# Pumping the brakes on RNA velocity by understanding and interpreting RNA velocity estimates

**DOI:** 10.1186/s13059-023-03065-x

**Published:** 2023-10-26

**Authors:** Shijie C. Zheng, Genevieve Stein-O’Brien, Leandros Boukas, Loyal A. Goff, Kasper D. Hansen

**Affiliations:** 1 Department of Biostatistics, Johns Hopkins Bloomberg School of Public Health, Baltimore, MD USA; 2 Department of Genetic Medicine, Johns Hopkins School of Medicine, Baltimore, MD USA; 3 Department of Neuroscience, Johns Hopkins School of Medicine, Baltimore, MD USA; 4 Kavli Neurodiscovery Institute, Johns Hopkins University, Baltimore, MD USA; 5 Quantitative Sciences Division, Department of Oncology, Johns Hopkins School of Medicine, Baltimore, MD USA

**Keywords:** Single-cell RNA sequencing, RNA velocity

## Abstract

**Background:**

RNA velocity analysis of single cells offers the potential to predict temporal dynamics from gene expression. In many systems, RNA velocity has been observed to produce a vector field that qualitatively reflects known features of the system. However, the limitations of RNA velocity estimates are still not well understood.

**Results:**

We analyze the impact of different steps in the RNA velocity workflow on direction and speed. We consider both high-dimensional velocity estimates and low-dimensional velocity vector fields mapped onto an embedding. We conclude the transition probability method for mapping velocity estimates onto an embedding is effectively interpolating in the embedding space. Our findings reveal a significant dependence of the RNA velocity workflow on smoothing via the k-nearest-neighbors (k-NN) graph of the observed data. This reliance results in considerable estimation errors for both direction and speed in both high- and low-dimensional settings when the k-NN graph fails to accurately represent the true data structure; this is an unknown feature of real data. RNA velocity performs poorly at estimating speed in both low- and high-dimensional spaces, except in very low noise settings. We introduce a novel quality measure that can identify when RNA velocity should not be used.

**Conclusions:**

Our findings emphasize the importance of choices in the RNA velocity workflow and highlight critical limitations of data analysis. We advise against over-interpreting expression dynamics using RNA velocity, particularly in terms of speed. Finally, we emphasize that the use of RNA velocity in assessing the correctness of a low-dimensional embedding is circular.

**Supplementary Information:**

The online version contains supplementary material available at 10.1186/s13059-023-03065-x.

## Background

RNA velocity analysis is widely used to infer temporal dynamics in single-cell gene expression data. In its original definition, RNA velocity is the time derivative of gene expression state (*ds*/*dt* with *s* representing the high-dimensional expression state and *t* time) [1]. Based on earlier work of Zeisel et al. [2], La Manno et al. [1] propose a model built using an ordinary differential equation model of transcription and the assumption that the relationship between spliced mRNA expression and unspliced pre-mRNA expression can be used to infer whether a gene is in the process of being upregulated or downregulated or in a steady expression state. Using this model, they derive an estimator for velocity using single-cell expression data. This model and estimation procedure was later extended by Bergen et al. [3]. These two variants are known as the steady-state model [1] and the dynamical model [3]. We return to these models in our results.

RNA velocity analysis for single-cell gene expression data starts with a high-dimensional estimation of *ds*/*dt*; we refer to these estimates as “velocities” (or gene-specific rates of change). In a second (optional) step, these high-dimensional velocities are visualized as a vector field on a low-dimensional embedding, usually constructed using UMAP or t-SNE. This vector field is supposed to represent local changes in the expression state; one might describe the output as an alternative to trajectory inference analysis [4]. We will use “velocity vector field” to refer to the low-dimensional vector field obtained by mapping the velocities into a low-dimensional space. In many datasets, the visualization of the velocity vector field appears to reflect what is known about the biological system and the relationship between cell states/types in the system.

Despite the widespread use and popularity of RNA velocity, studies that validate the high-dimensional RNA velocity estimates at the gene level are lacking. A significant reason is that measuring the instantaneous rate of expression change in a single cell is extremely hard. Qiu et al. [5] used metabolic labeling coupled with scRNA-seq to distinguish between newly synthesized and older mRNA. They directly compared the velocity estimates from metabolic labeling with the splicing-kinetics-based RNA velocity of 3 genes and found a poor correspondence between the two types of velocity estimates. It can be argued, however, that the explicit aspects of mRNA biogenesis estimated by metabolic labeling are subtly different from the models used in RNA velocity.

The potential of predicting the future state of individual cells has spurred tremendous interest in RNA velocity among the single-cell research community, resulting in many reviews and work building off the velocity framework. On the review side, Bergen et al. [6] highlights examples where the RNA velocity vector is not compatible with the known biology of the system and proposes that this is due to assumptions of multiple kinetic regimes, transcriptional boosts, high noise, or time-constant rates of transcription, splicing, and degradation. They further envision using gene regulatory networks and multimodal omics to expand RNA velocity models. Gorin et al. [7] is an in-depth discussion and critique of RNA velocity and complements the work we present here. The paper covers the underlying mathematics of RNA velocity in detail and advocates for a more rigorous approach to RNA velocity, respecting the discrete nature of transcription in single cells. Although the work covers all aspects of RNA velocity, there is a particular focus on the inherent discrete nature of the problem and its implications for preprocessing and biophysical modeling. The manuscript primarily discusses the Python implementation of velocyto.

While numerous tools seek to improve the RNA velocity framework, many methods build directly off the original framework. For example, Dynamo integrates metabolic labeling into the splicing-unspliced-dynamics-based model and tries to mathematically recover the whole velocity vector field in the low-dimensional embedding even for regions without any cells existing [8]. CellRank combines the k-NN graph built from the expression profile with the RNA velocity transition probability to detect initial, intermediate, and terminal populations during differentiation [9]. Marot-Lassauzaie et al. [10] first discusses several theoretical and computational problems and then proposes two alternative RNA velocity or RNA velocity flavored approaches. First, $\kappa$-velo tries to address the scale invariance problem by incorporating cell densities with the assumption that cell density is inversely proportional to the average velocity for each gene. Second, eco-velo is a heuristic RNA velocity-flavored approach that does not infer high-dimensional RNA velocity. Instead, eco-velo uses the first mutual nearest neighbor (MNN) of a cell in the unspliced matrix space to the spliced matrix space as the proxy for the future state in the spliced matrix space. A vector field is created based on the displacements between the future state and the current state in the spliced matrix space. Gao et al. [11] proposes a revised high-dimensional RNA velocities estimation model UniTVelo which imposes a gene-shared cell latent time to circumvent the independent estimation issue of other RNA velocity estimations approaches.

Here, we deconstruct the underlying workflow by separating the (gene-level) velocity estimation from the vector field visualization. We then analyze how the methods for mapping and visualizing the vector field impact the interpretation of RNA velocity and discover the central role played by the k-NN graph in both velocity estimation and vector field visualization. Using both simulations and real data, we identify situations where RNA velocity estimates are accurate and evaluate the extent to which the visualizations allow us to discover new structures in the data. We also explore whether―as their name suggests―velocity estimates can provide quantitative information about the speed at which cells progress along a trajectory.

## Results

### RNA velocity analysis and its implementations

RNA velocity analysis has two components, with the second component being optional

1. Spliced and unspliced counts are preprocessed (smoothed/imputed), and a cell-specific velocity is estimated separately for each gene.
2. (Optional) The high-dimensional velocity estimates are mapped into a low-dimensional embedding, and the resulting vector field is visualized on this embedding.

Visualization of RNA velocity results through the construction of a low-dimensional vector field on a given embedding (optional component 2) is important for using RNA velocity to bring insight into a biological system. Much―but not all―of the evidence for the usefulness of RNA velocity rests on its ability to produce low-dimensional vector fields which appear to represent known biology accurately. This suggests that it is important to understand the properties of this second component: what happens when high-dimensional velocity estimates are mapped to a low-dimensional embedding, particularly using the transition probability method introduced by La Manno et al. [1].

The two primary approaches to high-dimensional RNA velocity estimation are the steady-state model [1] and the dynamical model [3]. The main differences between the two are the specific assumptions about the parameters in the biophysical models of transcript abundance that are used to estimate cell-specific velocities (step 1 above), although many other differences impact the result (Supplementary Note 3.1). In the steady-state model, a single parameter representing degradation rate is assumed different between genes, and this parameter is estimated using robust regression [1]. In the dynamic model, both transcription, splicing, and degradation rate are assumed to differ between genes, and these parameters are estimated together with a cell-specific latent time for each cell using an EM algorithm [3]. The original papers contain well-written expositions of the details.

Here, we primarily focus on the newer dynamic model [3], as implemented in scVelo. We occasionally make comparisons to the steady-state model, using its implementation in the scVelo package (which we chose over other implementations of the steady-state model to ensure that we can keep other parameters of the workflow constant). Recent work has shown the importance of the choices of how to quantify spliced and unspliced counts [12]; here, we largely follow how each dataset was originally processed (Methods).

**Fig. 1:**
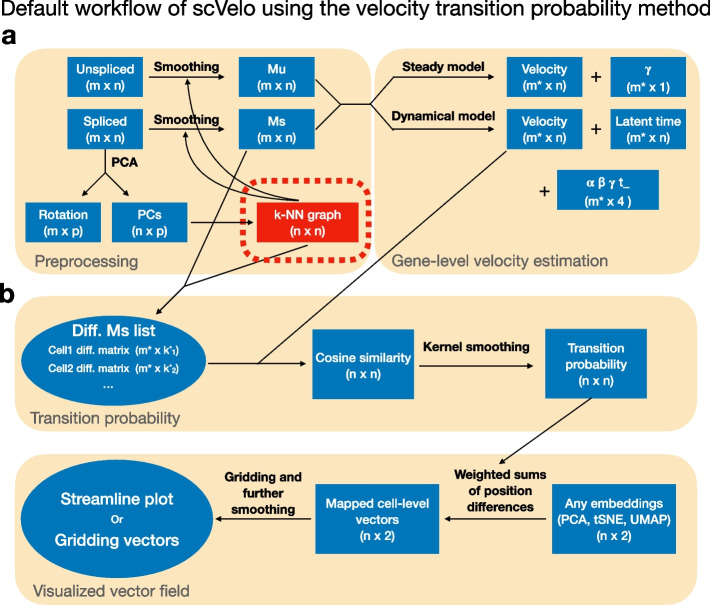
The flowchart of RNA velocity implementation in scRNA-seq data. The graph reflects scVelo. **a** A k-NN graph constructed from a PCA of the spliced counts is used to smooth (impute) the spliced and unspliced count matrices, resulting in the Ms and Mu matrix. This is followed by gene-specific velocity estimation using either the dynamical or the steady-state model. **b** Visualization of the estimated velocities on a low-dimensional embedding using velocity transition probabilities. First, transition probabilities are computed by considering which neighbors have a difference between the expression of the neighbor and the expression of the cell in question most similar to the estimated velocities. These transition probabilities are used to compute a vector as a linear combination of existing displacements. Finally, the resulting vector field can be visualized using streamline plots or a gridding approach (abbreviations: PCs, principal components; Diff. matrix, difference matrix between one cell to other cells)

The complete series of operations involved in an RNA velocity analysis workflow is outlined in Fig. 1 using the specific implementation for scVelo (note that Gorin et al. [7] has a similar velocyto-centric figure). We divide step 1 (inference of high-dimensional RNA velocity values) into two parts: preprocessing and gene-level velocity estimation (Fig. 1a). The raw count data is smoothed in the preprocessing step by a k-NN graph constructed exclusively from the spliced counts. A number of additional steps, such as library size adjustment and $\log$-transformation, are performed and discussed in Gorin et al. [7]. Following this, cell-specific velocities are estimated by fitting the smoothed spliced and unspliced counts to the appropriate biophysical model described by either the steady-state or dynamical model for each gene independently (Methods). Importantly, valid velocity estimations are only retained from a reduced set of genes where the model is considered well fit.

In the second step, after estimating the high-dimensional velocities, they are then visualized on a low-dimensional embedding, usually constructed using UMAP or t-SNE from principal components learned from the spliced count matrix. This consists of two steps (Fig. 1b). First, the velocity estimates are mapped into the existing low-dimensional embedding. The most common solution is to represent the velocities in this reduced dimensional space using transition probabilities, a method developed by La Manno et al. [1] and further modified by Bergen et al. [3]. After mapping, we are left with low-dimensional velocity vectors that are visualized using approaches such as streamline plots or gridding average vectors (Supplementary Note 3.2). In practice, this last visualization step almost always includes additional smoothing, such as kernel smoothing over the embedding; furthermore, the lengths of summarized vectors are usually rescaled.

Note there are many different terminologies related to visualization of the RNA velocity vector field. For example, Bergen et al. [3] used “project” to describe transforming the gene-level RNA velocity into an embedding. Here, we use “map” to describe the process of transforming the high-dimensional gene-level RNA velocities into the low-dimensional cell-specific vectors that can be visualized in the embedding space. And we reserve the word “project” to describe a mathematical projection onto a linear subspace. A large number of cells in most datasets implies that the final visualizations depict summarized/smoothed vector fields from the low-dimensional cell-specific vectors to avoid overplotting, and in that case “visualized” includes the summarization step.

Most attention in the literature has been given to a qualitative visual assessment of the RNA velocity vector field. We, therefore, start by examining how parts of the workflow impact visualization.

#### Mapping velocities into a low-dimensional embedding

To visualize scRNA-seq data on a nonparametric embedding, such as t-SNE or UMAP, mapping velocity estimates into an existing embedding is a nontrivial problem (unlike recomputing a new embedding based on the expression data supplemented with the predictions), and we briefly describe existing approaches to this problem. La Manno et al. [1] introduced a method we will refer to as “velocity transition probabilities” (later modified by Bergen et al. [3] which is the version we focus on here). This method is central to RNA velocity analysis since it is used in most RNA velocity vector field visualizations. The method also serves as the basis for the CellRank method for predicting fate specification [9]. Velocity transition probabilities are claimed to provide a solution to the mapping problem, which is compatible with *any* type of low dimensional embedding, including UMAP, t-SNE, and PCA, provided the embedding is constructed using expression data from the same cells yielding the velocity estimates. In addition to velocity transition probabilities, we have embedding-specific approaches such as an orthogonal projection operator for principal component plots and UMAP-transform for mapping into UMAP space (a method supplied by the UMAP authors) [13].

After estimating high-dimensional velocity by either the steady-state or dynamical model, we have the observed current expression state $\textbf{S}$ and the estimated velocity $\textbf{V}$ (both high-dimensional) (Methods). A first-order Taylor expansion of the expression state yields the following approximate relationship between current expression level $s_{gc}$, velocity $\vec {v}_g = d\vec {s}_g/dt$ and future expression level $s^*_{gc}$ for the cell *c* and the gene *g*:

$$\begin{aligned} s^*_{gc} = s_{gc} + v_{gc} \Delta t \end{aligned}$$

which requires a choice of time step $\Delta t$. For a fixed $\Delta t$, there is a one-to-one relationship between the velocity and the future expression.

We could use $E(\vec {y})$ to represent mapping a high-dimensional vector $\vec {y}$ to a low-dimensional space. We can map the velocities of cell *c* as

$$\begin{aligned} E^*(\vec {v}_c \Delta t) \equiv E(\vec {s}_c + \vec {v}_c \Delta t) - E(\vec {s}_c) \end{aligned}$$

Here, we are using $E^*$ to indicate that the left-hand side is a new operator defined by the right-hand side. If the mapping operator is linear (which is the case for principal component analysis), we get

$$\begin{aligned} E(\vec {s}_c + \vec {v}_c \Delta t) - E(\vec {s}_c) = E(\vec {v}_c) \Delta t \end{aligned}$$

and there is no difference between $E^*(\vec {v}_c)$ and $E(\vec {v}_c)$, and the impact of choosing a time step $\Delta t$ is just an overall scaling. If the mapping operator is not linear (which we believe is the case for UMAP-transform), there is a difference between $E^*(\vec {v}_c)$ and $E(\vec {v}_c)$, and the time step matters.

These computations require us to be able to compute $E(\vec {y})$ where $\vec {y}$ is an arbitrary new point in the expression space. The velocity transition probabilities were designed to work in cases where this is not directly obvious, as in the case of nonlinear embeddings. The only points we have available in the embedding space are the mapping of the observed cells, denoted as $E(\vec {s}_1), \ldots , E(\vec {s}_n)$. If we focus on a specific cell *i*, the idea is to represent the velocyto embedding as a weighted sum of empirical differences:

$$\begin{aligned} E^*(\vec {v}_i)&\equiv \, w_1(i) \big ( E(\vec {s}_1) - E(\vec {s}_i) \big ) + \cdots + \\&\quad w_n(i) \big (E(\vec {s}_n) - E(\vec {s}_i) \big ) \end{aligned}$$

for suitable choices of weights (depending on the cell in question). The weights are constructed to give higher weights to cells with a higher cosine similarity between $\vec {s}_j-\vec {s}_i$ and $\vec {v}_i$ (an approach that bypasses the choice of a time step $\Delta t$). In other words, you compute a future expression state in which high weights are given to cells with closer neighbors on the k-NN and to which you believe the cell is transitioning. In scVelo, this sum is restricted to include only terms from neighbors of neighbors of the cell *i* in the k-NN graph of the spliced counts. A consequence of this approach is that the direction of the mapped velocity must be towards the (convex hull) of neighbors of neighbors of the cell. This has the undesirable effect of assuring that the direction of the velocity vector for a given cell is entirely dependent on the expression states of its nearest neighbors, with consequences of constraining the vectors pointing to other existing cells around the query cell (detailed below).

### Transition probabilities impose directional constraints

**Fig. 2:**
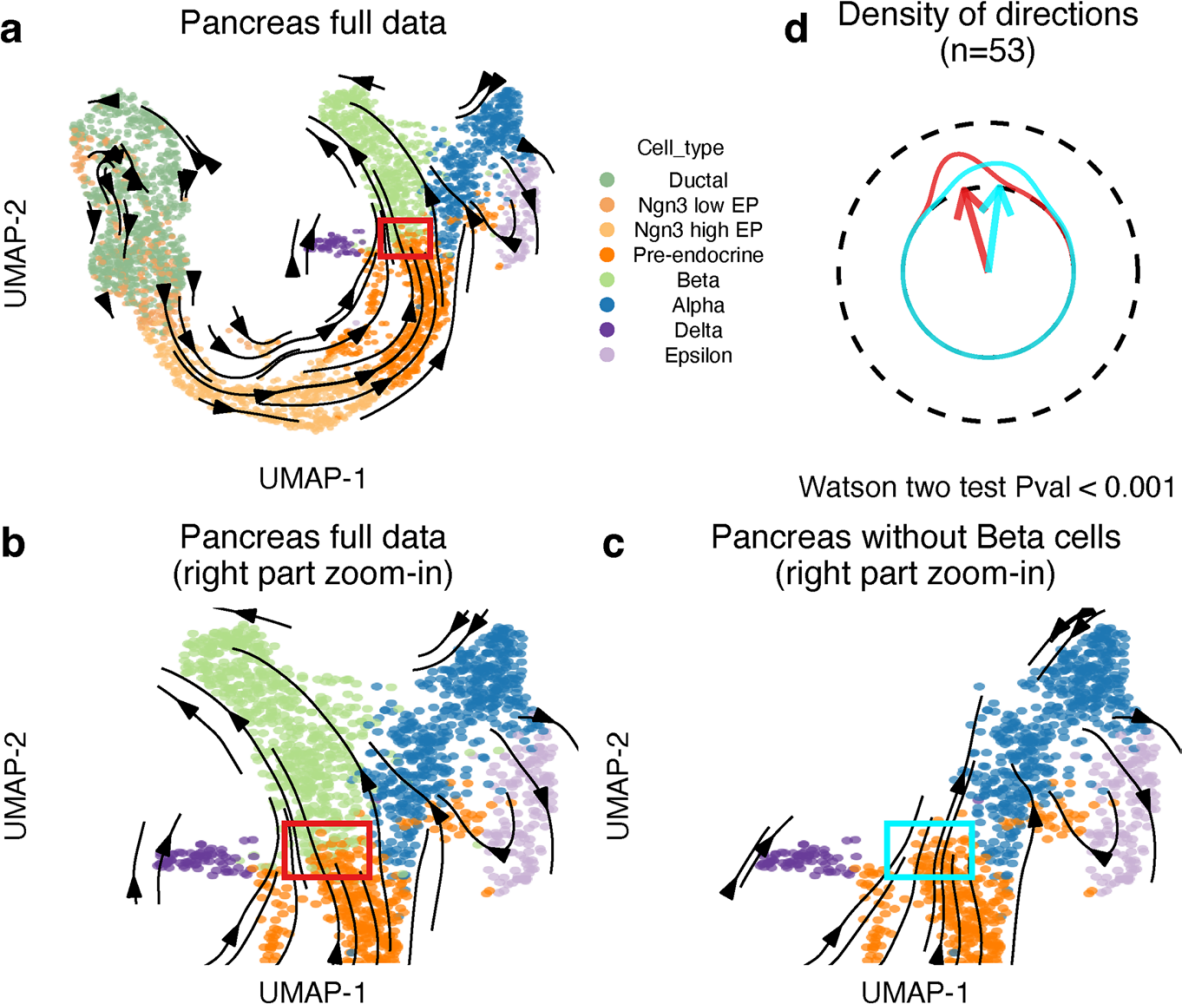
Mapped RNA velocity of the pancreas dataset. **a** Dynamical model-based RNA velocity mapped using velocity transition probability method for the full pancreas dataset. **b** Zoom in on the right part of **a**. **c** As **b**, but now we show the zoom-in for the pancreas data after removing the beta cells. Note that the gene-level velocity estimations are not changed, but the transition probability matrix is re-computed. **d** Comparison of directions of the cell-level vectors for the same set of pre-endocrine cells in the rectangle in **b** and **c** (abbreviation: Pval, *P*-value)

To illustrate the critical influence of neighboring cells on the resulting vector field, we consider the pancreas dataset featured in Bergen et al. [3] using the scVelo package for velocity estimates (Fig. 2a, b). As previously described, a standard velocity analysis suggests that pre-endocrine cells (orange) flow towards beta cells (light green) as expected. To demonstrate the dependence on nearest neighbor expression estimates, we then fix the high-dimensional velocity estimates (which are based on the full dataset) but remove the beta cells (light green) from the embedding step. Because we remove the beta cells, the recomputed velocity transition probabilities force the vectors to point to another part of the available embedding: the alpha cells (dark blue) (Fig. 2c and Additional file 1: Fig. S1a), resulting in a dramatic difference in the interpretation of the future state of the pre-endocrine cells. Figure 2d is a quantitative display of the change in low-dimensional vector directions for all cell-specific vectors of pre-endocrine cells in the red rectangle as a result of removing the beta cells from the embedding. We emphasize that the high-dimensional velocity estimates are unchanged between the panels.

This example illustrates a significant caveat of using transition probabilities for the low-dimensional estimation of velocity vectors. Velocity transition probabilities cannot represent unseen parts of an expression state space, regardless of the direction of the high-dimensional velocity vectors. Importantly, this suggests that the visualization of RNA velocities is more akin to an interpolation of observed expression states and less of an extrapolation of the future states. An example of this impacting data analysis is the comparison of multiple samples with sample-specific cell types or states, perhaps as a consequence of spatial heterogeneity (i.e., technical). In this case, the missing cell types or states will locally warp the vector field using transition probabilities.

### Visualizing velocity without noise

**Fig. 3:**
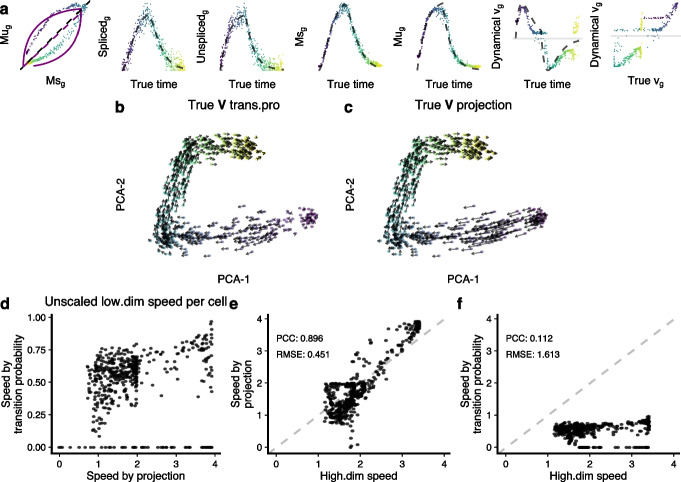
The challenges in visualizing RNA velocity in embeddings. **a** Example of a gene *g* in the simulated data. From left to right, we show the phase portrait, spliced counts over true latent time, unspliced counts over true latent time, Ms over true latent time, Mu over true latent time, estimated velocity over true latent time, and comparisons of estimated velocity and true velocity. **b** True RNA velocity vector field on PCA using the transition probability method. **c** As **b**, but the high-dimensional velocities are mapped by the projection method. **d** Comparison of the low-dimensional speed produced by two methods in **b** and **c**. **e** Comparison between the low-dimensional speed produced by projection and the high-dimensional speed. **f** Comparison between the low-dimensional speed produced by transition probability and the high-dimensional speed (abbreviations: trans.pro, transition probability; high.dim, high-dimensional)

To further examine the properties of velocity transition probabilities, we next employ a simulation experiment. We follow the simulation strategy of Bergen et al. [6] and generate 500 cells with 10 genes following the dynamic model with a low noise level. Figure 3a depicts one of these genes and shows that the dynamics of the simulated gene fit well with the dynamical model as expected. To ensure that we assess embedding and visualization separately from RNA velocity estimation, we use our simulation to define true velocities (defined as the velocity calculated with Eq. 2 with known parameters) and visualize these true velocities. While we use true velocities, the current expression state for each cell reflects measurement error according to our simulation.

We use principal component analysis to visualize the data because, for PCA, we have a mathematically well-defined and well-behaved projection operator which will respect the geometry of the high-dimensional vector field. The resulting embedding appears as a one-dimensional trajectory, reflecting the simulation strategy. The embedding has a small discontinuity around the start of the trajectory. Using velocity transition probabilities to map true velocities onto the PCA plot results in a vector field that flows in the right directory and reflects the simulation strategy (Fig. 3b,c, Additional file 1: Fig. S2). Using the PCA projection operator instead of velocity transition probabilities results in a qualitatively similar vector field.

We next sought to compare the vector field quantitatively cell-by-cell. The median of cosine similarities of cell-level vectors produced by transition probability and PCA projection is as high as 0.984, representing a high level of agreement in directions. We next compared speed (the length of the velocity vector; see the “Methods” section) between the two approaches. First, we compared the speed of the low-dimensional vector field resulting from transition probabilities to the vector field resulting from PCA projection. There is a low correlation between the speed of the two vector fields (Fig. 3d). Outliers are a small set of cells where the transition probabilities result in close to zero speed; these cells are all located at the start of the trajectory (Additional file 1: Fig. S2). More interesting is the relationship between the speed of the high-dimensional true velocity vector and its low-dimensional counterpart. Using PCA projection, we have a correlation of 0.9 (Fig. 3e). In contrast, the speed of low-dimensional velocities resulting from transition probabilities has no meaningful relationship with the speed of high-dimensional velocities (Fig. 3f).

In conclusion, in this simulation experiment, the different mapping approaches yield qualitatively similar vector fields with an exception around the start of the trajectory. Furthermore, for all simulated cells, there is little to no relationship between the speed obtained using velocity transition probabilities and the true high-dimensional speed. In contrast, using a PCA-based projection does a substantially better job of reflecting speed in high-dimensional space. We conclude that vector fields produced by transition probabilities do not reflect the high-dimensional speed.

### Velocity estimation and visualization are strongly dependent on the k-NN graph

**Fig. 4:**
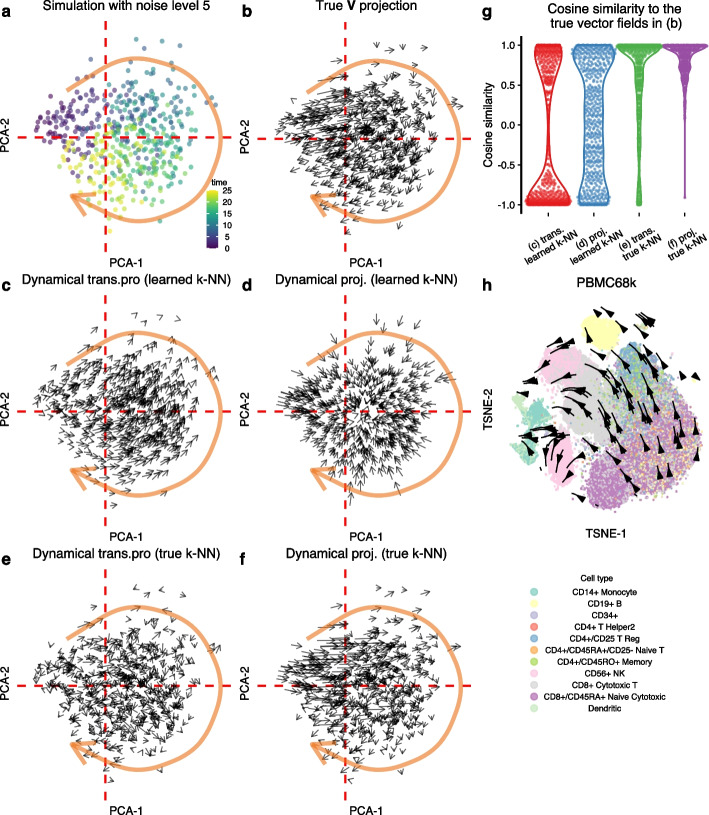
The RNA velocity vector field is dependent on the k-NN graph. **a** PCA of the simulated data. The dashed red line is an auxiliary line for mapping locations in other panels. **b** The PCA projections of the true RNA velocities. **c** RNA velocity inferred using the dynamic model and visualized using velocity transition probabilities (trans.pro). **d** RNA velocity inferred using the dynamic model and visualized using PCA projection. **e** As **c**, we use the true k-NN graph to both preprocess the data and as input to the velocity transition probabilities. **f** As **d**, but we use the true k-NN graph to preprocess the data. **g** The cosine similarities between the mapped cell-level vectors in **c**–**f** and the “true” mapped cell-level vectors in **b**. The usage of true k-NN substantially improves the cell-level vectors for both transition probability and direct projection methods. **h** The RNA velocity vector field of PBMC68k data flows with the embeddings, but it does not reflect the true biological trajectory. Each point represents a cell, which is colored by cell type (abbreviations: trans.pro, transition probability; trans., transition probability; proj., projection)

Single-cell data are known to be noisy. We next asked how increasing noise might affect low-dimensional vector field visualization. In the previous section, we examined issues with vector field visualization in a simulation setting with (unrealistic) low noise. When we use the same dynamics but increase the noise by 5x, the resulting PCA changes from a one-dimensional manifold to a ball (Fig. 4a). Different time points are located roughly in distinct quadrants of the ball, and the time progression is clockwise. A similar ball-like observation is made when we visualize the data using UMAP (Additional file 1: Fig. S3). Again, we project the true velocities using the PCA projection matrix, and the resulting vector field broadly reflects the time progression (as expected) (Fig. 4b). Note that neighboring vectors belonging to different quadrants point in very different directions, giving the impression of a very noisy visualization unless the true time progression is already known for reference. Notably, using a visualization method such as a streamline plot will have a smoothing effect that hides this behavior. We consider this vector field to be the gold standard for this embedding.

If we estimate velocities using the dynamical model and use the velocity transition probability method for visualization, we obtain a vector field with a smooth progression from left to upper right (Fig. 4c). Although visually pleasing (with apparent consistency in direction amongst neighbors), more than 50% of the vectors are strongly dissimilar from the gold standard (Fig. 4g). Note that this comparison is made at the cell level; the visualizations in Fig. 4 use the gridding approach. If we keep the velocity estimates from the dynamical model but map them using the PCA projection, we obtain a flow going from the edge of the circle towards its center (Fig. 4d), not reflecting the true time progression. Because neither visualization method compares favorably to the gold standard, it is tempting to conclude that this is a result of noise overwhelming the velocity estimates (we observe similar failures for the steady-state model in the Additional file 1: Fig. S4).

**Fig. 5:**
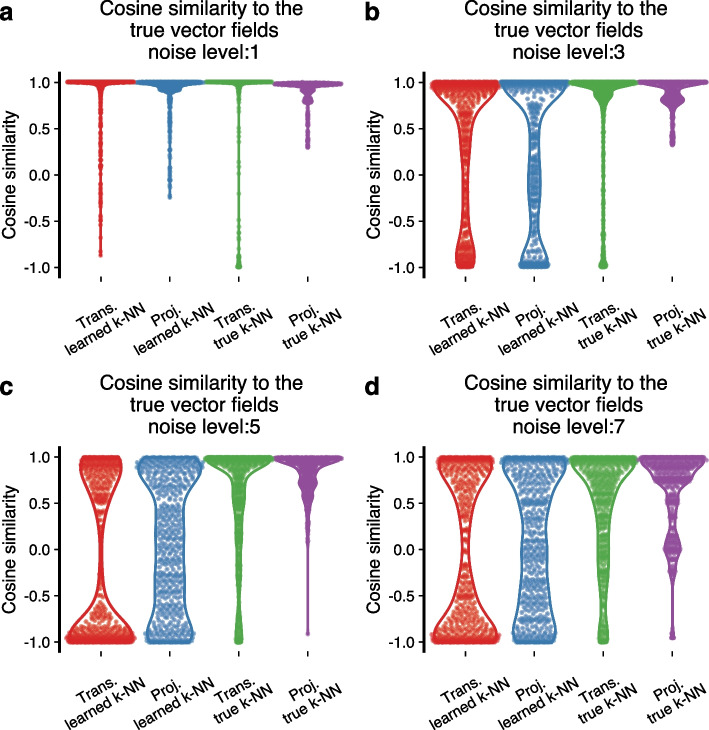
Cosine similarities between the mapped cell-level vectors and the “true” mapped cell-level vectors at various noise levels. Each panel resembles Fig. 4g, but we employ different noise levels during the simulations. Additional noise levels in Additional file 1: Fig. S5 (abbreviations: trans., transition probability; proj., projection)

However, using the appropriate k-NN graph is crucial for accurate inference. When we increase the noise level, two things happen simultaneously: gene-level measurements have added noise, and the k-NN graph learned from the data is perturbed away from the true graph. The significant change in the PCA layout reflects the latter. To investigate why the vector fields are incorrect, we obtain the true k-NN graph (the k-NN graph constructed using the spliced count matrix before adding noise) and use it to preprocess both the spliced and unspliced matrices, as well as to construct the velocity transition probabilities. Utilizing the true k-NN graph results in a vector field that is substantially more aligned with the gold standard. There is a slight improvement when using the PCA projection matrix compared to velocity transition probabilities (Fig. 4e–g). We reach the same conclusion when we use different noise levels for the simulations (Fig. 5, Additional file 1: Fig. S5). The gridding visualization of the velocity transition probabilities creates a noisy impression, which is not fully reflected by the cell-level comparisons in Fig. 4g.

Is the improvement in using the true k-NN graph driven by its usage in preprocessing or in mapping? When we attempt combinations of true and learned k-NN graphs for the two steps, we observe the most notable improvement by using the true k-NN graph in the preprocessing step (Additional file 1: Fig. S4).

We draw several conclusions from this example. First, it reveals that RNA velocity is critically dependent on the k-NN graph for vector field visualization. Second, we see how the true k-NN graph can be distorted by noise, an observation that is likely to be relevant outside of RNA velocity, given the importance of k-NN graphs in single-cell expression analysis. Third, we observe that the smoothness of the vector field does not imply correctness. Fourth, we are intrigued by the similarity of the “ball-of-cells”-like embedding to some existing single-cell embeddings: we refer to embeddings that show a dense structure where different parts of the structure appear to consist of distinct cell types. An example is the data depicted in Fig. 4h, which is comprehensively discussed in Bergen et al. [6] as an example where the RNA velocity fails. Our simulation experiment suggests this could arise primarily from noise deforming the k-NN graph.

### Evaluating gene-level RNA velocities using simulations

**Fig. 6:**
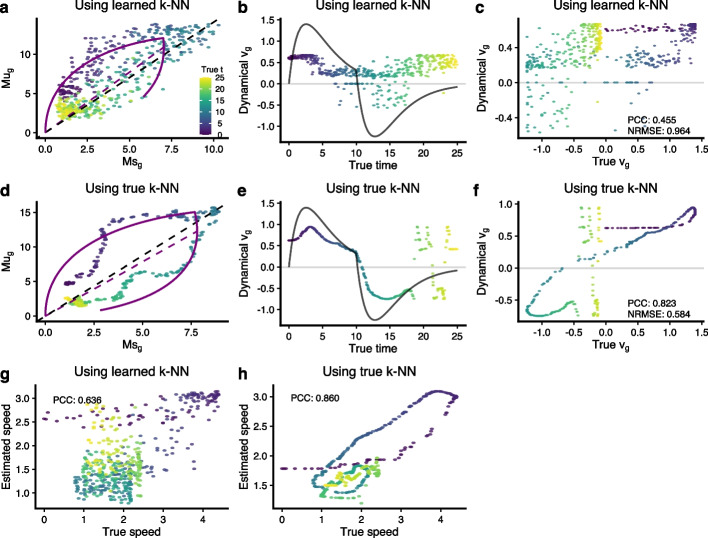
Gene-level RNA velocity estimation depends on the underlying k-NN graph. In all panels, a data point represents a cell and is colored by the known true latent time *t*. All solid black lines represent the known true values. **a** Phase portrait shows the Ms over Mu using the learned k-NN. The parameters are estimated by the dynamical model. **b** Estimated velocity (points) using the learned k-NN and true velocity (black line) over true latent time *t*. **c** Scatter plot compares the estimated velocity values (using the learned k-NN) to the true velocity values. Pearson’s correlation coefficient (PCC) and normalized root mean square error (NRMSE) are given. **d**–**f** As **a**–**c**, but now we use the true k-NN to get Ms and Mu matrices. The estimated velocity values are much closer to the true velocity values with PCC 0.823 and NRMSE 0.584. **g** Scatter plot compares the true (high-dimensional) speed to that of the estimated (high-dimensional) speed by the dynamical model using the learned k-NN graph. **h** As **g**, but we use the true k-NN to infer (high-dimensional) velocity (abbreviations: PCC, Pearson’s correlation coefficient; NRMSE, normalized root mean square error)

So far, we have focused on the low-dimensional velocity vector field on a fixed embedding. It is natural to ask to what extent the gene-level estimates are accurate. To do so, we turn to our simulations. Using a noise level of 3, the topology of the PCA plot is a 1-dimensional trajectory (Additional file 1: Fig. S6) with some noise. Using a k-NN learned from the data, the estimated velocities are quite inaccurate when considered as a function of the true time (Fig. 6a–c, Additional file 1: Fig. S7 for additional quantities). When switching to the true k-NN graph, the dynamical model works substantially better. However, there is still some discrepancy between the true velocity and the estimated velocity (Fig. 6d–f). Additional file 1: Fig. S7 expands on the quantities depicted in Fig. 6, and Additional file 1: Fig. S8 depicts the situation when we increase the noise level to 5, the situation where the PCA plot changes from a 1-dimensional trajectory to a ball.

Additional file 1: Fig. S9a,b depicts a comprehensive evaluation across all genes and across many noise settings for both the dynamical and the steady-state model. As the noise level increases, the dynamical model cannot be fitted to most of the genes, and the performance measures are restricted to the few genes that provide a passable fit (as determined by the software). We make several observations. First, the performance is markedly better when using the true k-NN graph compared to the learned k-NN graph. Second, the steady-state model performs better when the assessment criteria are independent of scale (such as PCC), whereas the dynamical model performs better when the assessment criteria are scale-dependent (such as NRMSE). At very low noise settings (1 or 2), RNA velocity appears to work well; using the true k-NN graph still improves performance substantially.

Aside from comparing the velocity values gene-by-gene, we can compare the speed and direction of the high-dimensional velocity vector cell-by-cell. Using a noise level of 3, we find poor concordance between the estimated true speed when using the learned k-NN (Fig. 6g). This is substantially improved by using the true k-NN (Fig. 6h). The steady-state model shows a similar behavior (Additional file 1: Fig. S10), although the scale of the speed is wrong. We can compare the speed estimates with the truth using both absolute differences (a measure dominated by the scale) and Pearson correlation (unaffected by the scale) (Additional file 1: Fig. S11a,b). Together, these two measures reveal that the estimates of speed from both the dynamical and the steady-state models fail to reflect the truth, at least when the learned k-NN graph is used. Direction is harder to assess in high dimensions; the cosine similarity is a step toward this goal, and it suggests poor concordance between estimated and true directions when using the learned k-NN graph (Additional file 1: Fig. S11c). Using the true k-NN graph leads to substantial performance improvements in estimating both speed and direction, but the overall performance is still poor for high noise levels. For low noise levels (1 or 2), we observe a good PCC between estimated and true speed as well as good-to-decent cosine similarity between directions.

In conclusion, the gene level RNA velocity estimations are highly dependent on the k-NN graph used to smooth the data. Even in a simulated setting where the observed k-NN graph reflects the true underlying structure (the PCA plot shows a 1-dimensional trajectory), using the observed k-NN graph results in substantial errors in estimated velocities.

### Correct estimate of the vector field does not imply accurate high-dimensional velocity estimation

Our ability to assess RNA velocity is limited by the technical difficulties in measuring the instantaneous rate of change in expression for many genes in single cells. To evaluate the RNA velocity estimates in real data, we consider a recent dataset on the cell cycle measured using the FUCCI system combined with scRNA-seq [14]. The cell cycle is a well-understood periodic process [15], where cell cycle-related genes go through phases of upregulation and downregulation. In this dataset, we can place each cell in a continuum representing the cell cycle. One approach to this goal is to take advantage of the FUCCI system, which tracks cell cycle time by the protein levels of two key cell cycle regulators, CDT1 and GEMININ. However, in our recent work, we have established that it is possible to improve this cell cycle time by projecting this data into a low-dimensional space representing the cell cycle [16]. We refer to these two time representations as the tricycle cell cycle time (position) and the FUCCI pseudotime.

First, we note that cell cycle genes have a specific role in RNA velocity estimation. Due to the periodicity, cell cycle genes go through both an upregulation and a downregulation phase, but, perhaps surprisingly, they dominate among such genes in existing single-cell datasets. In practice, this suggests that dynamically regulated cell cycle genes *should* sample both the increasing and decreasing portions of the RNA velocity biophysical models, providing for a potential better fitting of these models. Indeed, if we look at the top ten best-fitting genes across ten different single-cell expression datasets, the *only* genes having a “complete” phase portrait are cell cycle genes (Additional file 1: Fig. S12). Non-cell-cycle genes are either in the induction or repression phase. For this reason, we believe that cell cycle datasets such as the FUCCI dataset we consider here are amongst the most suitable for RNA velocity analysis using velocity estimates derived from current biophysical models.

**Fig. 7:**
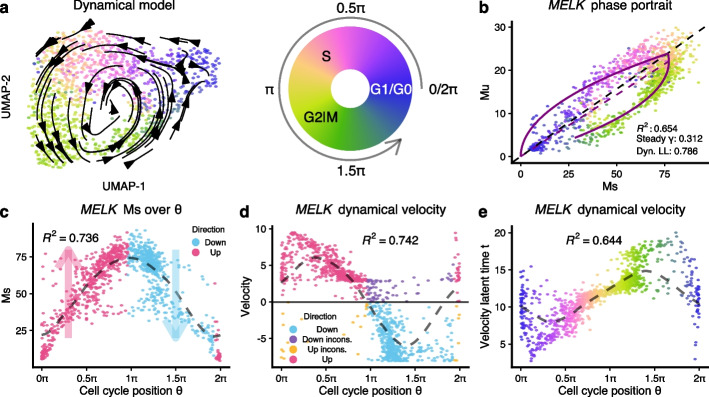
The RNA velocity application on FUCCI dataset. **a** RNA velocity vector field is visualized using the transition probability method on the UMAP embeddings of the FUCCI data. Each point represents a cell and is colored by the tricycle cell cycle position. **b** Phase portrait of gene *MELK*, of which the likelihood is the highest among all velocity genes inferred by the dynamical model. The purple lines represent the dynamics inferred by the dynamical model. **c** Scatter plot shows smoothed expression of *MELK* over cell cycle position. The dashed line is the fitted line by periodic loess (Methods). The expected direction of change is inferred on the fitted loess line and visualized by colors. **d** Scatter plot shows the estimated RNA velocity of *MELK* over cell cycle position. The signs of velocity estimations are compared to those inferred in **c**, with inconsistent directions colored purple/orange. The variance explained by cell cycle position ($R^2$ in the figure) is comparable for velocity values compared to Ms in **c**. **e** The velocity latent time for *MELK* generally agrees with cell cycle position, except for cells around G1 or G0 phases (abbreviation: incons., inconsistent)

Using the FUCCI data, the visualized vector field reflects cell cycle time (Fig. 7a) and so does the phase plot of the 10 best-fitting cell cycle genes (Fig. 7b and Additional file 1: Fig. S13).

Using our estimated cell cycle time from the tricycle, we can define the upregulation and downregulation phases for each gene by smoothing expression using the cell cycle time loess fitted line (Fig. 7c). Comparing these phases with the sign of the estimated velocities using the dynamical model reveals a high concordance (Fig. 7d). A feature of the dynamical model is the estimate of a cell-specific latent time which has good concordance with cell cycle time for *MELK* (Fig. 7e). Here, we use the gene-specific latent time, which is different from the gene-shared latent time[3].

Using the steady-state model, we get essentially the same vector field (Additional file 1: Fig. S14a). However, there are non-negligible inconsistencies between gene-level RNA velocity estimations from the steady-state and the dynamical model. For example, for the *MELK* gene $24\%$ cells exhibit different directions of change (Additional file 1: Fig. S14b-c) between the two models; the dynamical model agrees better with the direction of change inferred using cell cycle position. For each gene, we compute the PCC between the estimated velocities from the two models as well as the number of cells with inconsistent directions (Additional file 1: Fig. S14d). Across genes, the median PCC between the models is only 40%, with 30% of cells showing an inconsistent direction of change. Intriguingly, the two models yield qualitatively similar vector fields despite the substantial differences in estimated velocities. This, again, raises the issue of to what extent the velocities actually impact the vector fields. We emphasize that this comparison only focuses on the direction of change and not the rate of change.

If we take all the cycle genes with a $R^2\ge 0.5$ for Ms over the cell cycle position, we observe that the dynamical model is more consistent in the inference of velocity direction (Additional file 1: Fig. S15).

Using the tricycle cell time can be criticized because it is inferred using the expression data. If we replace tricycle cell cycle time with FUCCI pseudo-time, we observe a higher degree of discordance with FUCCI pseudo-time (Additional file 1: Fig. S16) compared to tricycle time. Additional file 1: Fig. S17 shows ten additional genes with similar behavior.

In summary, when it comes to the inference of direction of expression change, RNA velocity appears to work well in this cell cycle system, likely due to the fact that dynamic genes in this system experience both inductions and decrease over the course of the cell cycle. We note the unique role of the cell cycle in velocity analysis: the periodic nature of the cell cycle fits well with the biophysical model of transcription, and cell cycle genes are often among the best-fitting genes across biological systems. As a result, the velocity vector field is correct, and the direction of change of the gene-level velocities is largely correct for the dynamical model. We find it noteworthy that the steady-state model also yields a correct velocity vector field despite the two models yielding high-dimensional velocity estimates with substantial inconsistencies. Based on this, we believe the cell cycle represents a system that is particularly well suited to RNA velocity, and we caution against generalizing its performance to other systems.

### A quality control measure for RNA velocity model

**Fig. 8:**
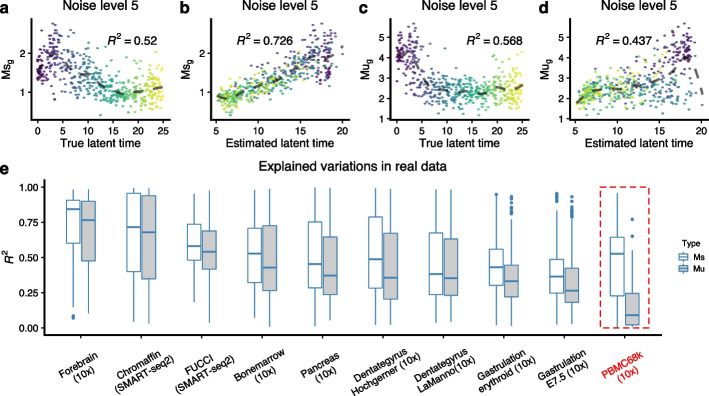
Using the $R^2$ values to detect bad datasets with high noise. **a**–**d** Using a gene from the simulation at noise level 5, we show Ms over true latent time, Ms over estimated latent time, Mu over true latent time, and Mu over estimated latent time. The dashed lines are fitted loess, on which the $R^2$ values are calculated (Methods). **e** $R^2$ values of Ms and Mu over the estimated latent time for the top 300 velocity genes ranked by the likelihood in 10 real datasets. Note that in the PBMC68k dataset, where we know the RNA velocity vector field does not work as we expect, the median $R^2$ for Mu is less than 0.1, and the difference between the median $R^2$ for Mu and Ms is about 0.5

A central question is when to trust RNA velocity vector fields. To assess this, we focus on how well the estimated latent time explains variation in the expression estimates. In the dynamical model, we estimate a gene-specific latent time representing the unknown time parameter in the differential equations; this quantity is unavailable from the steady-state model. In a subsequent step, the gene-specific latent time is summarized into a gene-shared latent time by taking the quantile (across genes) of the estimated gene-specific latent times. We ask how much variation in expression is explained by a loess fit on the gene-specific latent time (Fig. 8a,b). As an example, for one specific gene, for simulated data, we observe $R^2=0.73$ using the estimated latent time compared to $R^2=0.52$ using the true time. This suggests some degree of overfitting, possibly caused by either the preprocessing step or the model fit itself. Fortunately, in addition to the spliced counts, we also get unspliced counts, which are also smoothed but using the k-NN learned from the spliced counts. By comparing the difference between the spliced and unspliced expression matrices as a function of estimated latent time or the true time (Fig. 8c,d compared to Fig. 8a, b), we conclude that the source of the overfitting is the preprocessing step of smoothing using the spliced k-NN graph. We argue that this can be avoided by using the unspliced instead of the spliced matrix to assess how well RNA velocity model fitting works across genes because the preprocessing is done using the spliced k-NN graph and not the unspliced.

We applied this to the top 300 genes (ranked by the likelihoods of the dynamical model) in ten datasets (Fig. 8e). The PBMC68k data―highlighted by Bergen et al. [6] as a dataset where RNA velocity fails―is an outlier in this display, with a very high discrepancy between $R^2$ for the spliced and unspliced expression values and a very low $R^2$ for the unspliced values. The two gastrulation data sets that have the second and third lowest $R^2$ values have been reported to show unexpected directions in their RNA velocity vector fields that oppose the true differentiation path [17]; the authors speculate that these inconsistencies are driven by time-dependent changes in expression dynamics. We interpret a small value of $R^2$ for the unspliced matrix as evidence of a poor fit of the RNA velocity model. And we interpret a discrepancy between the $R^2$ of the spliced and unspliced matrix to be evidence of overfitting arising from the k-NN smoothing. Altogether, this suggests that our measure has some functionality in the sense that extreme behavior on this measure should inspire low confidence in the vector fields.

## Discussion

In physics, velocity is the combination of direction and speed and is defined as $v = ds/dt$, the derivative of position *s* with respect to time *t*. RNA velocity promises to bring temporal dynamics into gene expression analysis by estimating the derivative of the expression with respect to time. When assessing this promise, it is important to separately consider direction and speed (the length of the vector). For both direction and speed, RNA velocity produces two quantities: high-dimensional velocity vectors (each entry corresponds to a gene) and representations of these vectors in a suitable low-dimensional space, such as a UMAP embedding. We remind the reader that we use the term “velocity vector field” exclusively to refer to the low-dimensional representation. This vector field is always relative to the choice of low-dimensional space and is easy to visualize.

### Why does RNA velocity appear to work?

The low-dimensional directions inferred by RNA velocity appear to be successful at describing known biology in many systems. “Describing known biology” is a qualitative statement reflecting that the visualized vector field reflects known progression through a system; an example is the pancreas dataset (Fig. 2). We emphasize that this qualitative assessment is always exclusively in reference to the direction of the vector field; we will discuss speed below.

Most single-cell expression analyses―and all existing RNA velocity analyses―start by constructing a low-dimensional embedding (usually) using UMAP. It is an open question under which conditions UMAP is ever guaranteed to reflect biological truth. Despite this lack of guarantees, it is indisputable that UMAP often achieves a representation that qualitatively reflects existing knowledge (related cell types and states are placed close together). We argue that any successful RNA velocity analysis starts with a UMAP, which is deemed to represent existing or anticipated knowledge about the system. RNA velocity then produces a vector field overlay on this low-dimensional representation.

Here, we show that the direction of RNA velocity is strongly determined by the observed k-NN graph of the data. This k-NN graph is also directly reflected by the UMAP layout (indeed, one can view a UMAP layout as a representation of the k-NN graph). Together, this guarantees a *compatibility* between the UMAP and the vector field: vectors will always point towards neighbors on the UMAP. Importantly, this occurs regardless of whether the UMAP is in some sense “true.” Therefore, the directions inferred by RNA velocity cannot be interpreted as additional evidence for the correctness of the UMAP. For example, it is impossible for the RNA velocity vector field to construct a vector showing a transition between two cell populations that are distant on the UMAP. Instead, RNA velocity is more similar to a smoothing of the k-NN (and therefore UMAP) structure and cannot reveal a new structure but only depict a structure already present in the UMAP.

Our various experiments support these statements about the direction of the RNA velocity vector field; we next discuss these points and the experiments in detail.

### What influences the direction of RNA velocity?

First, we discuss the low-dimensional velocity vector field and focus on the particular case where mapping is done using velocity transition probabilities, as is common when visualizing RNA velocity on a UMAP, t-SNE, or other nonlinear embeddings. We show that the resulting vector field is strongly dependent on the structure of the k-NN graph (Fig. 4). The use of the k-NN graph to preprocess (smooth) the spliced and unspliced matrix has a larger impact on the resulting vector field than the use of the k-NN graph to estimate velocity transition probabilities (Additional file 1: Fig. S4). Additionally, we show how the resulting vectors are constrained to point exclusively toward sampled cell locations (Fig. 2).

Second, we consider the direction of the low-dimensional RNA velocity vector field when the UMAP and k-NN graphs are perturbed away from the “true” structure. It is not obvious how to define the true structure. Here, we use the fact that we are simulating from a model which can be represented as “signal + noise” where “noise” is independent Gaussian (Methods). When using the term “true” k-NN graph, we generate the signal data and form a k-NN graph of the signal data prior to adding independent noise. Our simulation results show that increasing noise can significantly perturb the observed (“learned”) k-NN graph away from the true neighborhood structure. This is best visualized by comparing the UMAP layouts at different noise levels (Additional file 1: Fig. S3). While our observation is based on a specific simulation result, we hypothesize that this is a common phenomenon in single-cell expression data, where technical noise can be the greatest source of variation. In our simulations involving moderate to high noise levels, we find that a genuine one-dimensional manifold is depicted as a compact cluster of cells in PCA or UMAP embeddings. Despite the noise, the general organization of the quadrants of the mass still corresponds to specific time points, conditions, or cell states, as illustrated in Fig. 4 and Additional file 1: Fig. S3. Because specific cell states appear to occupy distinct regions of the dense mass of cells, and related cell types/states are placed close together, this is often interpreted as a signal in the data. Our simulations suggest that such a dense mass, caused by the noise of the data, might obscure a simple trajectory. In this scenario, RNA velocity produces a pleasing, smoothed, low-dimensional velocity vector field, which nevertheless is a wrong representation of the structure of the system (Fig. 4). Given the importance of the observed k-NN graph in many methods of single-cell expression analysis, this observation may have far-reaching implications beyond RNA velocity analysis.

We now turn to the direction in high-dimensional space. First, we consider the relationship between high-dimensional velocities and the low-dimensional vector field. In our analysis of the FUCCI data, we show that both the dynamical model and the steady-state model yield overall similar vector fields but that the inferred direction of change of expression is different between the two models (Fig. 7 and Additional file 1: Fig. S14). This highlights that different high-dimensional velocities can result in the same low-dimensional vector field and cautions us against using the low-dimensional vector field to draw conclusions about the high-dimensional velocities. In our simulations, we observe that the estimated high-dimensional velocities strongly depend on the k-NN graph. Using the observed k-NN graph, there is a little concordance of directions between true high-dimensional velocities and the high-dimensional velocity estimates (Additional file 1: Fig. S11) at medium to high noise levels. At low noise levels (1-2), the cosine similarity between estimated and true directions is relatively high. Together, this suggests that high-dimensional velocities can be highly misleading. We note that high-dimensional velocities are not directly used in most RNA velocity analyses.

### RNA velocity does not estimate expression speed

We now turn our attention to expression speed: the length of the $v = ds/dt$ vector. Speed is seldom directly analyzed, but precocious differentiation in disease may result from changes in speed. Again, there is a speed of the high-dimensional velocity and a speed of the low-dimensional velocity resulting from mapping into a low-dimensional embedding.

We show conclusively that there is a little-to-no relationship between high-dimensional speed and speed in the low-dimensional velocity vector field when velocities are mapped using transition probabilities (Fig. 3). This suggests that speed in the low-dimensional embedding is meaningless (in this scenario). This is explained by considering the velocity transition probabilities: high-dimensional speed (vector length) is simply not used to determine the low-dimensional speed. The consistency between high- and low-dimensional speed is substantially better when velocities are projected onto a PCA plot using an orthogonal projection operator. However, it is well appreciated that PCA plots in 2 dimensions regularly only capture part of the multiple biological processes co-occurring in single-cell data.

Our simulation experiments show that high-dimensional speed estimates have poor concordance with the true speed at medium to high noise levels, although the concordance can be improved by using the true k-NN graph (Fig. 6 and Additional file 1: Fig. S10, S11). However, since the true k-NN graph is always unknown, it is of limited practical utility beyond the illustration of discordance. At low noise levels (1-2), there is a decent correlation between the true speed and the estimated speed.

In summary, there is no reason to believe that current RNA velocity workflows are capable of estimating gene expression speed, neither in high nor low dimensions. We make two comments related to speed. First, there is a significant gap in the ability to validate the speed of global gene expression transitions experimentally. If the accurate estimation of speed is of interest, it is critical to developing experimental approaches that will directly measure this property to provide a ground truth against which validation is possible. Second, there are issues with the theoretical definition of the speed of gene expression changes. When computing the speed, coordinates (genes) contribute with equal weight. This makes sense in 3D physics but is less intuitively clear in expression analysis: for example, one might want to consider the expression level and intrinsic variation of each gene. In practice, we believe that a more useful concept of speed is one that is coupled to specific biological processes, such as cell cycle speed, differentiation speed, or transcriptional response to perturbation.

#### The impact of the noise level in our simulations

 We have utilized simulations to examine the performance of RNA velocity under varying noise levels and compared the results of using both the learned and true k-NN graphs. As these simulations are based on the RNA velocity model, they represent an idealized best-case scenario. With a fixed signal in these simulations, they correspond to decreasing signal-to-noise situations. Our findings unequivocally demonstrate that the signal-to-noise ratio significantly influences RNA velocity performance. A critical aspect of this impact arises from the k-NN graph of the observed data, particularly through the k-NN smoothing step implemented during preprocessing. In low noise regimes (levels 1–2), RNA velocity exhibits decent to good performance. However, its effectiveness quickly deteriorates at higher noise levels, where the learned k-NN graph deviates from the k-NN graph calculated on data before the addition of measurement errors. For noise levels 1–2, the measurement noise itself is unrealistically low (Fig. 3a). Nonetheless, the crucial factor is likely the noise magnitude relative to the signal, which we have not attempted to quantify in real data. Quantifying this aspect is challenging because it depends on the magnitude of structured expression changes across the cell population being investigated. Consequently, we believe that determining how to quantify signal-to-noise in single-cell data and examining its impact on the learned k-NN graph is an essential open question. This inquiry is particularly significant since the k-NN graph is a central component in many single-cell analysis methods.

#### Limitations of our simulations

Following Bergen et al. [6], we use a straightforward simulation strategy where all genes are velocity genes, and the dynamics fit the underlying ODE perfectly well. Furthermore, the gene-specific parameters are identical between the genes. This is highly unrealistic but provides an over-optimistic best-case scenario. Given the model failures in this simulation setup, we would expect even larger discrepancies with more real-world models. We have criticized―but not critically evaluated―the aggregation of gene-specific latent times into a cell-specific latent time. Because the simulation setup imposes the exact ordering of cells for every gene, the aggregation step works well. To investigate issues with inferring a cell-specific latent time, we suggest one would need a simulation design where multiple processes are happening across multiple time scales, for example, cell cycling and differentiation happening at the same time.

#### The exception that proves the rule: the FUCCI data

 Our analysis of the FUCCI data adds to the short list of attempts at validating RNA velocity using experimental data. We take advantage of cell cycle time (a useful time concept different from wall time). Using this approach, we find the relatively good performance of the dynamical model on cell cycle related genes in predicting the direction of gene expression changes (Additional file 1: Fig. S15). Note that this is a partial validation: we are only considering the direction of change of expression (whether a gene is upregulated or downregulated) and not whether the “speed” or the predicted new state is correct. There is a substantial discrepancy between the dynamical and the steady-state model, and both models arrive at the same vector field.

In our work, we have substantially criticized RNA velocity. Why do we maintain our criticism in light of our moderately successful validation attempt? We believe that the FUCCI data is a rare experimental system that fits the underlying RNA velocity model well. First, only a single underlying process is happening (cell cycle). This is a substantial simplification compared to many datasets where multiple processes occur simultaneously. Second, gene expression in this process follows a cyclic pattern with both upregulation and downregulation. This is in contrast to other processes, such as differentiation, in which most dynamic genes are either exclusively downregulated or upregulated along the process. An example of such a system is red blood cell development, where the regulation of some key genes, such as hemoglobin genes, is monotonic [18]. Interestingly, RNA velocity was recently reported to result in a reverse (wrong) direction in this system due to errors in assigning the correct expression phase (up/down) to key genes [17]. Third, the UMAP suggests that the observed k-NN graph accurately reflects the underlying biology. Our criticism of RNA velocity is about the use of the method to “validate” a given embedding, and the FUCCI data does not really address this problem since we know the initial UMAP accurately reflects cell cycle progression.

## Conclusions

In light of our results, we believe that RNA velocity has far from achieving its stated goal: quantifying expression dynamics. Indeed, most applications of RNA velocity to date have exclusively relied on a qualitative interpretation of RNA velocity vector field estimates to “reinforce” the validity of learned trajectories in a reduced dimensional embedding. We provide evidence here that this validation exercise is, at best, a circular logic and, at worst, potentially inaccurate and misleading. The promise of RNA velocity as a quantitative tool to examine expression dynamics further falls short when the validity of these estimates is explored. Speed is especially problematic and has received little attention in the literature. At its best, RNA velocity provides a potentially useful visualization tool, conceptually similar to pseudo-time ordering.

## Methods

### Review of the dynamical model of RNA velocity for scRNA-seq data

RNA velocity is usually introduced through a pair of differential equations for the amount of spliced and unspliced RNA depending on the cell-specific latent time *t* for each gene independently [3]:

1 $$\begin{aligned} \frac{d u(t)}{dt} = \alpha ^ {(k)} - \beta u(t). \end{aligned}$$

2 $$\begin{aligned} \frac{d s(t)}{dt} = \beta u(t) - \gamma s(t). \end{aligned}$$

There are three unknown (constant) parameters for each gene: the transcription rate ($\alpha$), the splicing rate ($\beta$), and the degradation rate ($\gamma$). Unlike the steady-state model, which only searches for the degradation rate $\gamma$, the dynamical model solves for the three parameters. The analytical solutions to Eqs. 1 and 2, as given in Bergen et al. [3], are the key parts for the estimation of the parameters:

3 $$\begin{aligned} u(t)&= u_0 e^{-\beta \tau } + \frac{\alpha ^{(k)}}{\beta } (1 - e^{-\beta \tau })\nonumber \\ s(t)&= s_0 e^{-\gamma \tau } + \frac{\alpha ^ {(k)}}{\gamma } (1 - e^{-\gamma \tau })\nonumber \\&\quad + \frac{\alpha ^ {(k)} - \beta u_0}{\gamma - \beta } (e^{-\gamma \tau } - e^{-\beta \tau })\nonumber \\ \tau&= t - t^{(k)}_0 \end{aligned}$$

Here, $u_0$ and $s_0$ denote the initial unspliced and spliced counts, which are both set to 0 as default in implementation. And $\tau$ is the difference between the latent time *t* and the time point at which the phase change occurs. The greatest advancement of the dynamical model is that we get the cell-specific latent time *t* of each gene. Calculating the cell-specific velocity is performed by inserting the calculated values of *u*(*t*) and *s*(*t*) into the Eq. 2 instead of taking the residuals of the quantile regression as done by the steady-state model. We note that one of the important characteristics of the equation system 3 is that both *s*(*t*) and *u*(*t*) are univariate functions of cell-specific latent time *t* given all estimated parameters. It follows that Eq. 2 can also be expressed as a univariate function of the cell-specific latent time *t*.

### Real scRNA-seq datasets filtering and normalization

All real scRNA-seq datasets we used are summarized in Table 1. We got the Forebrain data [1], the Bonemarrow data [19], the Dentategyrus LaManno data [1, 20], the Pancreas data [3, 21], the Gastrulation erythroid data [22], the Dentategyrus Hochgerner data [3, 20], the Gastrulation E7.5 data [22], and the PBMC68k data [23] from the scVelo package directly (https://scvelo.readthedocs.io/api/#datasets). We got the Chromaffin data [1, 24] from http://pklab.med.harvard.edu/velocyto/notebooks/R/chromaffin2.nb.html and the FUCCI data [14] from https://drive.google.com/file/d/149ICTtieYjuKWZoLwRLzimwff0n6eWqw/view?usp=sharing. Usually, we did the following procedures for all real datasets: we first filtered genes with more than 20 counts across cells in both spliced and unspliced count matrices, and we only retained cells with more than 200 counts across genes in both spliced and unspliced count matrices; the filtered spliced count matrix was library size normalized across cells and $\log _2$ transformed by function normalizeCounts and then used as traditional expression matrix for PCA; we only used the top 2000 highly variable genes for PCA and the top 30 principal components for UMAP. All cell-type labels were included with the downloaded data.

**Table 1:** Datasets

Dataset	Species	Platform	Data access	Reference
Forebrain	Human	10x	*scvelo*	La Manno et al. [1]
Chromaffin	Mouse	SMART-seq2	*velocyto*	La Manno et al. [1], Furlan et al. [24]
FUCCI	Human	SMART-seq2	GSE146773	Mahdessian et al. [14]
Bonemarrow	Human	10x	*scvelo*	Setty et al. [19]
Dentategyrus LaManno	Mouse	10x	*scvelo*	La Manno et al. [1], Hochgerner et al. [20]
Pancreas	Mouse	10x	*scvelo*	Bergen et al. [3], Bastidas-Ponce et al. [21]
Gastrulation erythroid	Mouse	10x	*scvelo*	Pijuan-Sala et al. [22]
Dentategyrus Hochgerner	Mouse	10x	*scvelo*	Bergen et al. [3], Hochgerner et al. [20]
Gastrulation E7.5	Mouse	10x	***scvelo***	Pijuan-Sala et al. [22]
PBMC68k	Human	10x	*scvelo*	Zheng et al. [23]

For the following datasets, special treatments were applied. For the FUCCI dataset, for convenience, we scaled the FUCCI pseudotime to the range [0, 1] by dividing the pseudotime of each cell by the maximum pseudotime between cells. Cell cycle positions are estimated using the tricycle Bioconductor package [16]. For the Dentategyrus LaManno and PBMC68k datasets, instead of using the sum of spliced and unspliced across cells threshold 20, we used threshold 30 as the number of cells is considerable for these two datasets.

### Construction of the k-NN graph

The k-NN graph is constructed using the pp.neighbors function in the scVelo package with all default parameters. Internally, it runs functions from the Python umap package, which searches for *k* nearest neighbors in Euclidean distance of the top 30 PCs (default setting) and assigns weights to each edge [25] (for simulations with a number of genes less than 500, the scVelo package will use the spliced counts matrix instead of PCs). This results in an undirected weighted graph. We could use a symmetric $n \times n$ matrix $\textbf{W}$, of which column sums are normalized to 1, to represent such a k-NN graph. Given there is no guide on choosing the *k* in a new dataset, we have consistently used the $k=30$, the default in the scVelo package, unless otherwise specified.

### Smoothing of the count matrices

The smoothed count matrices Ms and Mu are calculated using the pp.moment function in the scVelo package with all default parameters. The smoothed count of a cell *i* for a particular gene *g* is given by the weighted sums of raw counts of k nearest neighbors:

$$\begin{aligned} Ms_{gi} = \sum _{j=1}^{n} (s_{gj} \cdot W_{ij}) \end{aligned}$$

$$\begin{aligned} Mu_{gi} = \sum _{j=1}^{n} (u_{gj} \cdot W_{ij}) \end{aligned}$$

Note that the spliced and unspliced counts are smoothed independently, but we use the same weighted k-NN graph for both.

### Gene-level velocity estimation

We use the scVelo tl.velocity function with mode = “deterministic” for steady-state model velocity estimation. For the dynamical model velocity estimation, we run the scVelo tl.recover_dynamics function to recover dynamics and then get the velocity values by the scVelo tl.velocity function with mode = “dynamical”. All parameters are defaulted as in the scVelo package. In brief, the steady-state model fits an extreme quantile regression line and calculates the residuals for each gene, which are returned as the gene-level velocity values. Along with the velocity matrix, we also get the estimated degradation rate $\gamma$ and the coefficient of determination for each gene. Only genes with a coefficient of determination greater than a pre-set threshold (0.01 used as default) will be labeled as velocity genes. Only these velocity genes will be used for visualization. Unlike the steady-state model, the dynamical model simultaneously solves the transcription rate $\alpha$, the splicing rate $\beta$, and the degradation rate $\gamma$. The inference is also made for each gene independently. For each gene, the direct output of the dynamical model is the gene parameters and a vector of latent time *t*, whose length is the number of cells *n*. The gene-level velocity value is then calculated using the system of Eqs. 3 and the 2. Note that we can only recover the parameters for a subset of genes, within which passes the pre-set threshold of coefficient of determination and likelihood are labeled as the velocity genes.

### Simulation settings

The simulation strategy is the same as described in Bergen et al. [6], as we used the simulation function in the scVelo package. Briefly, we simulate the spliced count and unspliced count matrices based on the system of Eq. 3. Note that the simulation process is independent for each gene, and it is trivial to get *s*(*t*) and *u*(*t*) as long as we have assigned other parameters. We use the transcription rate $\alpha = 5$, the splicing rate $\beta = 0.3$, and the degradation rate $\gamma = 0.5$ for all genes, as used in Bergen et al. [6]. To simulate data with a number of cells *n*, a cell-specific pseudotime vector is generated $\log$ uniformly distributed and scaled to the range $[0, t_{max}]$, with $t_{max}$ always set to 25 in our simulations. To make the counts different across genes, we further rescale the cell-specific time vector *t* to some interval within $[0, t_{max}]$. This step will keep the orders of cells but make all cells only cover some part of the full dynamics for a given gene. The same procedure is repeated for each gene to obtain a latent time matrix with *m* rows (*m* genes) and *n* columns (*n* cells). We can then plug the gene latent time matrix into the system of Eq. 3 to obtain the spliced count and unspliced count matrices. The true RNA velocity matrix comes naturally from the Eq. 2. After the generation of spliced and unspliced matrices, we add Gaussian noise with mean 0 and standard deviation $\sigma$ to the theoretical spliced and unspliced matrices to make the simulated data used in our analyses. The standard deviation $\sigma$ is equal to the noise level multiplied by the 99% percentile of the spliced or unspliced counts divided by 10 in our manuscript and in Bergen et al. [6]. Note that, as in Bergen et al. [6], we do not perform library size normalization and $\log _2$ transformation on the spliced count matrix. The k-NN graph construction and smoothing of counts matrices are performed as described previously. In the simulation where we use another “true” k-NN graph, the k-NN is calculated before we add Gaussian noise to the raw spliced and unspliced counts. Both the steady-state model RNA velocity and the dynamical model RNA velocity are inferred using the scVelo package, resulting in a velocity matrix *V* to be used later. Also, we force the labels of the “velocity gene” of all genes to be true, as in Bergen et al. [6]. For the steady-state model, the estimated velocity of all *m* genes will be used for later visualization. For the dynamical model, some of the genes are still excluded from visualization due to missing velocity estimations from the dynamical model. Coercion could potentially improve visualization results since we know that all genes are true “velocity genes.”

### Mapping high-dimensional velocities into a low-dimensional embedding

After getting the velocity matrix, which contains a velocity value for $m^*$ ($m^*$ is the “velocity gene” that passes predefined thresholds) genes and *n* cells, we need to map the high-dimensional velocity into the same low-dimensional embedding of expression (spliced counts). In our manuscript, we use the following three methods to map high-dimensional velocities, with the first two used by La Manno et al. [1]. We use $\textbf{S}$ to represent the expression matrix (raw spliced counts matrix), which has the shape of m rows (genes) and n columns (cells). Note that we describe the precise procedures here, which might look slightly different from the simplified version in the “Results” section.

#### Direct projection in PCA

We have used this method only on simulated data with known true velocity. Theoretically, this method could be used in any embedding methods with a linear operator $f: \mathbb {R}^{m \times n} \rightarrow \mathbb {R}^{n \times p}$. Specifically for PCA, we have

$$\begin{aligned} \textbf{P} = \tilde{\textbf{S}} ^ t \cdot \textbf{R} \end{aligned}$$

where $\textbf{R}$ represents the m-by-p rotation matrix ; $\tilde{\textbf{S}}$ is a m-by-n spliced count matrix with row-means centered. The resulting n-by-p $\textbf{P}$ is the cell-level principal components matrix. We can map velocities as

$$\begin{aligned} \textbf{V}_{\textbf{p}} = \textbf{V} ^ t \cdot \textbf{R} \end{aligned}$$

The formulation here is much simpler than the application to real data, as we omit library size normalization, $\log _2$ transformation, and highly variable gene selection. Note that in the simple simulation setting and PCA space, we project the velocity vectors directly, which is (almost) equivalent to taking the difference between the future state ($\textbf{S}^{*}$ denotes the future expression state matrix) and current state:

$$\begin{aligned} f(\textbf{S}^{*}) - f(\textbf{S})&= f(\textbf{S} + \textbf{V} \cdot \Delta t) - f(\textbf{S})\\&= f(\textbf{S}) + f(\textbf{V} \cdot \Delta t) - f(\textbf{S})\\&= f(\textbf{V}) \cdot \Delta t \end{aligned}$$

$f(\textbf{S}^{*}) - f(\textbf{S}) = f(\textbf{V})$ when $\Delta t = 1$. It is clear that $\Delta t$ is a trivial scalar since all vectors are rescaled in the final visualization. However, the choice of $\Delta t$ would matter in real data as non-linear operations are involved, such as normalization of the library size and transformation of $\log _2$.

#### Velocity transition probability method

The velocity transition probability method was first introduced by La Manno et al. [1] and was reused by Bergen et al. [3] with some modifications. We use the implementation by Bergen et al. [3] with all default parameters in the scVelo package.

For a given cell *i*, we have its velocity vector $\vec {v}_i$ and its expression vector $\vec {Ms}_{i}$ (the length for both two is $m^*$ since we only consider the “velocity” genes filtered by scVelo). Also, note that the Ms matrix is being used for the transition probability instead of the raw spliced count matrix. The first step to get the velocity transition probability matrix is the calculation of an n-by-n cosine similarity matrix. The advantage of using cosine similarity or PCC to quantify the relationship between cells is that we do not need to choose $\Delta t$. For a cell *i*, we consider cells that are cell *i*’s k-nearest neighbors and recursive k-nearest neighbors, denoted as $\{n(n(i, k), k)\}$. The k-NN graph is precalculated on the PCA of the raw spliced count matrix and was used for Ms and Mu. The cosine similarity between cell *i* and cell *j* is given as

$$\begin{aligned} \pi (i, j) = \left\{ \begin{array}{ll} \frac{(\vec {Ms}_i - \vec {Ms}_j) \cdot \vec {v}_i}{\Vert \vec {Ms}_i - \vec {Ms}_j \Vert \cdot \Vert \vec {v}_i\Vert } &{} \quad \text {if}\ j \in \{n(n(i, k), k)\} \setminus \{i\}\\ 0 &{} \quad \text {if}\ j \not \in \{n(n(i, k), k)\} \setminus \{i\} \end{array}\right. \end{aligned}$$

The exponential kernel is then applied to the cosine similarity matrix to get the velocity transition matrix. Specifically, the transition probability from cell *i* to cell *j* is given as

$$\begin{aligned} \tilde{\pi }(i \rightarrow j) = \frac{1}{z_i} exp(\frac{\pi (i, j)}{\lambda }) \end{aligned}$$

with $z_i$ as the cell normalization factor $z_i = \sum _j exp(\frac{\pi (i, j)}{\lambda })$ and the constant kernel width parameter $\lambda$. There are optional variance stabilization transformations mentioned in La Manno et al. [1] and Bergen et al. [3], but we use the default parameter in scVelo, which does not perform any variance stabilization transformations. Given a embedding $\textbf{Q}$, the normalized location difference between cell *i* and *j* is $\vec {d}(i, j) = \frac{\vec {Q}_j - \vec {Q}_i}{\Vert \vec {Q}_j - \vec {Q}_i \Vert }$. The mapped low-dimensional vector for cell *i* is calculated as

$$\begin{aligned} f^*(\vec {v}_i) = \sum _{j \not = i} [(\tilde{\pi }(i \rightarrow j) - \frac{1}{n}) ~ \vec {d}(i, j)] \end{aligned}$$

where $f^*$ is a non-existing symbolic function. The idea behind the velocity transition probability is to weigh the likelihood that the cell *i* will become the cell *j* in the future state.

#### UMAP transform method

The UMAP transform method is an experimental method that has not been used in previous papers on RNA velocity [1, 3]. Unfortunately, we were unable to systematically confirm the correctness of the method since the UMAP transform function itself has not been systematically evaluated. To illustrate, we use *f* to represent the function that projects data into the PCA space. Since we use the PCA results as the input of the UMAP function, the UMAP embedding is given as $g(f(\textbf{S})) = \textbf{Q}$. Note that we use the function $g: \mathbb {R}^{n \times p} \rightarrow \mathbb {R}^{n \times 2}$ to represent the UMAP embedding process, but *g* is not as straightforward as the function *f*. Before we transform new points into the existing UMAP space, we need to get new PCA coordinates, which requires us to choose a $\Delta t$. The coordinates of the future states in PCA are given by $f(\textbf{S}^{*}) = f(\textbf{S} + \textbf{V} \cdot \Delta t) = f(\textbf{S}) + f(\textbf{V} \cdot \Delta t)$. Thus, the coordinates of future states in UMAP are given as

$$\begin{aligned} g(f(\textbf{S}^{*})) = g(f(\textbf{S}) + f(\textbf{V} \cdot \Delta t)) \end{aligned}$$

Note that *g* is not a linear function, so we could not expand the right part. Finally, we map the high-dimensional velocities into the low-dimensional UMAP space as

$$\begin{aligned} g(f(\textbf{S}^{*})) - g(f(\textbf{S})) = g(f(\textbf{S} + \textbf{V} \cdot \Delta t)) - g(f(\textbf{S})) \end{aligned}$$

In our analysis, we always use $\Delta t = 1$. We admit that this is a somewhat random choice, but it is hard to argue the choice because the fuzzy definition and poor interpretability of pseudotime/latent time *t* inherits from the RNA velocity models.

### Vector field visualization

After getting the low-dimensional cell-level vector field, we need to process it further as we could not show that many vectors (arrows) due to overplotting. We use two visualization strategies for the final visualization: the gridding method and streamline plot. While the streamline plot is aesthetically more appealing, we turn to the gridding method whenever we want to highlight more details. Again, as we have mentioned previously, the same vector field may look quite different when comparing the two methods. For all vector fields in the manuscript, we adapt the streamline plot and the gridding method implemented in the velociraptor R package [26] (Additional file 1: Fig. S19 is an exception). The streamline plot connects vectors flowing towards and in a similar direction. For the streamline plot, which is used for real datasets, we use a resolution between 13 and 20. The gridding method takes the average of all vectors in the grid box. The averaged vectors are further scaled to look good based on the axis range. For the gridding method, we use a resolution of 20 for simulated data and 30 for the pancreas data. We note that there is no existing metric to guide us in choosing the best resolution, so we have to choose a resolution that, we think, makes sense based on the number of data points and the embedding structure to balance the details and overplotting.

### Calculation of the speed

The speed (vector length) in both high-dimensional space and low-dimensional space is defined as the $\ell$-2 norm of the vector of each cell. Specifically, for high-dimensional velocities, the speed of cell *i* is $L_i=\sqrt{\sum _{g \in \{\text {velocity genes}\}} v_{gi}^2}$ with $v_{gi}$ the velocity estimation of gene *g*. For low-dimensional embedding, the speed of cell *i* is $L_i=\sqrt{ o_{1i}^2 + o_{2i}^2}$ with $o_{1i}$ the mapped vector in the first dimension and $o_{2i}$ the mapped vector in the second dimension.

### Calculation of Pearson’s correlation coefficient (PCC)

For two continuous variables, *X* and *Y*, the formula for Pearson’s correlation coefficient is given by:

$$\begin{aligned} r = \frac{\sum _{i=1}^{n}(x_i - \bar{x})(y_i - \bar{y})}{\sqrt{\sum _{i=1}^{n}(x_i - \bar{x})^2 \sum _{i=1}^{n}(y_i - \bar{y})^2}} \end{aligned}$$

Here, $\bar{x}$ and $\bar{y}$ represent the mean values of variables *X* and *Y*, respectively, and *n* is the total number of paired observations.

### Comparison of directions of the cell-level vectors

In Fig. 2d, we analyze the cell-level vector directions of 53 pre-endocrine cells, as identified within the annotated rectangle in Fig. 2b and c. After mapping the high-dimensional RNA velocity data onto the UMAP embeddings, we compute the angles for each cell-level vector. To estimate the kernel density of the two circular variables, we employ the von Mises Kernel, which is a circular normal kernel. We then use Watson’s two-sample test of homogeneity from the R circular package to compare the two circular variables.

### Calculation of explained variation $R^2$ of the fitted loess model

The coefficient of determination $R^2$ for the fitted loess model is calculated using the following equation:

$$\begin{aligned} R^2 = 1 - \frac{SS_{\text {res}}}{SS_{\text {total}}} \end{aligned}$$

Here, $SS_{\text {res}} = \sum {i}^{n} (y_i - \hat{y_i})^2$ and $SS_{\text {total}} = \sum {i}^{n} (y_i - \bar{y})^2$. For the FUCCI pseudotime or tricycle cell cycle position, since they track a full cell cycle, we fit a periodic loess model $y \sim t$, where *y* is any response variable (e.g., Ms or Mu), by concatenating triple *y* and triple FUCCI pseudotime *t* with one period shift to form [*y*, *y*, *y*] and $[t - 1, t, t + 1]$ (or $[t - 2 \pi , t, t + 2 \pi ]$ for cell cycle position). Note that we only use the original data points (the middle copy) for calculating $SS_{\text {res}}$ and $SS_{\text {total}}$, rather than all three copies of data points.

For the velocity latent time, we cannot determine whether the latent time spans a complete period. Therefore, we fit a standard loess model instead of the periodic model described above for the velocity latent time.

### Calculation of normalized root mean square error (NRMSE)

In the simulations, we know the true values, such as the true RNA velocity at the gene level. We use both PCC and NRMSE to quantify how good the estimations are. The NRMSE is calculated as

$$\begin{aligned} NRMSE = \sqrt{\frac{\sum (y_i - \hat{y_i})^2}{n \sigma ^2 }} \end{aligned}$$

Here, $y_i$ represents the true values; $\hat{y_i}$ are the estimated values; *n* is the number of observations. The standard deviation of the true values $\sigma$ is used to normalize the root mean square error for making different simulations comparable.

### Supplementary Information

**Additional file 1:** Contains Figs. S1-S20 and supplementary notes on “The differences between implementations of RNA velocity analysis” and “Visualization of 2D vector fields”.

**Additional file 2.** Contains the review history.

## Data Availability

All data sets we use are list in the Table 1. The Forebrain data [1], the Bonemarrow data [19], the Dentategyrus LaManno data [1, 20], the Pancreas data [3, 21], the Gastrulation erythroid data [22], the Dentategyrus Hochgerner data [3, 20], the Gastrulation E7.5 data [22], and the PBMC68k data [23] are all available from the scVelo package directly at https://scvelo.readthedocs.io/api/#datasets [3]. The Chromaffin data [1, 24] is available at http://pklab.med.harvard.edu/velocyto/notebooks/R/chromaffin2.nb.html. The FUCCI data [14] is available at https://drive.google.com/file/d/149ICTtieYjuKWZoLwRLzimwff0n6eWqw/view?usp=sharing. The code for data analysis and figure generation associated with this work is available under the MIT License. It can be accessed on our GitHub repository at https://github.com/hansenlab/RNAVelocityCode [27] and has also been archived on Zenodo with the DOI 10.5281/zenodo.8299826 [28].
