# Supplementary material for: Pumping the brakes on RNA velocity by understanding and interpreting RNA velocity estimates
Source: Genome Biol. 2023 Oct 26;24:246. doi: 10.1186/s13059-023-03065-x (PMC10601342; doi:10.1186/s13059-023-03065-x)
Supplement: Supplementary file 1 — Additional file 1: Contains Figs. S1-S20 and supplementary notes on “The differences between implementations of RNA velocity analysis” and “Visualization of 2D vector fields”. [file 13059_2023_3065_MOESM1_ESM.pdf]

ADDITIONAL FILE 1

Pumping the brakes on RNA velocity by understanding and interpreting RNA velocity estimates

Shijie C. Zheng, Genevieve Stein-O'Brien, Leandros Boukas,
Loyal A. Goff*, and Kasper D. Hansen*

* Correspondence to loyalgoff@jhmi.edu (LAG), khansen@jhsph.edu (KDH)

CONTENTS

1 Supplementary Figures **2**

2 Supplementary Notes **23**

2.1 The differences between implementations of RNA velocity analysis 23

2.2 Visualization of 2d vector fields 25

1 SUPPLEMENTARY FIGURES

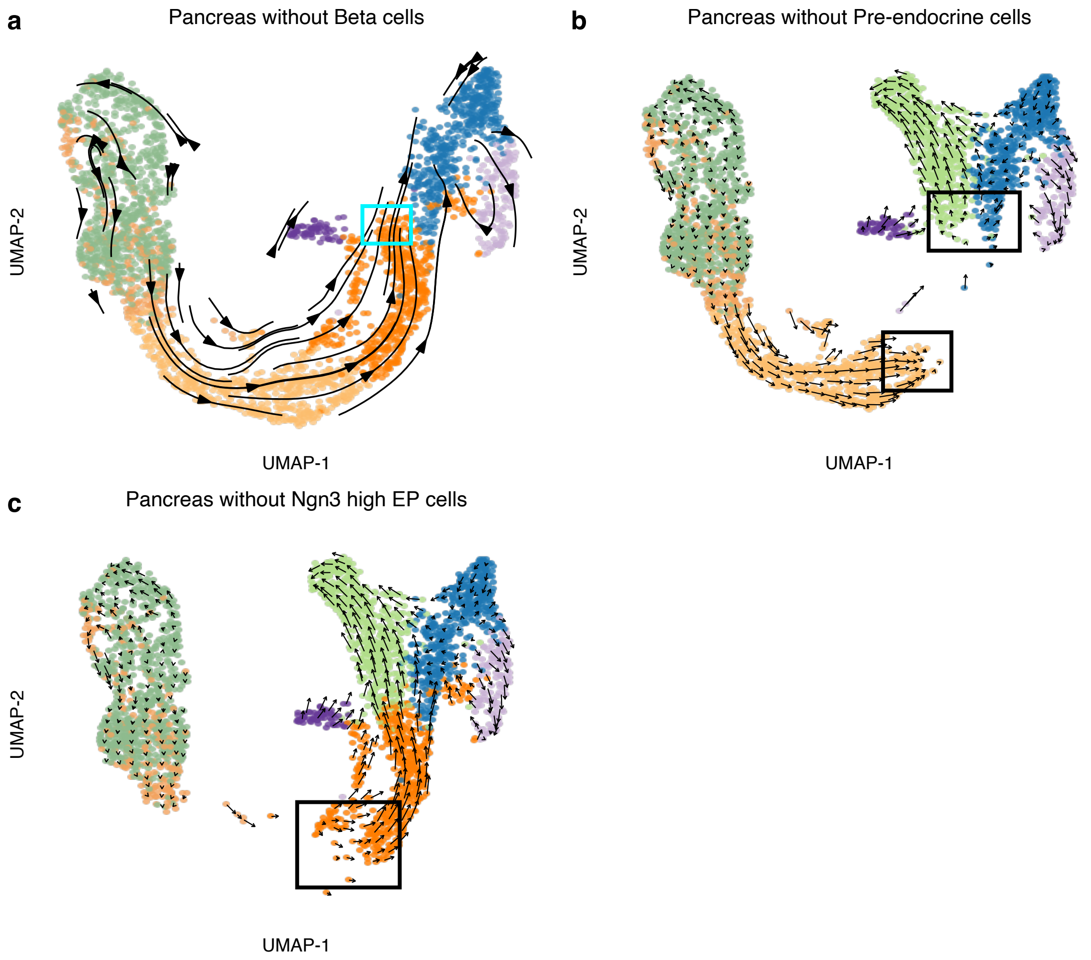

Fig. S1. Mapped RNA velocity in the pancreas dataset after removing a cell type. (a) Dynamical model based RNA velocity is visualized on the UMAP for the pancreas data after removing the beta cells. **(b)** As (a), but the pre-endocrine cells are removed instead. **(c)** As (a), but the Ngn3 high EP cells are removed instead. Note that the arrows disappear for cells in the black rectangles of (b) and (c).

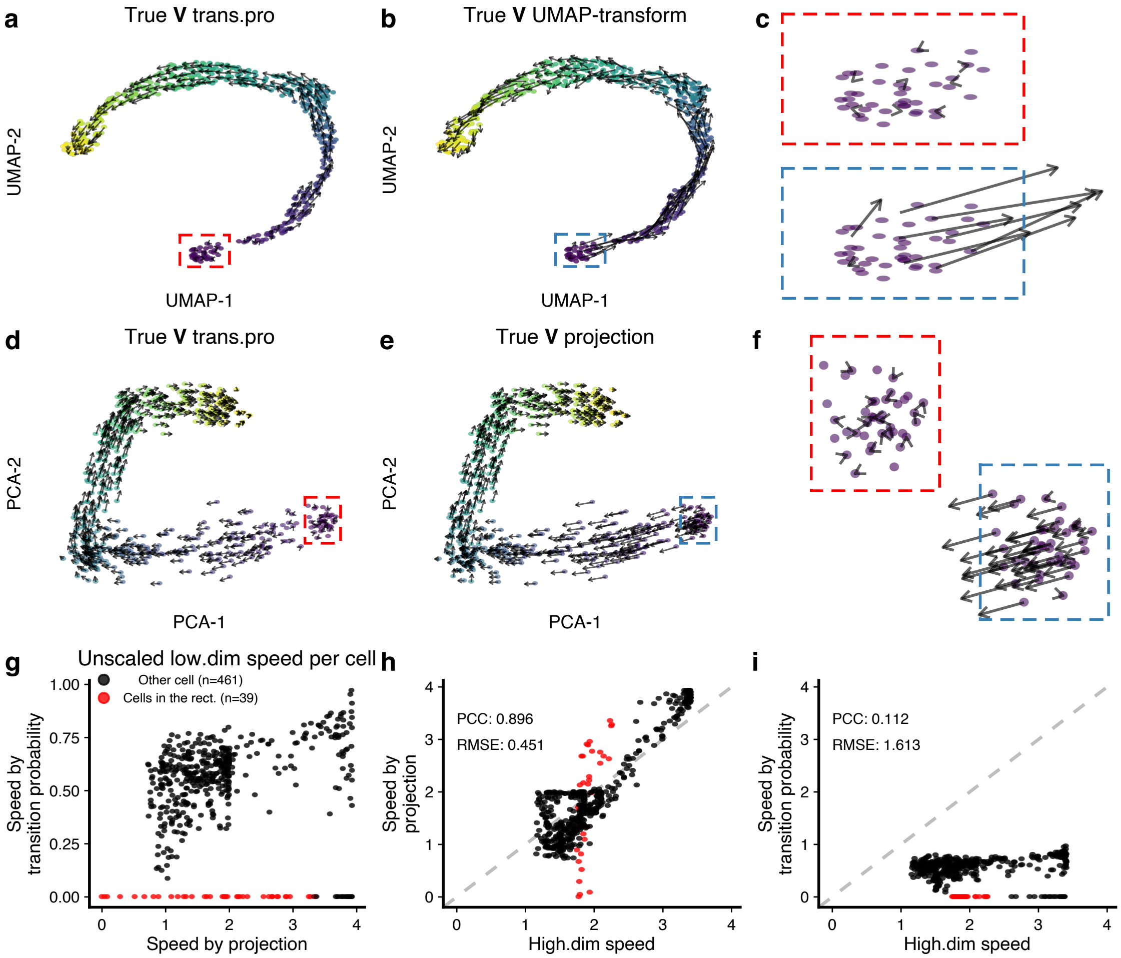

Fig. S2. Missing mapped cell-level vectors at the start of the trajectory by the transition probability method. (a) True RNA velocity vector field on UMAP using the transition probability (trans.pro) method. **(b)** As (a), but the high-dimensional (high.dim) velocities are mapped by the UMAP-transform method. **(c)** Zoom-ins of the red and blue rectangle labeled regions in (a) and (b). **(d)** True RNA velocity vector field on PCA using the transition probability method. **(e)** As (d), but the high-dim velocities are mapped by the projection method. **(f)** Zoom-ins of the red and blue rectangle labeled regions in (d) and (e). **(g)** Comparison of the low-dimensional (low.dim) speed produced by two methods in (d) and (e). **(h)** Comparison between the low-dimensional speed produced by projection and the high-dimensional speed. **(i)** Comparison between the low-dimensional speed produced by transition probability and the high-dimensional speed. (Abbreviations: trans.pro: transition probability; high.dim: high-dimensional; low.dim: low-dimensional.)

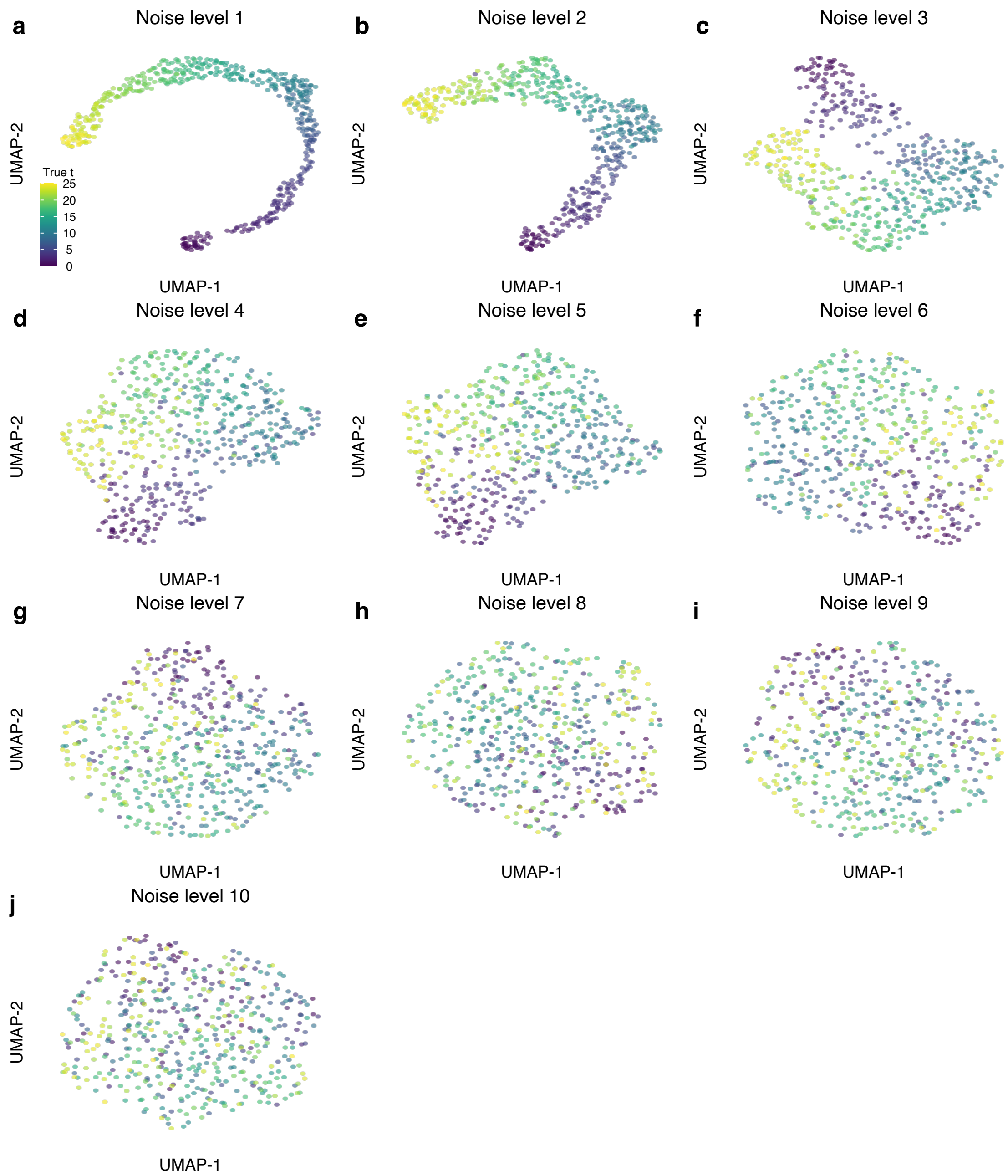

Fig. S3. UMAP representation of the simulated data with different noise levels. Each point represents a cell, colored by the true latent time. We use a different noise level in each panel, increasing from 1 to 10. Similar to the PCA representation, the UMAP representation becomes like a big blob more and more with the noise level increases.

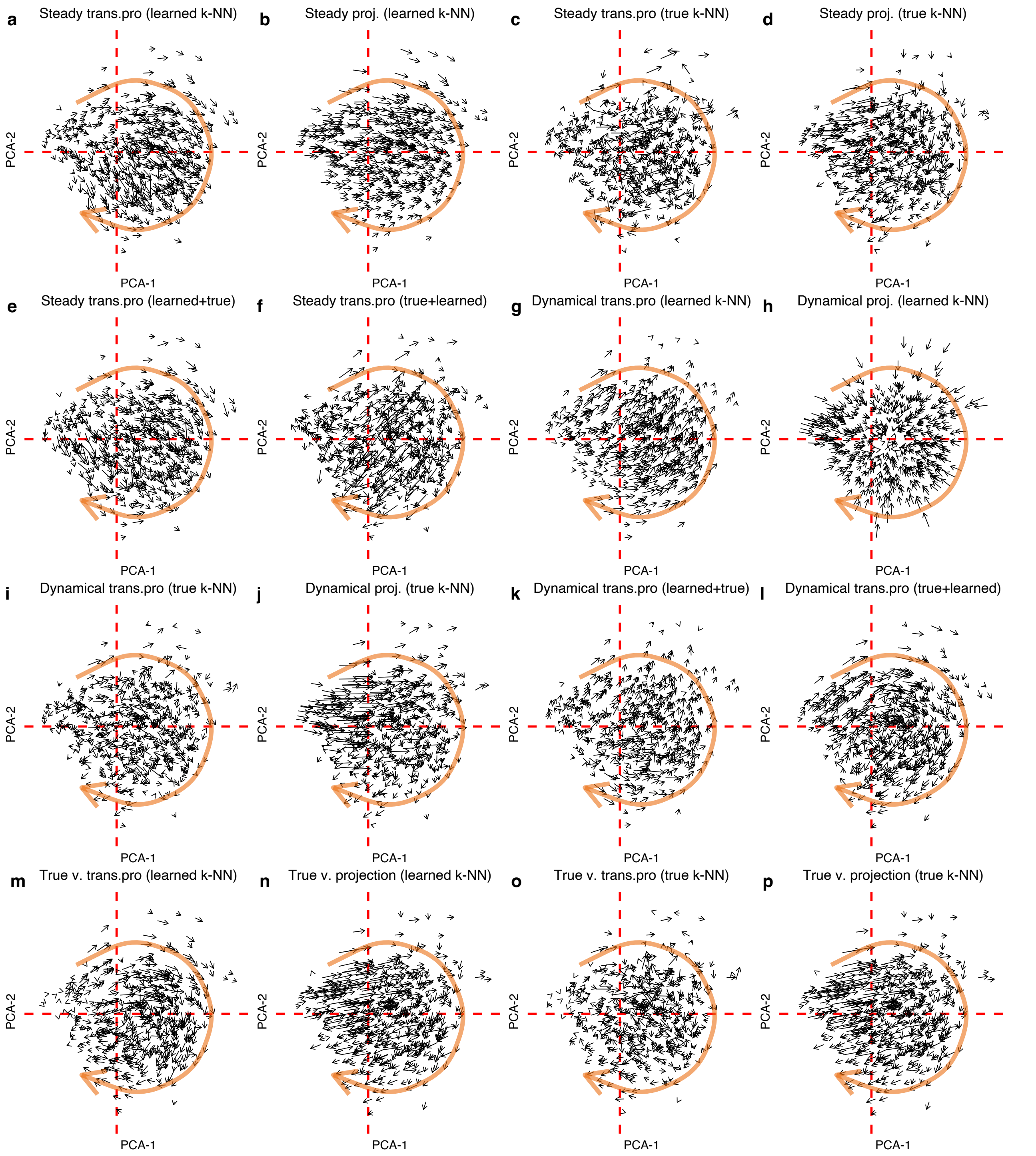

Fig. S4. Visualized RNA velocity vector field using simulated data at noise level 5. See the next page for captions.

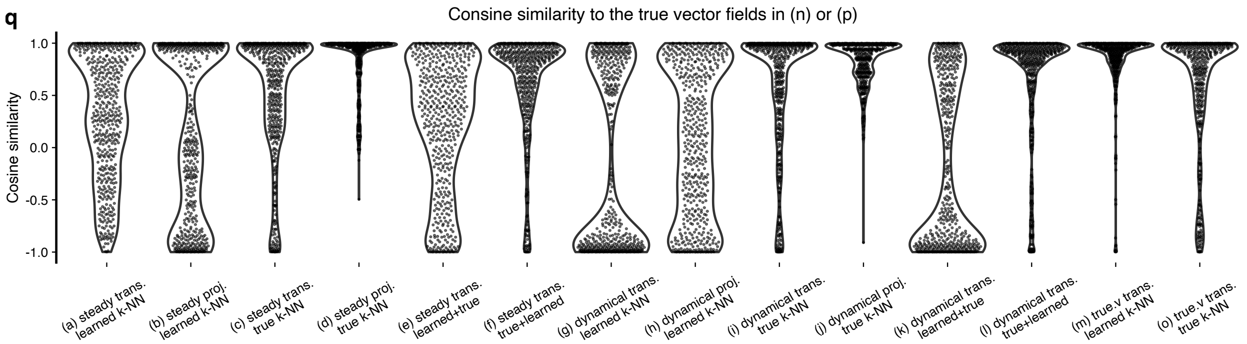

Fig. S4. (Continued) (a-p) Visualized vector fields in all combinations of the following: types of high-dimensional velocities (estimated high-dimensional velocities from the steady-state model or estimated high-dimensional velocities from the dynamical model, or true high-dimensional velocities), mapping methods (transition probability or direct PCA projection), types of k-NN used for preprocessing (learned k-NN or true k-NN), and types of k-NN used for calculation of transition probability (learned k-NN or true k-NN). In (a-p), the type of velocities is given first in the panel title and followed by the mapping methods (trans.pro for transition probability and proj. for direct PCA projection). The type of k-NN is given in the parenthesis of each panel title. If there is one type of k-NN, then that type of k-NN is used for smoothing and transition probability calculation. If two types of k-NN are given, the first k-NN is used for smoothing, and the second is for transition probability calculation. In all panels, each point represents a cell. The big orange arrow approximates the true direction of the trajectory. Note that (n) and (p) is the same because the true velocity is unrelated to smoothing. **(q)** The cosine similarities between the mapped cell-level vectors and the "true" mapped cell-level vectors in (n) or (p). (Abbreviations: trans.pro: transition probability; proj.: projection; trans.: transition probability; v.: velocity.)

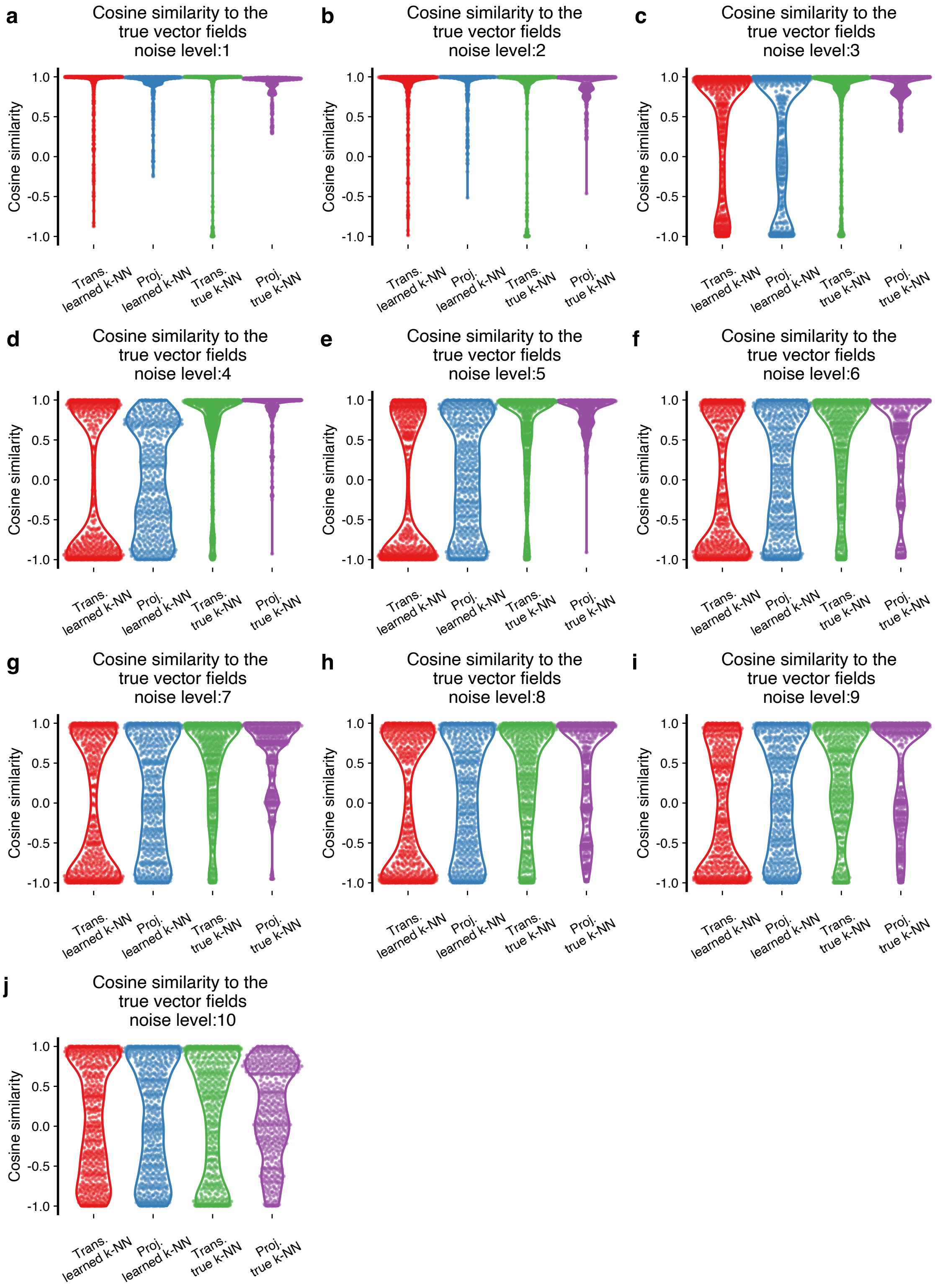

Fig. S5. Cosine similarities between the mapped cell-level vectors and the "true" mapped cell-level vectors at various noise levels. An extension of Fig. 5 with additional noise levels. (Abbreviations: trans.: transition probability; proj.: projection.)

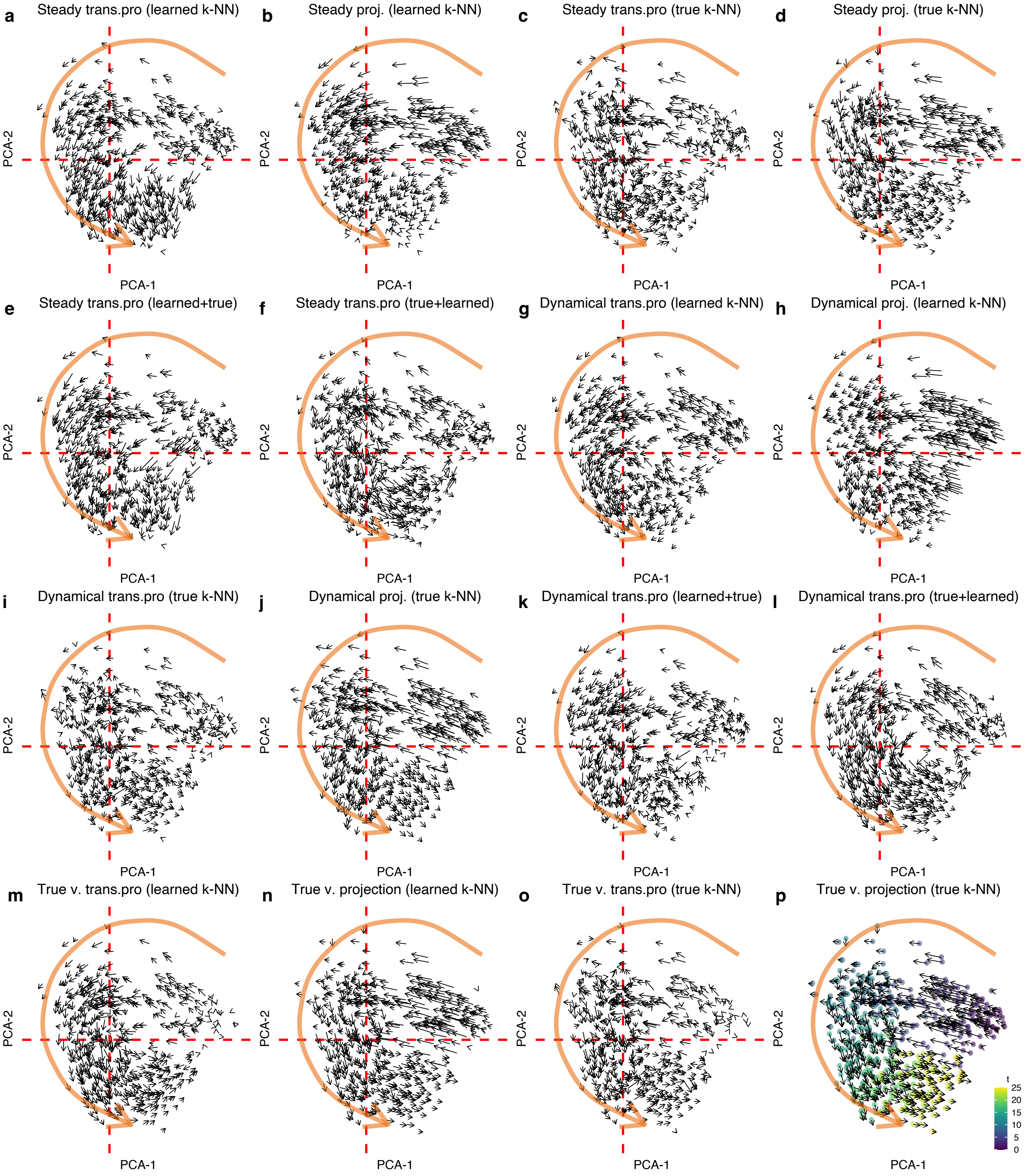

Fig. S6. Visualized RNA velocity vector fields using simulated data at noise level 3. Similar to Fig. S4, but the noise level is lowered to 3. See the next page for captions.

q

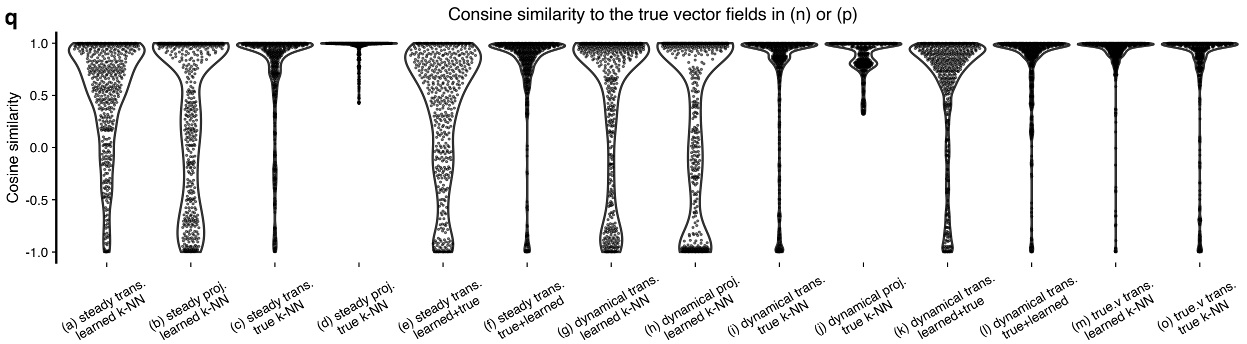

Fig. S6. (Continued) (a-p) Visualized vector fields in all combinations of the following: types of high-dimensional velocities (estimated high-dimensional velocities from the steady-state model or estimated high-dimensional velocities from the dynamical model, or true high-dimensional velocities), mapping methods (transition probability or direct PCA projection), types of k-NN used for preprocessing (learned k-NN or true k-NN), and types of k-NN used for calculation of transition probability (learned k-NN or true k-NN). In (a-p), the type of velocities is given first in the panel title and followed by the mapping methods (trans.pro for transition probability and proj. for direct PCA projection). The type of k-NN is given in the parenthesis of each panel title. If there is one type of k-NN, then that type of k-NN is used for smoothing and transition probability calculation. If two types of k-NN are given, the first k-NN is used for smoothing and the second for transition probability calculation. In all panels, each point represents a cell. The big orange arrow approximates the true direction of the trajectory. Note that (n) and (p) is the same because the true velocity is unrelated to smoothing. **(q)** The cosine similarities between the mapped cell-level vectors and the "true" mapped cell-level vectors in (n) or (p). (Abbreviations: trans.pro: transition probability; proj.: projection; trans.: transition probability; v.: velocity.)

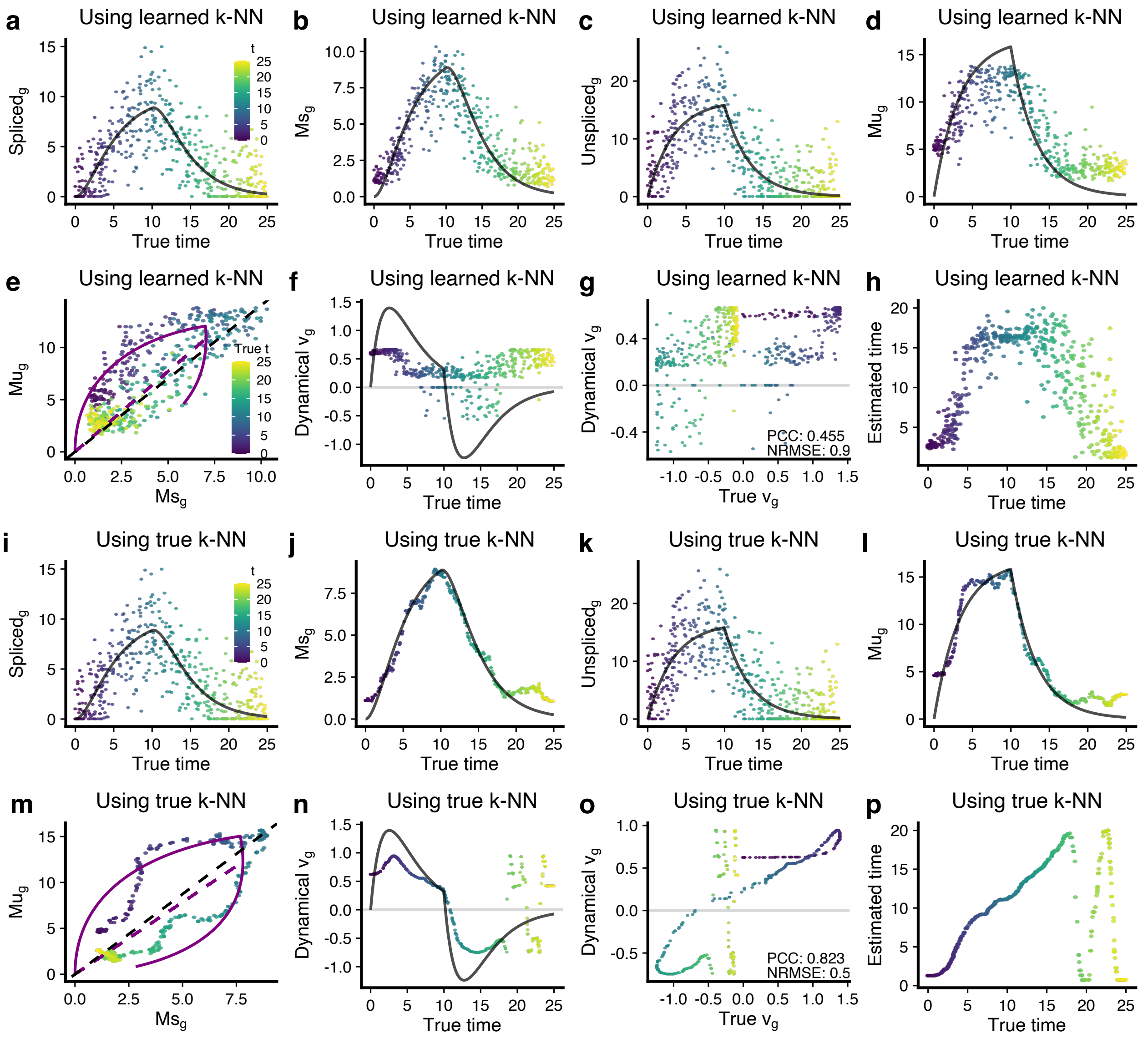

Fig. S7. Gene-level RNA velocity estimation analyses using simulated data at noise level 3. This figure complements Fig. 6. In all panels, a data point represents a cell and is colored by the known true latent time t. All solid black lines represent the known true values. **(a-d)** Scatter plots show the spliced counts, Ms, unspliced counts, and Mu over the true latent time t for a random gene in simulation. **(e)** Phase portrait shows the Ms over Mu for the same gene. The parameters are estimated by the dynamical model. **(f)** Estimated velocity (points) and true velocity (black line) over true latent time t. **(g)** Scatter plot compares the estimated velocity values to the true velocity values. PCC and NRMSE are given. **(h)** Scatter plot compares the estimated latent time to the true latent time. **(i-p)** As (a-h), but now we use the true k-NN to get Ms and Mu matrices. The estimated velocity values are much closer to the true velocity values with PCC 0.823 and NRMSE 0.584. Note that (a) and (i) are identical, and (c) and (k) are identical, too, since the k-NN does not affect raw counts. (Abbreviations: PCC: Pearson's correlation coefficient; NRMSE: Normalized Root Mean Square Error.)

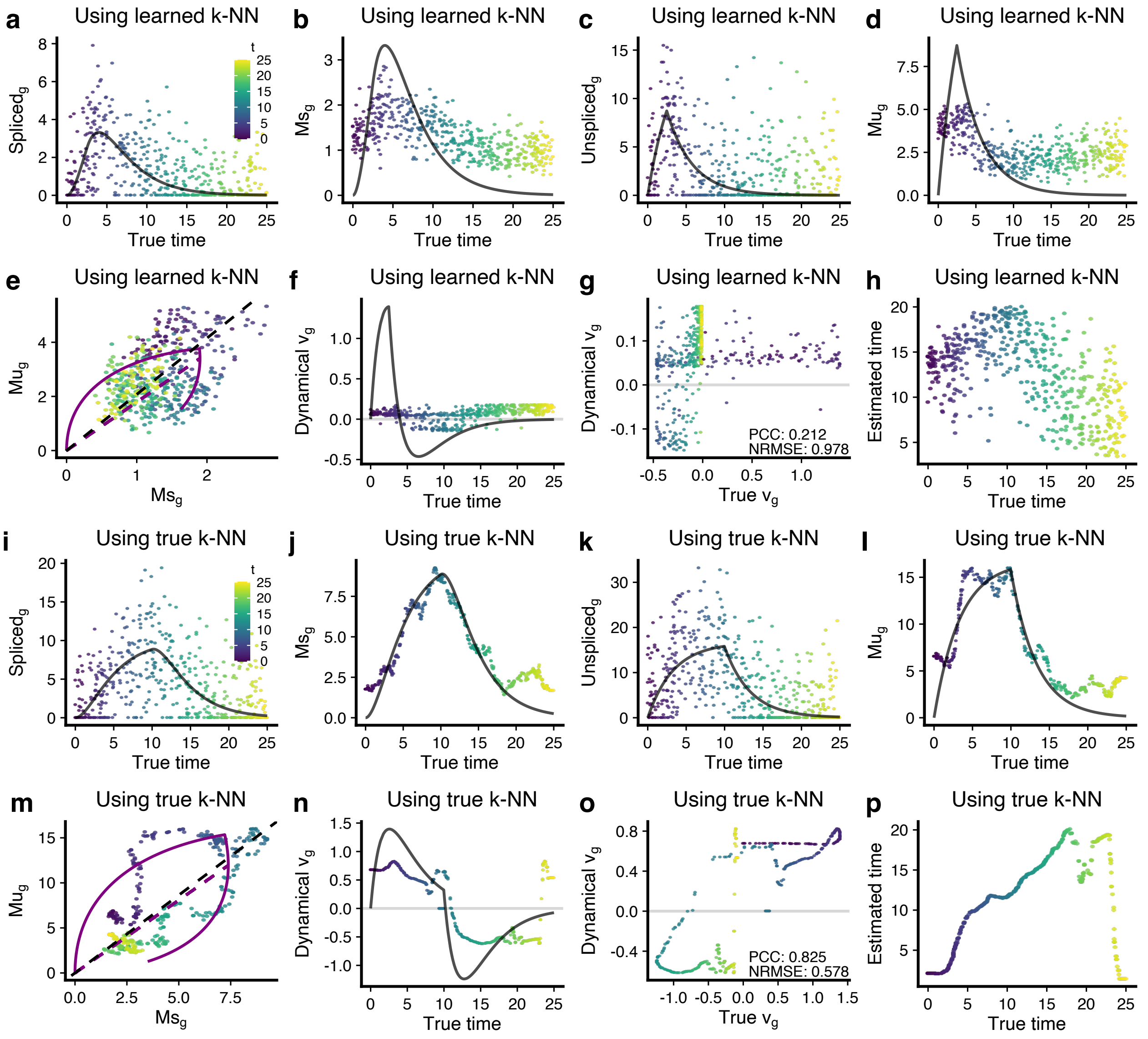

Fig. S8. Gene-level RNA velocity estimation analyses using simulated data at noise level 5 This figure is similar to Fig. S7, but the noise level is 5. In all panels, a data point represents a cell and is colored by the known true latent time t. All solid black lines represent the known true values. **(a-d)** Scatter plots show the spliced counts, Ms, unspliced counts, and Mu over the true latent time t for a random gene in simulation. **(e)** Phase portrait shows the Ms over Mu for the same gene. The parameters are estimated by the dynamical model. **(f)** Estimated velocity (points) and true velocity (black line) over true latent time t. **(g)** Scatter plot compares the estimated velocity values to the true velocity values. PCC and Normalized Root Mean Square Error (NRMSE) are given. **(h)** Scatter plot compares the estimated latent time to the true latent time. **(i-p)** As (a-h), but now we use the true k-NN to get Ms and Mu matrices. The estimated velocity values are much closer to the true velocity values with PCC 0.825 and NRMSE 0.578. (Abbreviations: PCC: Pearson's correlation coefficient; NRMSE: Normalized Root Mean Square Error.)

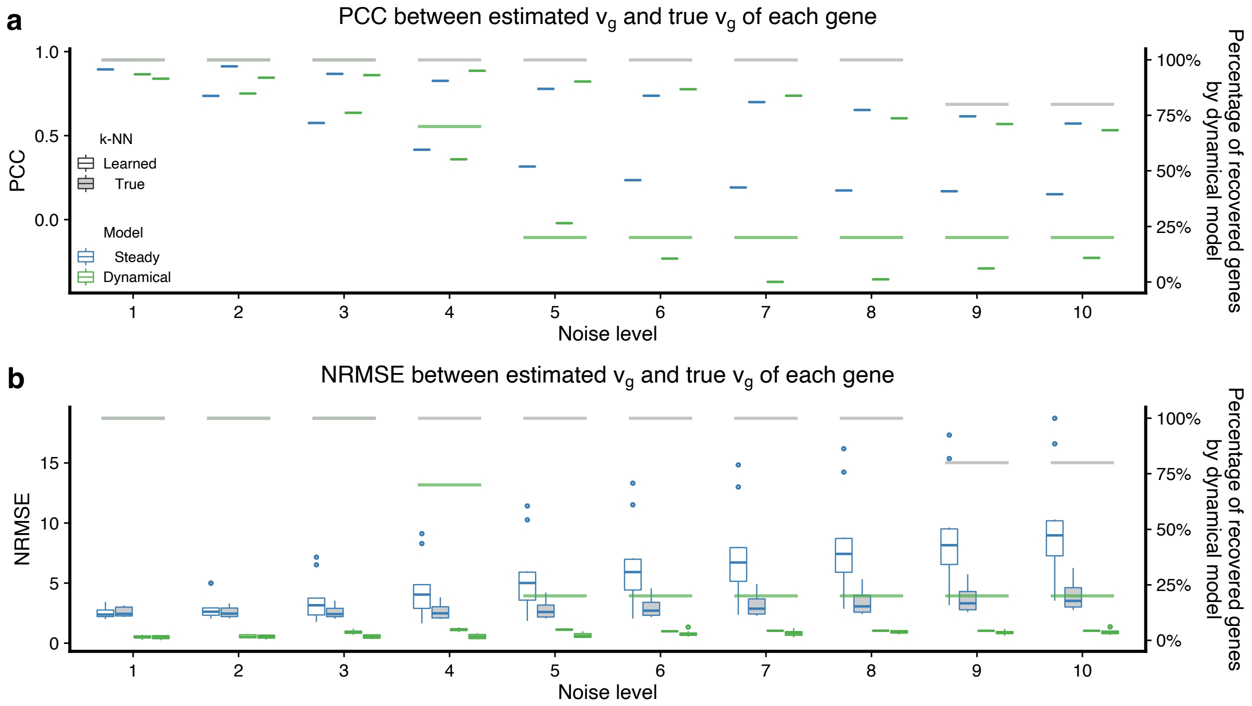

Fig. S9. Comprehensive gene-by-gene evaluations of high-dimensional velocity estimations using simulations. (a) Boxplots show PCC between the estimated velocities and the true velocities of each gene. **(b)** Boxplots show NRMSE between the estimated velocities and the true velocities of each gene. All boxes show the left y-axis values and indicate the 25th and 75th percentiles. Whiskers extend to the largest values no further than $1.5 \times$ interquartile range (IQR) from these percentiles. The grey and green horizontal lines correspond to the percentage of recovered genes by the dynamical model using the true and learned k-NN graph, respectively. (Abbreviation: IQR: interquartile range; PCC: Pearson's correlation coefficient; NRMSE: Normalized Root Mean Square Error.)

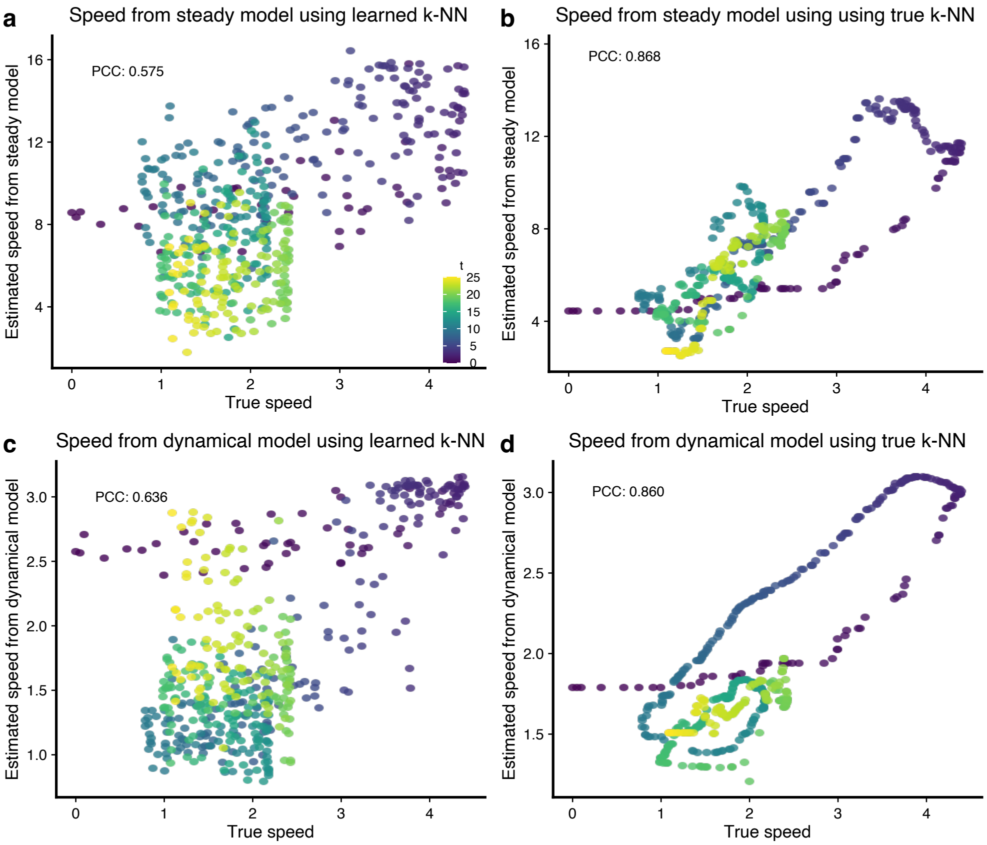

Fig. S10. Comparisons of high dimensional speed at noise level 3. (a) Scatter plot compares the speed of true (high-dimensional) velocity to that of the estimated (high-dimensional) velocity by the steady-state model using the learned k-NN graph. **(b)** As (a), but we use the true k-NN to infer (high-dimensional) velocity. **(c-d)** As (a-b), but the velocities are estimated using the dynamical model. (Abbreviation: PCC: Pearson's correlation coefficient.)

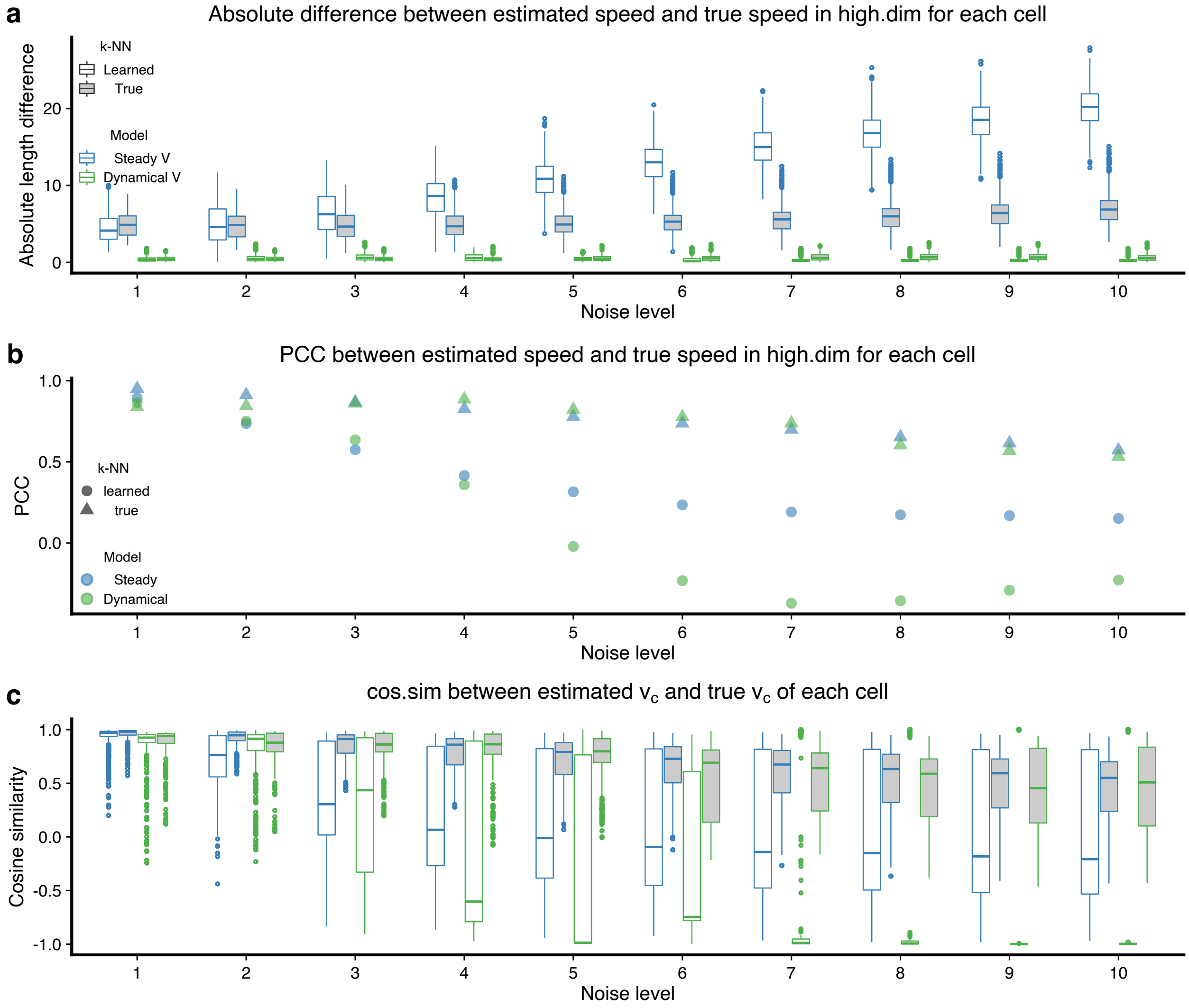

Fig. S11. Comprehensive cell-by-cell evaluations of high-dimensional velocity estimations using simulations. (a) Boxplots show the absolute difference between the estimated speed and the true speed of each cell. **(b)** We show the PCC between the cell-level estimated high-dimensional speed and the high-dimensional true speed. **(c)** Boxplots show cosine similarity between the estimated velocities and the true velocities of each cell. Whiskers extend to the largest values no further than 1.5 × interquartile range (IQR) from these percentiles. (Abbreviations: PCC: Pearson's correlation coefficient; IQR: interquartile range; est. v.: estimated velocity; true v.: true velocity; high.dim: high-dimensional; cos.sim: cosine similarity.)

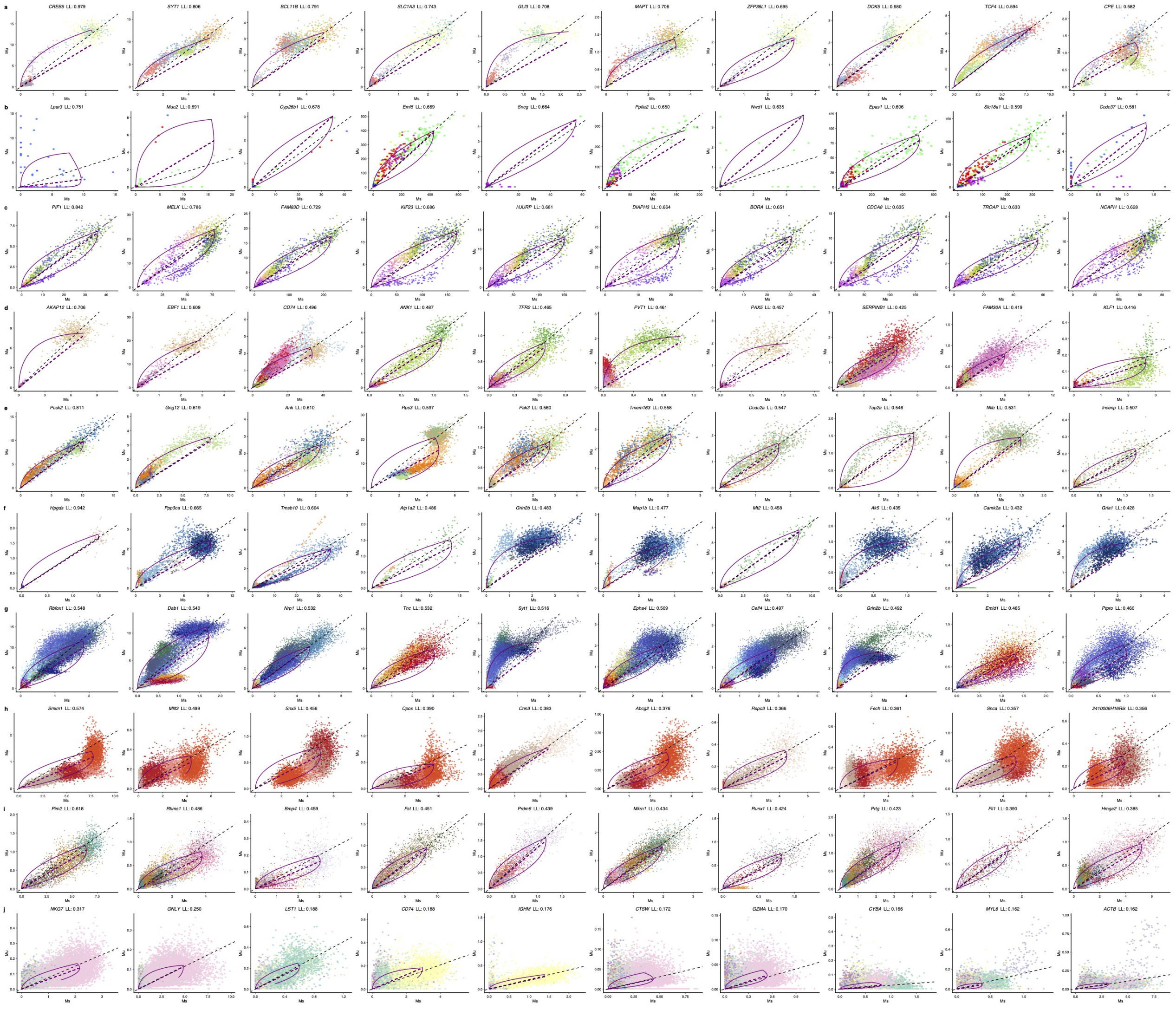

Fig. S12. The phase portrait of top 10 genes in the 10 real datasets. Each point represents a cell, colored by cell type or FUCCI pseudotime. Form (a-j), the order of the datasets is listed as in Table 1: Forebrain, Chromaffin, FUCCI, Bonemarrow, Dentategyrus Lamanno, Pancreas, Gastrulation erythroid, Dentategyrus Hochgerner, Gastrulation E7.5, and PBMC68k. The top 10 genes ranked by the likelihood from the dynamical model are shown from left to right for each dataset. Purple lines represent the fitted dynamics by the dynamical model, and the black dashed line represents the degradation rate (slope) from the steady-state model. Note that most genes do not show complete up- and down- regulation dynamics.

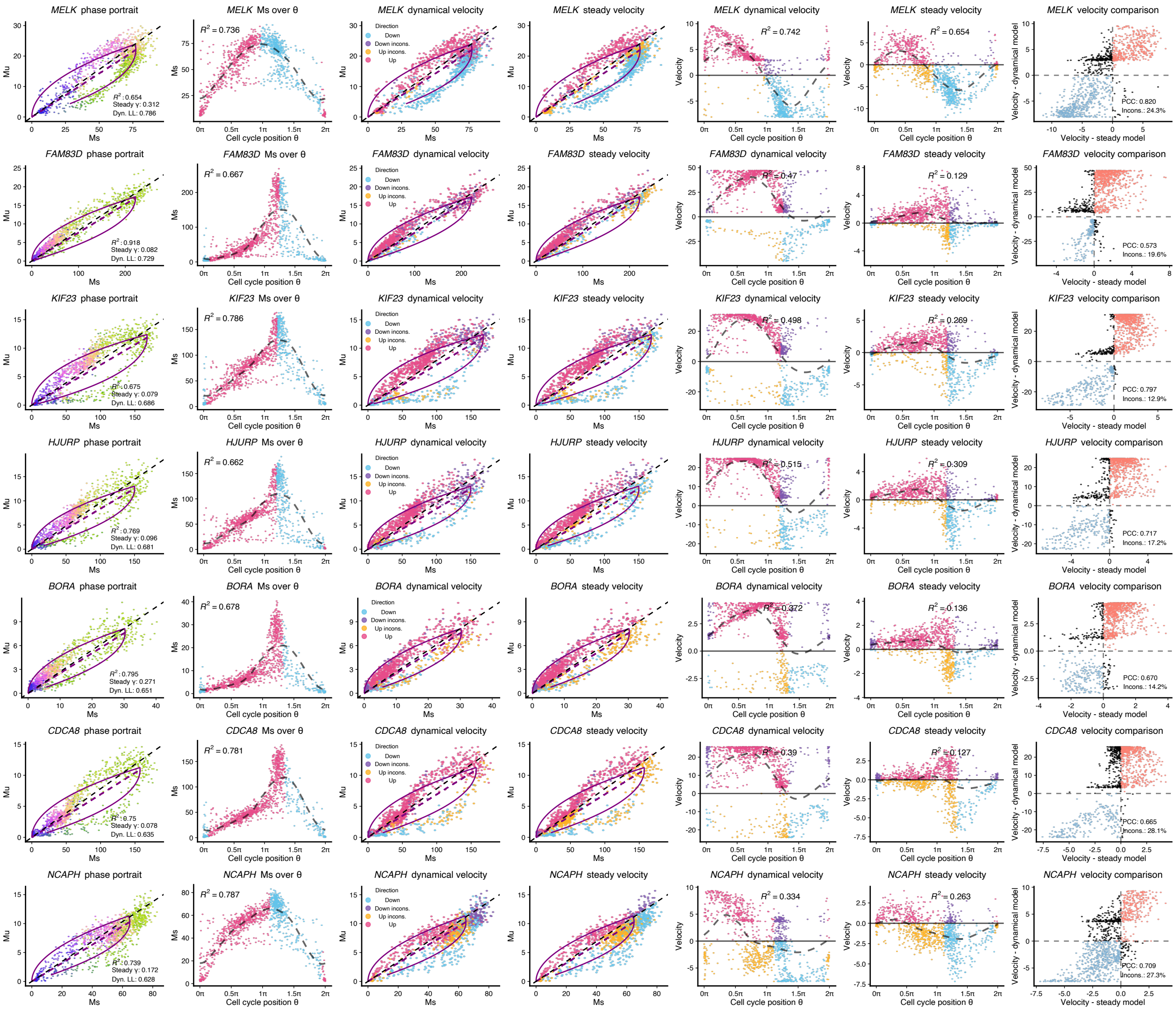

Fig. S13. Analyses of the ten best genes (highest likelihoods) in the FUCCI data. See the next page for captions.

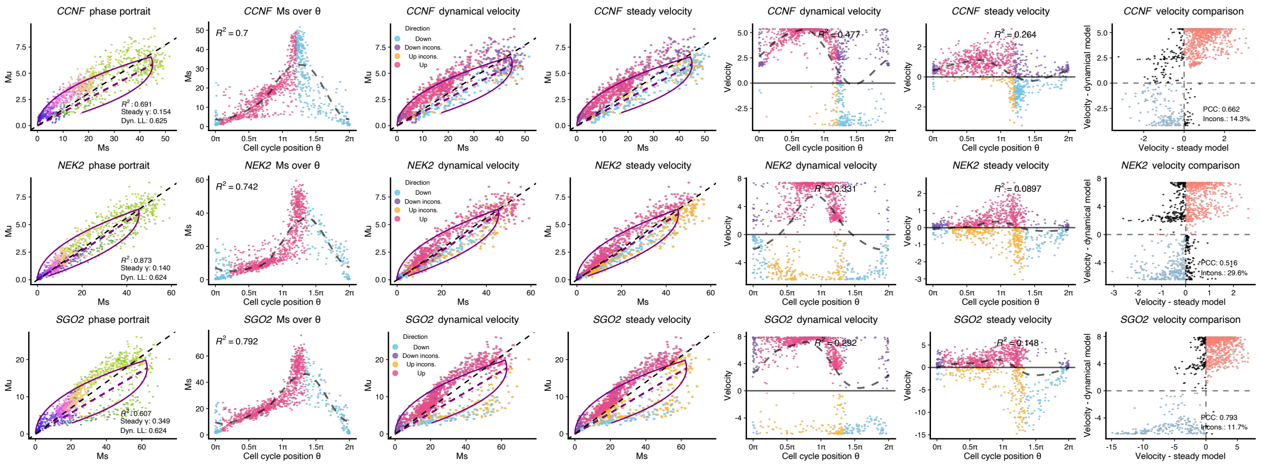

Fig. S13. (Continued) Each row contains a gene, ranked decreasingly by the likelihood. Each point represents a cell in the FUCCI data. From left to right of each row: phase portrait of the gene with points colored by cell cycle position; smoothed expression (Ms) over cell cycle position; phase portrait with points colored by direction comparisons between direction inferred by Ms and dynamical model based velocity estimates; phase portrait with points colored by direction comparisons between direction inferred by Ms and steady-state model based velocity estimates; dynamical model based velocity estimates over cell cycle position; steady-state model based velocity estimates over cell cycle position; comparison of velocity estimates using the steady-state model and the dynamical model. (Abbreviations: PCC: Pearson's correlation coefficient; NRMSE: Normalized Root Mean Square Error.)

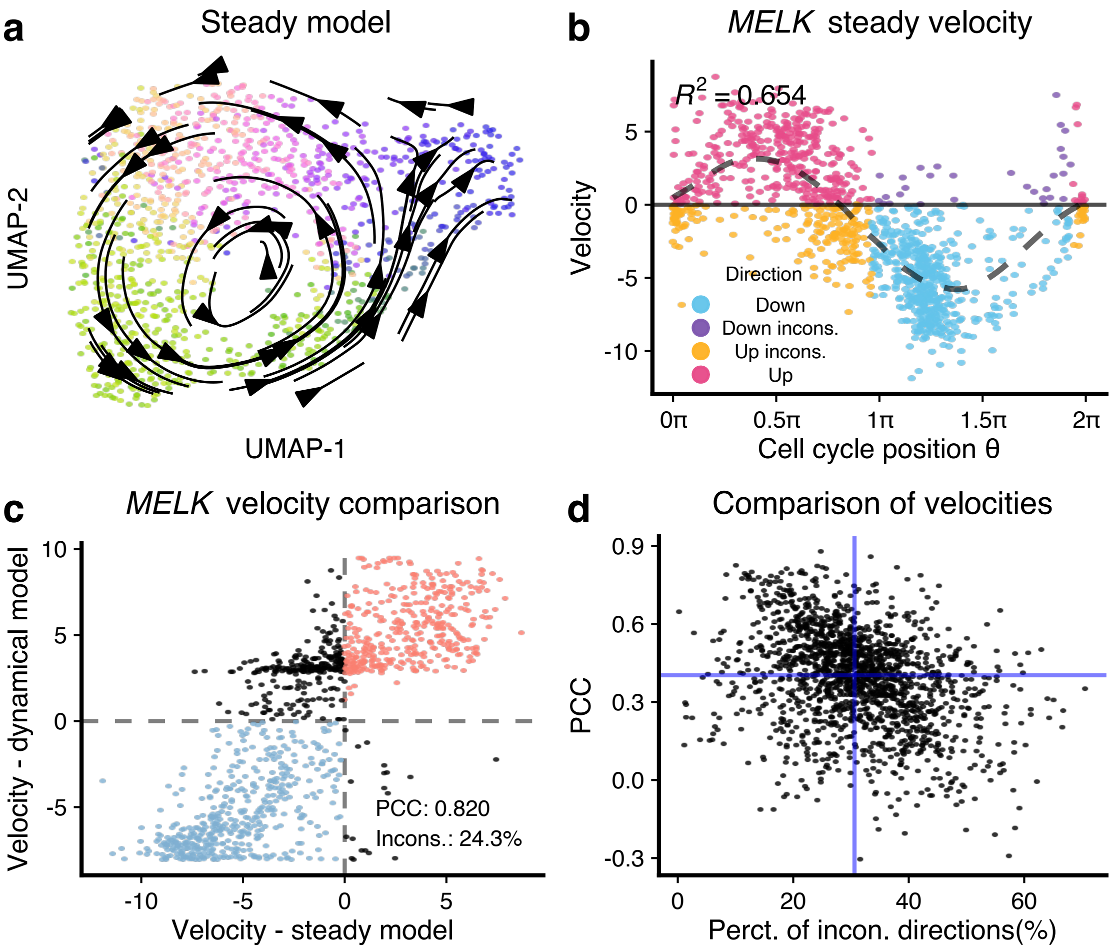

Fig. S14. RNA velocity estimation using the steady-state model on the FUCCI data and comparisons of gene-level velocities between steady-state and dynamical model. (a) Similar to Fig. 7a, but we now use RNA velocity estimates using the steady-state model. RNA velocity vector fields are visualized using the transition probability method on the UMAP embeddings of the FUCCI data. Each point represents a cell and is colored by its FUCCI pseudotime. **(b)** Scatter plot shows the estimated RNA velocity of *MELK* over FUCCI pseudotime. The signs of velocity estimations are compared to those inferred in Fig. 7c, with inconsistent directions colored black. **(c)** Comparison of RNA velocity for gene *MELK* estimated by steady-state model and dynamical model. About 24.3% of cells, which are colored black, exhibit opposite velocities between the two models. **(d)** Scatter plot shows the PCC and percentage of inconsistent directions between velocities estimated by the steady-state model and dynamical model for all velocity genes in the FUCCI data. The two blue lines give the respective median values, with a PCC median of only about 0.4. (Abbreviations: PCC: Pearson's correlation coefficient; Incons.: inconsistent; Perct.: percentage.)

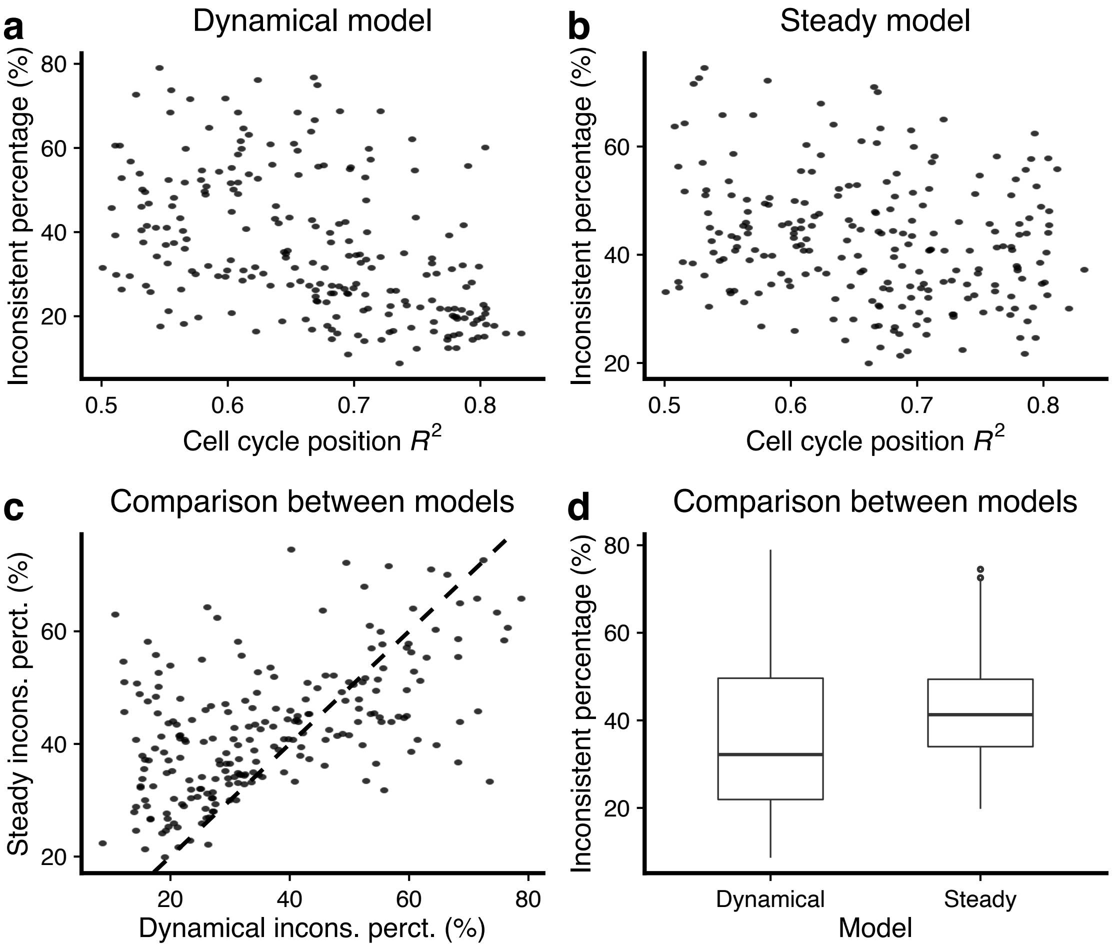

Fig. S15. Percentage of cells showing the inconsistent direction of change between fitted model using cell cycle position and gene-specific RNA velocity. Out of 579 cell cycle genes which are also velocity genes, we examine the 224 genes that have a R^2 of periodic loess over cell cycle position greater than 0.5. **(a)** Scatter plot shows the percentage of cells having the inconsistent direction of change between the fitted model using cell cycle position and dynamical model based gene-specific RNA velocity estimates. **(b)** As (a), but now we use the steady-state model to get RNA velocity estimates. **(c-d)** Comparison of the percentage of cells having the inconsistent direction of change between the steady-state and dynamical model velocity estimates.

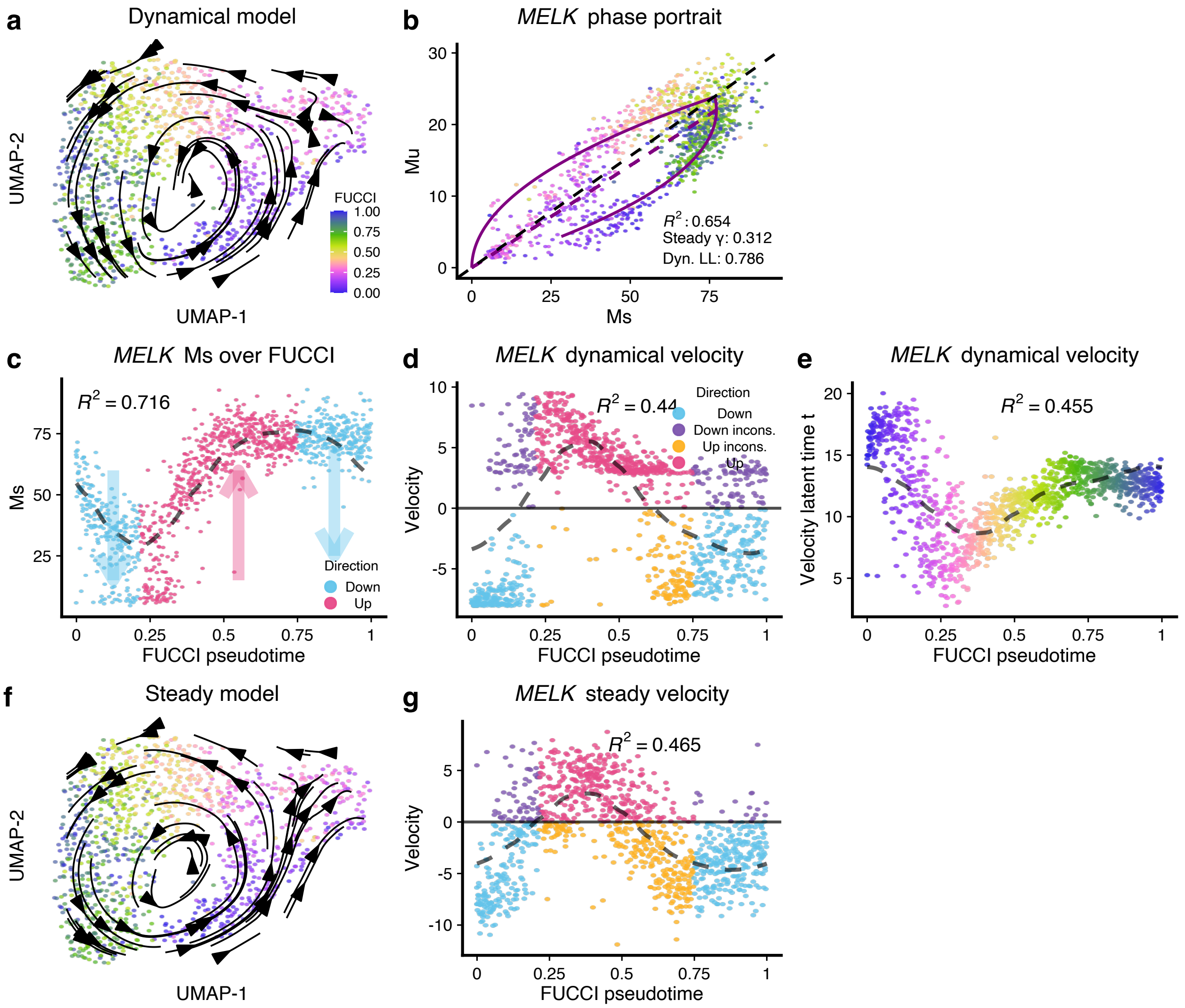

Fig. S16. The RNA velocity application on FUCCI dataset (colored by FUCCI pseudotime). (a-e) Similar to Fig. 7, but now we use FUCCI pseudotime as the "true" trajectory. **(a)** RNA velocity vector fields are visualized using the transition probability method on the UMAP embeddings of the FUCCI data. Each point represents a cell and is colored by FUCCI pseudotime. **(b)** Phase portrait of gene *MELK*, of which the likelihood is the highest among all velocity genes inferred by the dynamical model. The purple lines represent the dynamics inferred by the dynamical model. **(c)** Scatter plot shows smoothed expression of *MELK* over FUCCI pseudotime. The dashed line is the fitted line by periodic loess (Methods). The expected direction of change is inferred on the fitted loess line and visualized by colors. **(d)** Scatter plot shows the estimated RNA velocity of *MELK* over FUCCI pseudotime. The signs of velocity estimates are compared to those inferred in (c), with inconsistent directions colored black. **(e)** The level of agreement between the velocity latent time for *MELK* and the FUCCI pseudotime is lower than between the velocity latent time and the cell cycle position. **(f)** As (a), but we use the steady-state model velocity estimates. **(g)** As (d), but we use the steady-state model velocity estimates. (Abbreviations: Dyn.: dynamical; incons.: inconsistent.)

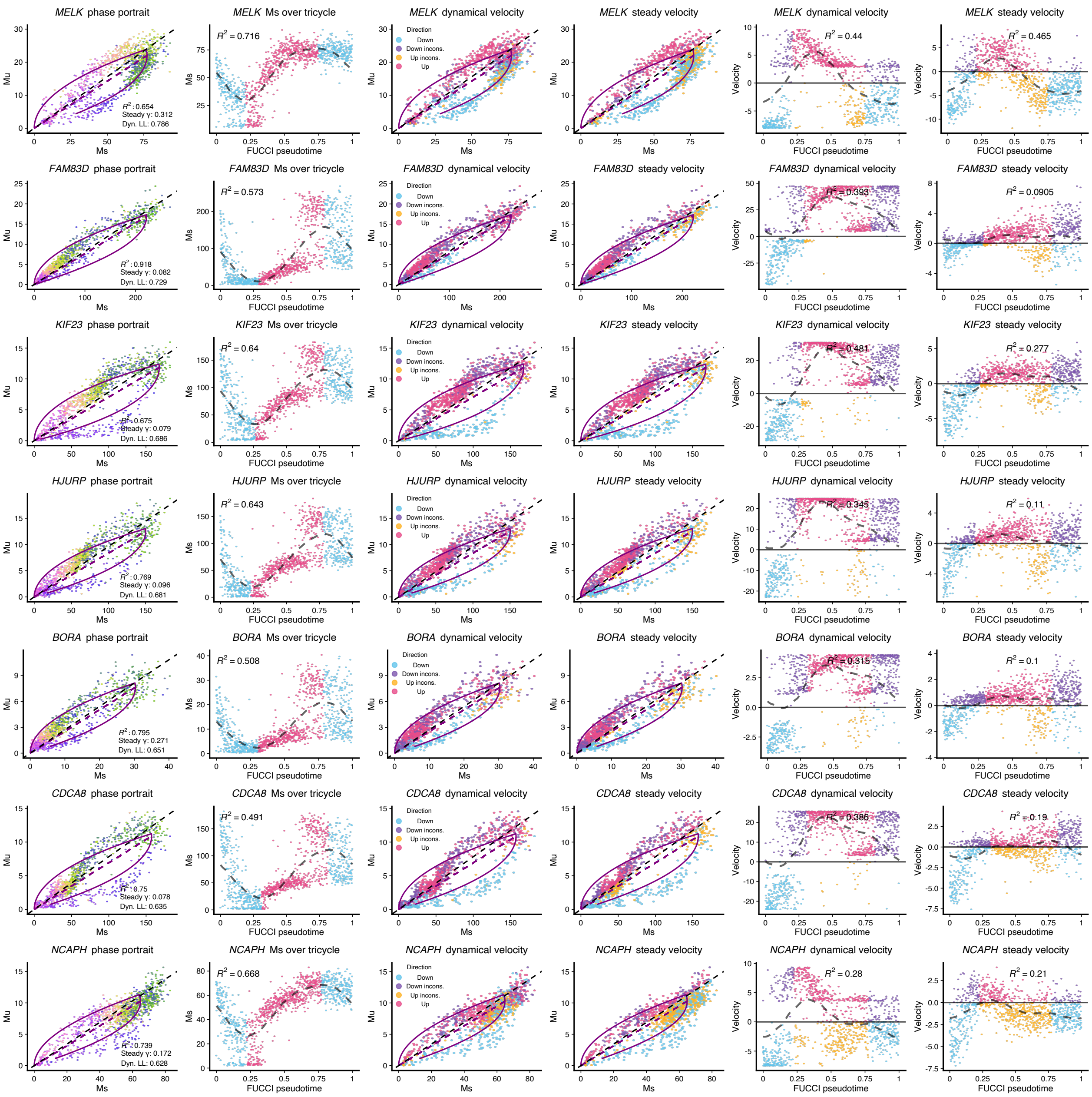

Fig. S17. Analyses of the 10 best genes (highest likelihoods) in the FUCCI data (using FUCCI pseudotime). See the next page for captions.

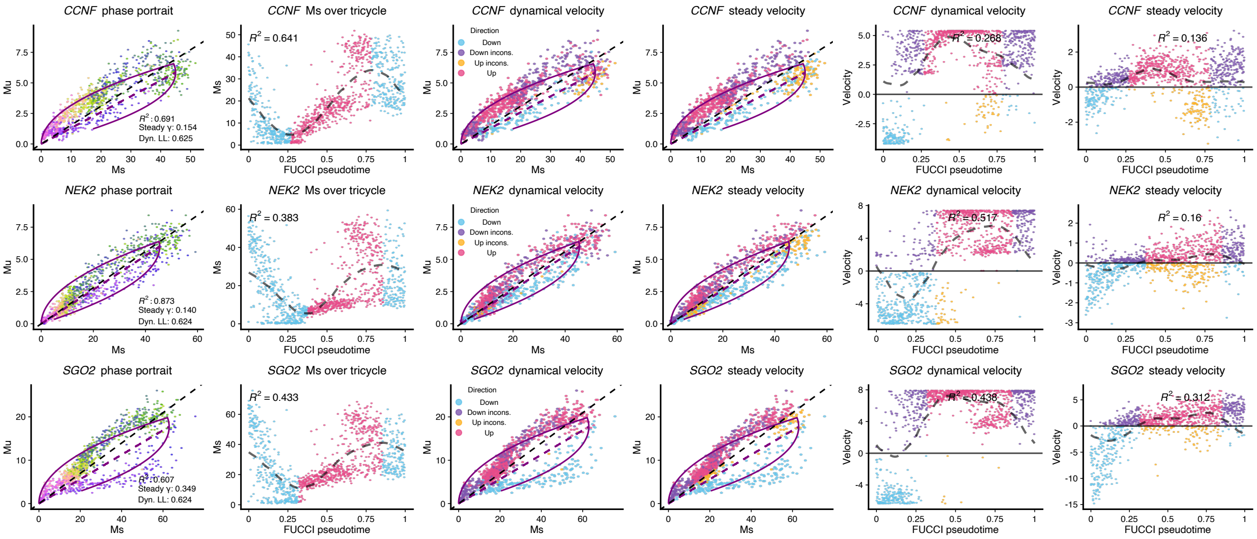

Fig. S17. (Continued) This figure is similar to Fig. S13, but now we use FUCCI pseudotime as the "true" trajectory. Each row contains a gene, ranked decreasingly by the likelihood. Each point represents a cell in the FUCCI data. From left to right of each row: phase portrait of the gene with points colored by FUCCI pseudotime; smoothed expression (Ms) over FUCCI pseudotime; phase portrait with points colored by direction comparisons between direction inferred by Ms and dynamical model based velocity estimates; phase portrait with points colored by direction comparisons between direction inferred by Ms and steady-state model based velocity estimates; the dynamical model based velocity estimates over FUCCI pseudotime; the steady-state model based velocity estimates over FUCCI pseudotime. (Abbreviations: Dyn.: dynamical; incons.: inconsistent.)

2 SUPPLEMENTARY NOTES

2.1 The differences between implementations of RNA velocity analysis

Aside from the fact that scVelo offers multiple models for high-dimensional RNA velocity estimations, while velocyto (Python and R packages) only implements a steady-state model, the default workflows of these three packages significantly differ. In this article, we will discuss some key differences we believe are important.

During the high-dimensional gene-level RNA velocity estimation step, all implementations smooth (also referred to as imputation) the spliced and unspliced counts using a k-NN graph; however, the specifics vary. The scVelo package constructs a k-NN graph using the Euclidean distance between cells in a low-dimensional PCA space (derived from the spliced matrix) and follows with a weighted smoothing step, with weights computed as in McInnes et al. (2018). Both parameter estimation and velocity computation utilize these smoothed values. The Python implementation of velocyto also builds a k-NN graph using Euclidean distance in a low-dimensional PCA space (using a different k-NN implementation from scVelo), but then performs unweighted smoothing. Both parameter estimation and velocity computation rely on these smoothed values. In contrast, the R implementation of velocyto employs an unweighted k-NN graph using the correlation distance on the high-dimensional spliced counts matrix. Neighboring cells are aggregated into pseudo cells, and a quantile regression line is fitted on these pseudo cells to obtain the estimated degradation rate. After calculating the degradation rate, velocities are computed for each gene by plugging in the unspliced and spliced counts (before forming pseudo-cells) in Equation 2; this represents a substantial deviation from the Python implementation.

Another significant difference lies in selecting the number K for the k-NN graph. The default parameters used in the scVelo package are $k = 30$ neighbors. For velocyto, there is no default k, but the analyses presented in La Manno et al. (2018) occasionally use large values (e.g., $k = 550$ for a forebrain dataset with fewer than 2,000 cells). As k is the most crucial parameter for all k-NN graphs, which is central to the high-dimensional RNA velocity estimation step, the choice of k may significantly impact the visualized low-dimensional vector field. For example, we compare the RNA velocity vector field of the Forebrain data using $k = 30$ and $k = 550$ for both steady-state and dynamical models (using the scVelo implementation for both models to avoid differences caused by other steps). Comparing Fig. S18a to Fig. S18b, we observe that a larger k in the steady-state model appears to yield a smoother vector field. However, a striking difference is observed for the dynamical model, where the low-dimensional vectors of neuroblast cells (purple and red cells) display the opposite direction when using $k = 30$ (Fig. S18c) compared to using $k = 550$ (Fig. S18d). As there is no guidance on selecting the k for a new dataset, we have consistently used $k = 30$, the default in the scVelo package.

There are notable differences between the implementations in the low-dimensional vector field visualization step. A prominent example is the construction of another k-NN graph using the low-dimensional embedding in velocyto, which results in a Pearson correlation matrix (compared to the cosine similarity matrix in scVelo). In contrast, scVelo uses the same k-NN graph for both processing (smoothing) and low-dimensional velocity vector field visualization. For velocyto, the resulting transition probability matrix is embedding-dependent, and having an embedding-dependent property for a transition probability matrix seems counterintuitive.

The three implementations differ significantly in critical steps, and the impact of these choices on the resulting output, including both high-dimensional velocities and low-dimensional vector fields, has not been thoroughly examined. We will not delve further into this issue. Instead, we will exclusively use the scVelo package for both the steady-state and dynamical models to circumvent implementation differences.

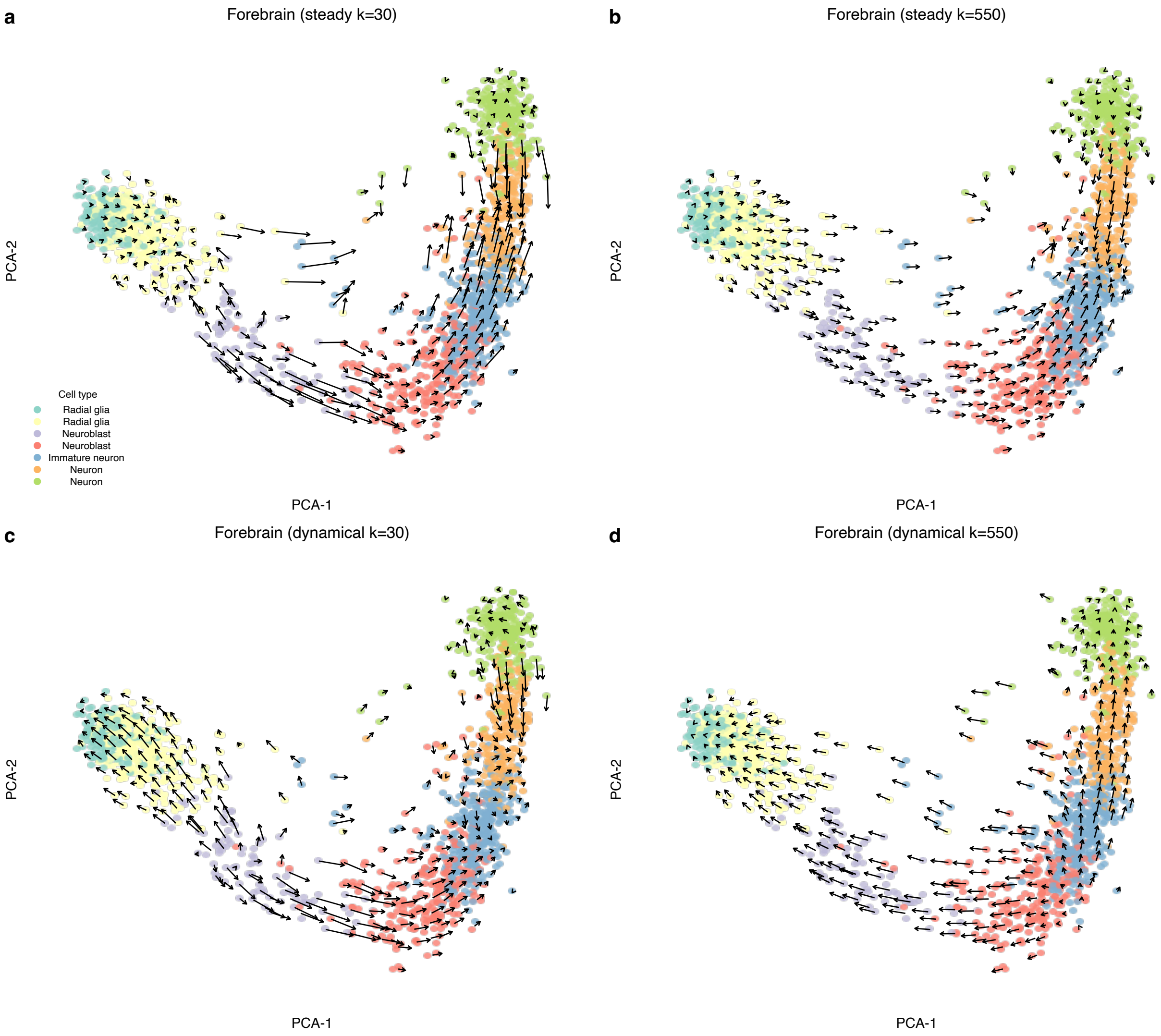

Fig. S18. Mapped RNA velocity of the Forebrain data using different numbers of K for the k-NN graph. (a) Mapped steady-state model based RNA velocity using the transition probability method of the Forebrain dataset when $k = 30$ for the k-NN graph. **(b)** As (b), but now we use $k = 550$, which was used by La Manno et al. (2018). **(c-d)** As (a-b), but now we use the dynamical model to estimate RNA velocity instead.

2.2 Visualization of 2d vector fields

To avoid overplotting, we usually summarize vectors in a 2D space, which can hide local detail. One approach is to grid the 2d embedding and compute the average vectors at each grid location. An alternative is the streamline plot which has multiple distinct implementations (The Matplotlib development team, 2022; Campitelli, 2022). Fig. S19a shows a streamline plot of a forebrain dataset previously discussed in the literature on RNA velocity (La Manno et al., 2018; Gorin et al., 2022). The gridding display of the same vector field produces a very different impression (Fig. S19b). If we examine the embedding by the left, middle, and right parts, the streamline plot hides that the length of vectors on the left is much shorter than the length of vectors in the middle.

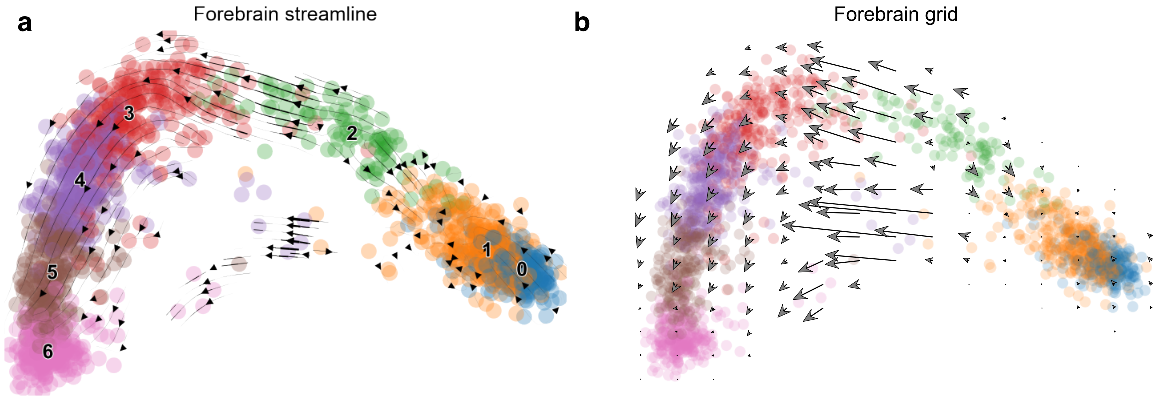

Fig. S19. The impact of different visualization approaches. We run scVelo on the Forebrain data and visualize the exact same vector field using two visualization functions pl.velocity_embedding_stream and pl.velocity_embedding_grid in scVelo, which give us quite different impressions of how the vector field looks like. **(a)** The vector field is visualized by streamline plot (The Matplotlib development team, 2022). We use all default parameters for the pl.velocity_embedding_stream function. **(b)** The vector field is visualized by gridding and kernel smoothing. We had to decrease the resolution (density=0.3), increase the arrow size (arrow_size=4), and scale up the arrow length (arrow_length=3) to make the arrows visible. Note that in the middle part, we see some long arrows, while the arrows on the other parts are fairly short.

In addition to the choice of visualization approaches, options such as resolution also matter. Not only does the different selection of resolution change the aesthetic impression, but it also affects the interpretation of the vector fields (Fig. S20), especially locally for some regions in the embedding, such as the top right part of the pancreas data.

These illustrate how the qualitative impression of a vector field depends on the visualization method. As the qualitative impression is usually subjective and highly parameter-dependent, we will not pursue this critical point further. But we strongly recommend using the same visualization tool with choices of appropriate parameters to compare different vector fields. We tend to favor the gridding approach, as it more faithfully depicts local vector fields than the streamline plot and is easier to reason.

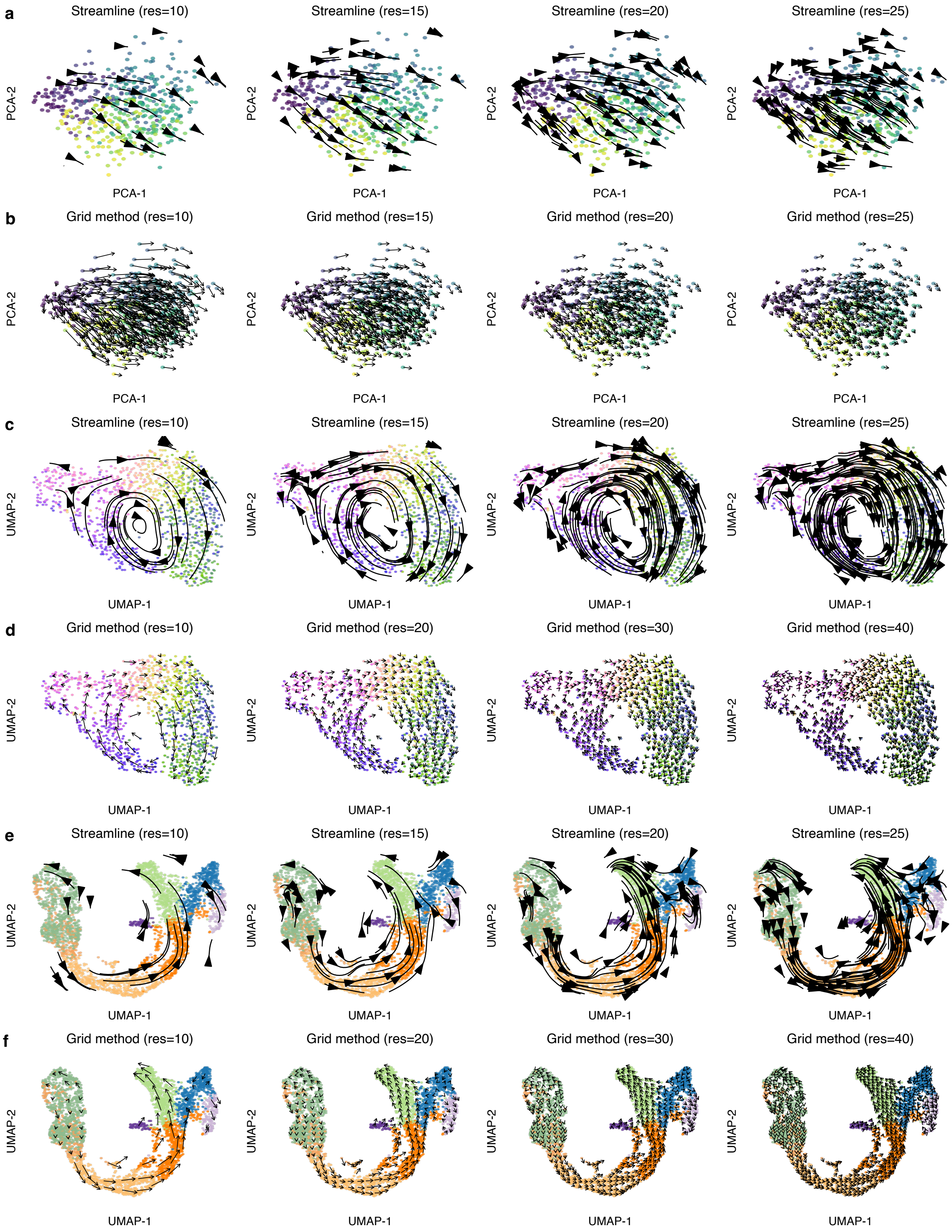

Fig. S20. The choice of resolution could affect how the RNA velocity vector field looks. We use three datasets to show that the choice of resolution could affect how the RNA velocity vector field looks like: simulated data in (a-b); FUCCI data in (c-d); pancreas data in (d-e). For those datasets, we used the dynamical model RNA velocity estimates. We use the streamline method to visualize the velocity vector field in (a), (c), and (e), while we use the grid method in (b), (d), and (f). From left to right, we increase the level of resolution. Note that how good the vector field looks depends on the subjectively optimal choice of resolution level, which is difficult to decide and varies across datasets.

REFERENCES

Campitelli, E (2022). *metR: Tools for Easier Analysis of Meteorological Fields*. https://cran.r-project.org/web/packages/metR/index.html.

Gorin, G, Fang, M, Chari, T, and Pachter, L (2022). RNA velocity unraveled. bioRxiv, 2022.02.12.480214. DOI: 10.1101/2022.02.12.480214.

La Manno, G, Soldatov, R, Zeisel, A, Braun, E, Hochgerner, H, Petukhov, V, Lidschreiber, K, Kastriti, ME, Lönnerberg, P, Furlan, A, et al. (2018). RNA velocity of single cells. Nature *560*, 494–498. DOI: 10.1038/s41586-018-0414-6.

McInnes, L, Healy, J, and Melville, J (2018). UMAP: Uniform Manifold Approximation and Projection for Dimension Reduction. arXiv, 1802.03426. DOI: 10.48550/arXiv.1802.03426.

The Matplotlib development team (2022). *Streamplot*. URL: https://matplotlib.org/stable/gallery/images_contours_and_fields/plot_streamplot.html (visited on 02/21/2022).
