# Supplementary material for: Pumping the brakes on RNA velocity by understanding and interpreting RNA velocity estimates
Source: Genome Biol. 2023 Oct 26;24:246. doi: 10.1186/s13059-023-03065-x (PMC10601342; doi:10.1186/s13059-023-03065-x)
Supplement: Supplementary file 2 — Additional file 2. Contains the review history. [file 13059_2023_3065_MOESM2_ESM.docx]

**1^st^ round**

**Reviewer 1**

SUMMARY

In this manuscript, the authors provide a thorough explanation and critique of modern RNA velocity estimation methods and how they are used in practice. This critique is thorough and well-supported at every step.

Making use of real data and popular existing methods, the authors show that the low-dimensional vector field is not necessarily representative of high-dimensional velocity estimates, and can be restricted to the space of cells present in the dataset. The latter is a severe limitation of RNA velocity analysis, as a qualitative assessment of the low-dimensional vector field is often the primary output from these methods. A seemingly coherent vector field may be held up as evidence of biological insights, but the authors show that actually, these methods may be incapable of producing incoherent results.

They also demonstrate that expression speed (the magnitude of velocity) is poorly estimated by existing methods and, indeed, low-dimensional speed is entirely unrelated to high-dimensional speed when using transition probabilities.

They make use of simulated data to tease out the sources of various discrepancies in a scenario where the true k-NN graph and velocities are knowable. This allows them to suggest a potentially useful metric based on R-squared values from loess models that could help identify datasets with high levels of transcriptional noise on which RNA velocity methods may perform poorly.

They also present a novel analysis of a cell cycle dataset, showing that it is particularly well-suited to velocity analysis. However, they detail multiple reasons why these characteristics are unlikely to generalize to other datasets.

COMMENTS

I don't have any concerns that rise to the level of "major", so consider these all to be "minor" comments.

1. This is likely not the authors' fault, but I find some of the terminology a bit unclear and I think there is some room for improvement here. In particular, I think the authors could make more use of the fact that "speed" is the magnitude of velocity, rather than using wordier phrases like "(velocity) vector length". Similarly, a single gene's contribution to the high-dimensional velocity vector is (if I understand correctly) a scalar value and could therefore be described as a "speed". This could stand to be more thoroughly explained and thereby potentially avoid some confusion between that quantity and the "gene-level velocity", ie. high-dimensional velocity. I found certain figures hard to decipher at first because there would be an axis labeled (ie.) "Dynamical v.", but it was referring to a scalar quantity (the speed of a single gene), not a vector quantity.

2. In general, I think the direction of the low-dimensional velocity vector is going to be more highly emphasized than the magnitude, so I would have liked to see more results from simulated data like panel 4(g). A similar panel with a different noise level, such as in Figure S4(q), could be promoted to a main figure.

3. The notation E and E* was introduced without explanation. Given that "E" is commonly used in statistics to denote expected value, it would be helpful to explain what it represents in this context (I think it's "embedding"?).

4. Whenever a single-gene speed is plotted ("Dynamical v."), a reference line at 0 could be helpful.

5. In general, figures and figure captions should explain more of the notation used within the figure. For example, "trans.pro" is used without explanation in Figure 4.

6. Several figures are hard to interpret due to the use of "v." as an abbreviation for "velocity". This is a common abbreviation for "versus", which makes titles like "True v. projection" (3d) very hard to decipher. I would encourage the use of a bold letter "v" or a "v" with an arrow over it when referring to "velocity" in figures.

7. This may be something I missed, but in the simulation study, why do cells have different speeds (as indicated by Figure 3g)? I was assuming they were homogeneous with respect to speed, but this doesn't seem to be the case.

8. For Figures 3b-e, it would be helpful to use two different colors for the dashed boxes. Otherwise, it is not clear which red box is which in panels (c) and (e).

9. In Figure 7(e), there is no legend indicating which boxplots refer to R-squared values for spliced/unspliced expression.

10. I don't know if it's necessary to repeat certain definitions (such as "velocity vector field", which has been used throughout) at the beginning of the Discussion.

11. The caption of Figure S1 mentions red and green rectangles, but the figure only contains black and light blue ones. There is also a caption for a non-existent panel (d).

**Reviewer 2**

In the manuscript, "Pumping the brakes on RNA velocity - understanding and interpreting RNA velocity estimates" by Zheng et al, the authors perform a systematic dissection of the limitations associated with the RNA velocity workflow. Furthermore, they highlight specific examples where RNA velocity estimation or velocity projection for visualization of the vector field may be particularly challenging to infer or well-suited according to the biological system. For example, the authors highlight how a noisy kNN graph can impact gene level estimates (e.g. moments, velocity, directionality) as well as visualization of RNA velocity vectors on a low dimensional embedding (e.g. with transition probabilities or projected vectors). Furthermore, they carefully dissect where noise impacts RNA velocity estimation and highlight this occurs at the level of preprocessing when computing spliced and unspliced moments vs. computing transition probabilities. In contrast, they illustrate an example where RNA velocity works well (e.g. cell cycle). As such, this analysis and evaluation is broadly applicable to the scientific community that uses RNA velocity, especially for visualization of cellular trajectories in noisy single-cell data. However, the manuscript has several issues that should be addressed prior to publication in Genome Biology. Many of the claims presented in the main text are overstated, should be more strongly supported by the data, or clarified. Below are the major and minor comments.

Major comments:

1. Page 1, lines 56-59: "the primary output of RNA velocity is a vector field visualized on a low dimensional embedding, usually a TSNE or UMAP." Page 2, lines 6-9: "the intermediate output towards the low dimensional vector field is the high dimensional estimation of ds/dt." These statements are misleading and should be clarified throughout the text. RNA velocity uses a kinetic model to estimate transcriptional dynamics, or the conversion between unspliced and spliced mRNA molecules. The primary output is the high-dimensional estimation of ds/dt, kinetic rate parameters, and latent time. This can also be used for identifying dynamic gene drivers or for visualization of the vector field. Moreover, high dimensional RNA velocity vectors have been used for more than qualitative visual interpretation. They have been used quantitatively for predicting cell fate probabilities, gene regulatory networks, embeddings, cellular progression etc, thus these approaches should also be referenced and cited.

2. In the abstract, the authors claim that, "RNA velocity is at best a smooth interpolation of the observed k-NN structure as opposed to an extrapolation of future cellular states." How exactly is it an interpolation and are there experiments/data to support this? Moreover, it should be clarified if this is in reference to the visualization of the vector field using transition probabilities or the high dimensional RNA velocity estimates. Nevertheless, high dimensional RNA velocity vectors do provide directional information generated from spliced and unspliced counts, even if moments are computed from a kNN graph of observed spliced expression.

3. One of the major findings of this manuscript is that transition probabilities fail to correctly estimate speed (i.e. length) when comparing low dimensional velocity vectors to the simulated ground truth. Transition probabilities were designed to place the vector in the direction in which the expression difference is best correlated with the estimated velocity vector for visualization, not to capture speed or length (La Manno et al. Nature 2018). The dynamical model implementation (Bergen et al. Nature 2020) states that transition probabilities solely measure direction, not magnitude (ie speed). Lange et al. Nature 2022 highlights that transition probabilities reflect transcriptional similarity as well as local changes in directional information given by RNA velocity.

4. Throughout the manuscript, the authors claim that "RNA velocity does not estimate speed at the cell or gene level;" however, they demonstrate clear examples where RNA velocity works well. For example, high dimensional velocity estimation under low noise conditions have high PCC and low NRMSE (e.g. gene level Figure S7 noise condition 1 and 2) and high cosine similarity amongst transition probabilities or projected vectors using a denoised kNN graph (e.g. Figure 4g), speed of projected low dimensional vectors have high correlation with true high dimensional speed (Figure 3g), cell cycle (Figure 6).

5. In reference to Figure 2, the authors perform an ablation experiment by removing a population of beta cells prior to recomputing transition probabilities. Moreover, they illustrate how the kNN graph used for estimating transition probabilities imposes directional constraints, as it depends on the cells that were profiled. Although an informative illustration, this experiment can be considered a bit artificial. It might be more informative if the study highlighted how RNA velocity performs in scenarios where cells may be incompletely sampled due to technical limitations (e.g. downsampling populations vs. complete removal).

6. The authors should provide details on how the quantification is performed in Figure 2d in the methods section.

7. In reference to Figure 3, the authors describe a simulation experiment generating 500 cells and 10 genes with low noise levels. Why is it super important that the resulting qualitatively similar vector field has differences around the start of the trajectory?

8. To illustrate how well projected RNA velocity vectors capture directionality, the authors should compute the cosine similarity between (1) projected low dimensional vectors and high dimensional vectors and (2) transition probability low dimensional vectors and high dimensional vectors and (3) high-dimensional dynamical velocity and the true velocity vectors for simulated data under low noise conditions, as in Figure 3. The latter is particularly important to mention as this is what RNA velocity is designed for.

9. PCC should be clearly defined and described in methods.

10. On page 2, lines 4-5, the authors mention that the RNA velocity vector field is used to provide evidence for the correctness of the embedding. This is further mentioned on Page 18, lines 21. Can you clarify / provide references where/how the visualized vector field is used to validate an embedding?

11. On page 3, line 27: 'RNA velocity analysis results are usually qualitative' - could you clarify how exactly this is validated in this qualitative manner?

12. On page 9, line 16: 'the kNN graph learned from the data is perturbed away from the true graph' - what does true graph mean? Point related to this, the trueKNN, learned kNN, trans true kNN acronyms in Figure 4 could be defined in a clearer way.

13. The introduction could benefit from a bit more organization to be more digestible for readers that are less familiar with the history and terminology used in the RNA velocity field. I think that the introduction could become much more digestible if it was broken into subsections (description of the technology, dynamical vs steady state model, inference techniques, possible decisions or components of various inference techniques that you are planning to evaluate in your paper).

14. The discussion could benefit from a clearer explanation of where RNA velocity works and fails. It currently highlights speed limitations; however, the authors should highlight correctness of high dimensional directionality, speed, and parameter estimates under low noise conditions, as well as the correctness of directional mapping under low noise conditions. It seems that the limitation is with using a kNN graph that is not representative of the underlying biological process (overwhelmed by noise, missing cell populations) vs RNA velocity estimation itself.

Minor comments and typos:

1. Page 2, lines 9-10: "the manuscript primarily discusses the python implementation of velocyto." Page 3, lines 44-46: "here, we primarily focus on the newer dynamical model as implemented in scVelo". A distinction should be made between how spliced and unspliced counts are being quantified (e.g. velocyto pipeline) vs. how RNA velocity is being estimated (e.g. scVelo steady-state or dynamical model). Furthermore, it would be useful to explain what is happening in velocyto and not just that the manuscript discusses a python implementation.

2. The acronym kNN is used frequently throughout the text and is never formally defined. It is best to define this as k nearest neighbor graph.

3. In the 'construction of the k-NN graph' section in the methods on page 19, lines 56-57, the authors should describe what the default parameters are. How many neighbors were considered? This is mentioned in the supplemental file (k = 30), but should be also included in the main text for clarity.

4. Abstract, line 37, 'This dependence lead' → 'This dependence leads to'

5. Page 2, line 47: 'highlight examples where RNA velocity vector is not compatible' → 'highlight examples where the RNA velocity vector is not compatible'

6. Figure 4g: 'consine similarity' → 'cosine'

**Authors’ response**

Reviewer #2

“In this manuscript, the authors provide a thorough explanation and critique of modern RNA velocity estimation methods and how they are used in prac tice. This critique is thorough and well-supported at every step.

Making use of real data and popular existing methods, the authors show that the low-dimensional vector field is not necessarily representative of high-di mensional velocity estimates, and can be restricted to the space of cells present in the dataset. The latter is a severe limitation of RNA velocity analysis, as a qualitative assessment of the low-dimensional vector field is often the primary output from these methods. A seemingly coherent vector field may be held up as evidence of biological insights, but the authors show that actually, these methods may be incapable of producing incoherent results.

They also demonstrate that expression speed (the magnitude of velocity) is poorly estimated by existing methods and, indeed, low-dimensional speed is entirely unrelated to high-dimensional speed when using transition probabil ities.

They make use of simulated data to tease out the sources of various discrep ancies in a scenario where the true k-NN graph and velocities are knowable. This allows them to suggest a potentially useful metric based on R-squared values from loess models that could help identify datasets with high levels of transcriptional noise on which RNA velocity methods may perform poorly.

They also present a novel analysis of a cell cycle dataset, showing that it is particularly well-suited to velocity analysis. However, they detail multiple reasons why these characteristics are unlikely to generalize to other datasets.”

**Response:** Thanks a lot for the encouraging comments. And thanks for the effort spent on following our arguments. We believe our revised version is easier to follow.

“COMMENTS

I don’t have any concerns that rise to the level of ”major”, so consider these all to be ”minor” comments.

1. This is likely not the authors’ fault, but I find some of the terminology a bit unclear and I think there is some room for improvement here. In partic ular, I think the authors could make more use of the fact that ”speed” is the magnitude of velocity, rather than using wordier phrases like ”(velocity) vec tor length”. Similarly, a single gene’s contribution to the high-dimensional velocity vector is (if I understand correctly) a scalar value and could therefore be described as a ”speed”. This could stand to be more thoroughly explained and thereby potentially avoid some confusion between that quantity and the ”gene-level velocity”, ie. high-dimensional velocity. I found certain figures hard to decipher at first because there would be an axis labeled (ie.) ”Dynami cal v.”, but it was referring to a scalar quantity (the speed of a single gene), not a vector quantity.”

**Response:** We now clarify our discussion of speed by adjusting our notation. We differ entiate the high-dimensional velocity vector of cell *c* as *⃗v_c_*, the velocity vector of gene *g* across all cells as *⃗v_g_*, and the comprehensive velocity for all genes and cells as **V**.

“2. In general, I think the direction of the low-dimensional velocity vector is going to be more highly emphasized than the magnitude, so I would have liked to see more results from simulated data like panel 4(g). A similar panel with a different noise level, such as in Figure S4(q), could be promoted to a main figure.”

**Response:** We have now included the new main Figure 5, which is similar to Fig 4g, and illustrates the cosine similarities between the mapped low-dimensional cell-level veloc ity estimates and the mapped low-dimensional true cell-level velocity at various noise levels.

“3. The notation E and E* was introduced without explanation. Given that ”E” is commonly used in statistics to denote expected value, it would be helpful to explain what it represents in this context (I think it’s ”embedding”?).”

**Response:** We now explain what *E* operator does in our manuscript as ”We could use *E*(*⃗y*) to represent mapping a high-dimensional vector *⃗y* in a low-dimensional space.”

“4. Whenever a single-gene speed is plotted (”Dynamical v.”), a reference line at 0 could be helpful.”

**Response:** We have now added a reference line at 0 when we plot out the velocity value for a single gene.

“5. In general, figures and figure captions should explain more of the notation used within the figure. For example, ”trans.pro” is used without explanation in Figure 4.”

**Response:** We now explained all abbreviations used in the caption for each figure includ ing ”trans.pro” which is now explained in the caption of Figure 4.

“6. Several figures are hard to interpret due to the use of ”v.” as an abbreviation for ”velocity”. This is a common abbreviation for ”versus”, which makes titles like ”True v. projection” (3d) very hard to decipher. I would encourage the use of a bold letter ”v” or a ”v” with an arrow over it when referring to ”velocity” in figures.”

**Response:** We change our notation to distinguish the high-dimensional velocity vector of cell *c* as *⃗v_c_*, the velocity vector of gene *g* for all cells as *⃗v_g_*, and the velocity for all genes and all cells as **V**.

“7. This may be something I missed, but in the simulation study, why do cells have different speeds (as indicated by Figure 3g)? I was assuming they were homogeneous with respect to speed, but this doesn’t seem to be the case.”

**Response:** We simulate from the model; the differential equations impose the speed to be dependent on *s*(*t*), *u*(*t*), which are dependent on *t* (the latent time).

In the Methods section titled “Review of the Dynamical Model of RNA Velocity for scRNA seq Data”, we explain that the spliced count, unspliced count, derivative of the spliced count with respect to latent time *t*, and derivative of the unspliced count with respect to latent time *t* are all univariate functions of *t*. Consequently, to generate cells with vary ing expression values (spliced counts), we must simulate cells with different latent times. As illustrated in Figure 3a, the velocity value for an individual gene is dependent on the latent time *t*, which varies among cells.

“8. For Figures 3b-e, it would be helpful to use two different colors for the dashed boxes. Otherwise, it is not clear which red box is which in panels (c) and (e).”

**Response:** We have moved the relevant panels to the new Supplementary Figure S2 fol lowing a suggestion by Reviewer 2. We now use red and blue boxes in Supplementary Figure S2 to distinguish the ”projections” on the figure.

“9. In Figure 7(e), there is no legend indicating which boxplots refer to R squared values for spliced/unspliced expression.”

**Response:** We add the legend of Figure 8e (previously 7e) on the right side.

“10. I don’t know if it’s necessary to repeat certain definitions (such as ”ve locity vector field”, which has been used throughout) at the beginning of the Discussion.”

**Response:** We expect that some readers are skipping the Results section to go straight to the Discussion, and we want to make our definitions clear to such readers. However, our previous wording was unfortunate because it wasn’t really clear that we were just repeating a definition from our Results section. To reduce the confusion, we changed the wording to

“*We remind the reader that we use the term “velocity vector field” exclusively to refer to the low dimensional representation. This vector field is always relative to the choice of low-dimensional space and is easy to visualize.”*

“11. The caption of Figure S1 mentions red and green rectangles, but the figure only contains black and light blue ones. There is also a caption for a non existent panel (d).”

**Response:** We have fixed the inconsistency. The captions of Figure S1 now reads

“***Mapped RNA velocity in the pancreas dataset after removing a cell type. (a)*** *Dynamical model based RNA velocity is visualized on the UMAP for the pancreas data after removing the beta cells.* ***(b)*** *As (a), but the pre-endocrine cells are removed instead.* ***(c)*** *As (a), but the Ngn3 high EP cells are removed instead. Note that the arrows disappear for cells in the black rectangles of (b) and (c).”*

Reviewer 2

“In the manuscript, ”Pumping the brakes on RNA velocity - understanding and interpreting RNA velocity estimates” by Zheng et al, the authors perform a systematic dissection of the limitations associated with the RNA velocity workflow. Furthermore, they highlight specific examples where RNA velocity estimation or velocity projection for visualization of the vector field may be particularly challenging to infer or well-suited according to the biological sys tem. For example, the authors highlight how a noisy kNN graph can impact gene level estimates (e.g. moments, velocity, directionality) as well as visual ization of RNA velocity vectors on a low dimensional embedding (e.g. with transition probabilities or projected vectors). Furthermore, they carefully dis sect where noise impacts RNA velocity estimation and highlight this occurs at the level of preprocessing when computing spliced and unspliced moments vs. computing transition probabilities. In contrast, they illustrate an example where RNA velocity works well (e.g. cell cycle). As such, this analysis and evaluation is broadly applicable to the scientific community that uses RNA velocity, especially for visualization of cellular trajectories in noisy single-cell data. However, the manuscript has several issues that should be addressed prior to publication in Genome Biology. Many of the claims presented in the main text are overstated, should be more strongly supported by the data, or clarified. Below are the major and minor comments.

Major comments:

1. Page 1, lines 56-59: ”the primary output of RNA velocity is a vector field visualized on a low dimensional embedding, usually a TSNE or UMAP.” Page 2, lines 6-9: ”the intermediate output towards the low dimensional vector field is the high dimensional estimation of ds/dt.” These statements are misleading and should be clarified throughout the text. RNA velocity uses a kinetic model to estimate transcriptional dynamics, or the conversion between unspliced and spliced mRNA molecules. The primary output is the high-dimensional estimation of ds/dt, kinetic rate parameters, and latent time. This can also be used for identifying dynamic gene drivers or for visualization of the vec tor field. Moreover, high dimensional RNA velocity vectors have been used for more than qualitative visual interpretation. They have been used quan titatively for predicting cell fate probabilities, gene regulatory networks, em beddings, cellular progression etc, thus these approaches should also be refer enced and cited.”

**Response:** We acknowledge that our previous assertion that ”the primary output of RNA velocity is a vector field visualized on a low-dimensional embedding, usually a TSNE or UMAP” was inaccurate. We have made the necessary clarifications in the Introduction and Results sections while also emphasizing the significance of visualization in efforts to establish the usefulness of RNA velocity. For instance, a majority of the figures in both La Manno et al. (2018) and Bergen, Lange, et al. (2020) consist of low-dimensional vector fields.

We now write in Introduction,

“*RNA velocity analysis for single-cell gene expression data starts with a high-dimensional esti mation of ds*/*dt; we refer to these estimates as “velocities” (or gene-specific rates of change). In a second (optional) step, these high-dimensional velocities are visualized as a vector field on a low dimensional embedding, usually constructed using UMAP or t-SNE. This vector field is supposed to represent local changes in the expression state; one might describe the output as an alternative to trajectory inference analysis (Weiler et al., 2021). We will use “velocity vector field” to refer to the low-dimensional vector field obtained by mapping the velocities into a low-dimensional space. In many datasets, the visualization of the velocity vector field appears to reflect what is known about the biological system and the relationship between cell states/types in the system.”*

and in Results

“*RNA velocity analysis has two components, with the second component being optional*

*1. Spliced and unspliced counts are preprocessed (smoothed/imputed), and a cell-specific veloc ity is estimated separately for each gene.*

*2. (Optional) The high-dimensional velocity estimates are mapped into a low-dimensional em bedding, and the resulting vector field is visualized on this embedding.*

*Visualization of RNA velocity results through the construction of a low-dimensional vector field on a given embedding (optional component 2) is important for using RNA velocity to bring in sight into a biological system. Much – but not all – of the evidence for the usefulness of RNA velocity rests on its ability to produce low-dimensional vector fields which appear to represent known biology accurately. This suggests that it is important to understand the properties of this second component; what happens when high-dimensional velocity estimates are mapped to a low dimensional embedding, particularly using the transition probability method introduced by La Manno et al. (2018).”*

“2. In the abstract, the authors claim that, ”RNA velocity is at best a smooth interpolation of the observed k-NN structure as opposed to an extrapolation of future cellular states.” How exactly is it an interpolation and are there ex periments/data to support this? Moreover, it should be clarified if this is in reference to the visualization of the vector field using transition probabil ities or the high dimensional RNA velocity estimates. Nevertheless, high di mensional RNA velocity vectors do provide directional information generated from spliced and unspliced counts, even if moments are computed from a kNN graph of observed spliced expression.”

**Response:** Re-reading our abstract with a critical eye following this comment by the re viewer made us realize the need for more precision and clarity instead of the existing ”flowery” language. Specifically, throughout our work, we have emphasized the differ ence between low and high-dimensional velocity; we have introduced this difference in the abstract. In response to this, we have completely rewritten the abstract (including now following the Genome Biology style).

“3. One of the major findings of this manuscript is that transition probabilities fail to correctly estimate speed (i.e. length) when comparing low dimensional velocity vectors to the simulated ground truth. Transition probabilities were designed to place the vector in the direction in which the expression difference is best correlated with the estimated velocity vector for visualization, not to capture speed or length (La Manno et al. Nature 2018). The dynamical model implementation (Bergen et al. Nature 2020) states that transition probabilities solely measure direction, not magnitude (ie speed). Lange et al. Nature 2022 highlights that transition probabilities reflect transcriptional similarity as well as local changes in directional information given by RNA velocity.”

**Response:** In this comment, the reviewer puts length estimation into context and states that the foundational papers of the field contain clear statements cautioning against in terpreting low-dimensional vector length as speed.

We have now re-read the entirety of the two papers in great detail, including the Supple mentary Materials. We have supplemented this reading by searching for relevant key words. Below, we detail what we could find in explicit statements in the papers. If the reviewer believes we are missing something, we would very much appreciate an accurate quote so we can find the relevant passage.

Much of the wording and choice of terminology in the two papers suggest that speed is a component of the estimates – the usage of ”velocity” and model parameters which are de scribed as kinetic parameters. And this is emphasized in the text, for example, La Manno et al. (2018) writes, ”Here we show that RNA velocity – the time derivative of the gene expression state” (abstract), ”As RNA velocity is grounded in real transcription kinet ics...” and ”Most genes showed a positive correlation between velocity and empirically observed expression derivatives...”

It can be argued that these statements pertain to gene-level (high-dimensional) veloci ties and not to the velocity vector field. However, neither in La Manno et al. (2018) nor in Bergen, Lange, et al. (2020) is the interpretation of the length of the low-dimensional embedded vector (derived from the transition probability approach) clarified, and in our opinion, the visualizations often suggest that it carries biological information. For exam ple, the authors display low-dimensional vector fields that distinctly exhibit cell-specific lengths.

We also note that sometimes the authors of the foundational papers do seem to interpret vector lengths. For example, La Manno et al. (2018) clearly interprets vector length in Figure 11 (Supplementary Note 2). For this figure, they visualize vectors using PCA, but it is unclear if they use the projection operator or transition probabilities to embed the velocities. If the intention was to use PCA and the orthogonal projection operator to be able to interpret vector length (which we argue makes sense), they never explicitly state this. In the Results section of Bergen, Lange, et al. (2020), the authors appear to interpret (what we read as) vector lengths: ”Furthermore, the inferred velocities in *α*-cells are lower than the strong directional flow in *β*-cells, which, again, suggests that *α*-cells have already been produced at an earlier stage.” Now, perhaps the authors meant high-dimensional speed and not embedded vector length, but we believe it is very easy for readers to be confused. And we believe our paper adds substantially to this discussion by clarifying what may represent speed under which conditions.

In La Manno et al. (2018), we have not been able to find **any** statements cautioning against interpreting vector length after using transition probabilities. The closest is the following quote “Because cells can have RNA velocities along multiple independent components simultaneously, such as differentiation, maturation, and proliferation, care must be taken when interpreting low-dimensional representations, as cells that lack apparent velocity in one particular embedding can nevertheless have substantial velocity in some subspace that is not visualized.”

In the Methods of Bergen, Lange, et al. (2020), the authors write, “It solely measures sim ilarity in directionality, not in magnitude, and ranges from -1 (opposite direction) over 0 (orthogonal, thus maximally dissimilar) to 1 (identical direction).” when introducing cosine similarity in the section on transition probabilities. This is a very clear statement. However, there is a difference between cosine similarity, transition probabilities, and em bedded velocities which are described as “the embedded velocity is estimated as the ex pected displacements with respect to the transition matrix”; no explanation is offered for what the length of displacement represents.

In summary, in our opinion, while the foundational papers do touch on this issue, they offer no concrete interpretation of what the vector length represents in low dimensions (despite plotting vector length in their visualizations), and we argue it is very easy for readers/users to believe it represents speed. This is especially true for users who have not taken a deep dive into the inner workings of RNA velocity but are only using it to gain biological insights into their system of interest. Our work is an attempt to clarify these issues to the community.

“4. Throughout the manuscript, the authors claim that ”RNA velocity does not estimate speed at the cell or gene level;” however, they demonstrate clear examples where RNA velocity works well. For example, high dimensional ve locity estimation under low noise conditions have high PCC and low NRMSE (e.g. gene level Figure S7 noise condition 1 and 2) and high cosine similarity amongst transition probabilities or projected vectors using a denoised kNN graph (e.g. Figure 4g), speed of projected low dimensional vectors have high correlation with true high dimensional speed (Figure 3g), cell cycle (Figure 6).”

**Response:** We agree that Supplementary Figure S8 (previously S7) shows that for low noise levels, we get much better PCC and RSME compared to higher noise levels (al though we still get substantially better performance by using the ”true” k-NN in those noise level scenarios). Likewise, Supplementary Figure S11 (previously S9) shows decent performance for cosine of the cell-specific direction and good PCC of the speed for noise levels 1 and 2 (with noise level 2 being substantially worse than 1 on the speed). We have added text which highlights this observation to the Results section (see quote below), and we have added text about this to the Discussion (see question 14 by the reviewer, which asks for this).

We now write “*At very low noise settings (1 or 2), RNA velocity appears to work well; using the true k-NN graph still improves performance substantially.”* as well as “*For low noise levels (1 or 2), we observe a good PCC between estimated and true speed as well as good-to-decent cosine similarity between directions.”*

For Figure 7 (cell cycle; previously Figure 6), we again agree that RNA velocity estimated using the dynamical model has a very nice performance for *MELK*. However, we empha size that this is – by a good margin – the best gene. Supplementary Figure S13 (previously S11) shows the other top genes, and Supplementary Figure S14 (previously S12) shows the consistency in the direction of change (when the true direction of change is calculated using cell cycle time, which is a decent – but of course not perfect – a proxy for the truth). What we see for this particular data is that for many genes, the dynamical model and the steady state model disagree substantially (with the dynamical model being far bet ter). However, we believe we have already highlighted this for the cell cycle data; for example, we currently write in the Results

“*In summary, when it comes to the inference of direction of expression change, RNA velocity appears to work well in this cell cycle system, likely due to the fact that dynamic genes in this system experience both inductions and decrease over the course of the cell cycle.”*

Finally, we don’t agree that Figures 3g (previous numbering) and 4g (previous number ing) show that RNA velocity estimation works in low-noise settings. Specifically, Figure 3g does not depict the result of RNA velocity estimation. It takes true vectors (calculated based on simulation parameters, and depicts those true vectors visualized by PCA pro jection or transition probabilities. The expression data (not the velocities) are simulated with low noise, which impacts (1) the k-NN graph itself and (2) the UMAP / PCA, but the velocities (arrows) are not estimated using RNA velocity but are the ”true” velocities. For Figure 4g, we remind the reviewer that we are using the ”true” k-NN graph for both preprocessing and visualization. A major contribution to the result is that we smooth the expression data using the ”true” k-NN graph prior to using the model to estimate RNA velocity. Because we don’t use the k-NN graph for visualization using PCA projection, this shows that the major contribution is to use the k-NN graph to smooth the data. We don’t think it is fair to say that this shows RNA velocity to work well when it requires preprocessing the data using the unknown truth.

In response to this, we have also changed the Discussion, and the quotes and details are described in our answer to major issue 14, which now includes a discussion of whether noise levels 1 or 2 are realistic.

“5. In reference to Figure 2, the authors perform an ablation experiment by removing a population of beta cells prior to recomputing transition probabil ities. Moreover, they illustrate how the kNN graph used for estimating tran sition probabilities imposes directional constraints, as it depends on the cells that were profiled. Although an informative illustration, this experiment can be considered a bit artificial. It might be more informative if the study high lighted how RNA velocity performs in scenarios where cells may be incom pletely sampled due to technical limitations (e.g. downsampling populations vs. complete removal).”

**Response:** Our main point with this figure is to illustrate the transition probability method enforces that the mapped (ie. low-dimensional) vector always points towards points (ie. cells) which are present in the specific embedding. Because of this visualization con straint, it is important to be cautious when interpreting mapped velocities, and this con straint forces the vector field to look consistent with the depicted cell population. We emphasize that the estimated high-dimensional velocities are unchanged between the different panels; it is only the low-dimensional embedding that changes.

The figure is not examining the question of how RNA velocity performs where a “cell fate state” or “terminal differentiation endpoint” is either not present or (more likely) represented at a very low proportion amongst the cells studied. For this question, we agree with the reviewer that down-sampling and re-estimating RNA velocity (including re-smoothing the data using the k-NN graph) would be a very appropriate analysis.

Based on this, we believe the reviewer might have misunderstood the point we’re trying to make. To clarify this for our audience, we now write the following in the “Transition probabilities impose directional constraints” section of the Results: “*We emphasize that the high-dimensional velocity estimates are unchanged between the panels.”* as well as “*Velocity transition probabilities cannot represent unseen parts of an expression state space, regardless of the direction of the high-dimensional velocity vectors.”*

“6. The authors should provide details on how the quantification is performed in Figure 2d in the methods section.”

**Response:** We add a new subsection “Comparison of directions of the cell-level vectors” in the Methods section, which reads as

“*In Figure 2d, we analyze the cell-level vector directions of 53 pre-endocrine cells, as identified within the annotated rectangle in Figures 2b and c. After mapping the high-dimensional RNA velocity data onto the UMAP embeddings, we compute the angles for each cell-level vector. To estimate the kernel density of the two circular variables, we employ the Von Mises Kernel, which is a circular normal kernel. We then use Watson’s two-sample test of homogeneity from the R circular package to compare the two circular variables.”*

“7. In reference to Figure 3, the authors describe a simulation experiment gen erating 500 cells and 10 genes with low noise levels. Why is it super important that the resulting qualitatively similar vector field has differences around the start of the trajectory?”

**Response:** It is not super important that there are differences at the start of the trajec tory, so we moved most of that analysis to the supplement. In addition, reviewing this section made us realize that part of the writing was unclear and we have tried to clarify this. Specifically, in this section, we are not analyzing RNA velocity estimates, but we are instead assessing to what extent the transition probability method results in a vector field that reflects the high-dimensional vector field. We start our manuscript with this analysis because it is important to understand how the embedding/visualization approach works.

As a result, we have substantially re-written the Results section titled “Visualizing veloc ity without noise”

“8. To illustrate how well projected RNA velocity vectors capture direction ality, the authors should compute the cosine similarity between (1) projected low dimensional vectors and high dimensional vectors and (2) transition prob ability low dimensional vectors and high dimensional vectors and (3) high dimensional dynamical velocity and the true velocity vectors for simulated data under low noise conditions, as in Figure 3. The latter is particularly im portant to mention as this is what RNA velocity is designed for.”

**Response:** Calculating cosine similarity directly between low-dimensional and high-dimensional vectors is infeasible due to the incompatible dimensions. Nevertheless, we can separately compare similarities within the low-dimensional and high-dimensional spaces. By calcu lating the cosine similarities between the estimated vectors and the true vectors in both the low- and high-dimensional spaces, we can assess how well the RNA velocity cap tures directionality in each space. These comparisons will be distinct and specific to their respective dimensional spaces.

For the simulations, we have now included two cosine similarities in both high- and low dimensions. In the new main Figure 5, we calculate the cosine similarity between the mapped low-dimensional cell-level velocity estimates and the mapped low-dimensional true cell-level velocity at various noise levels (additional noise levels in Supplementary Figure S5). Additionally, in the new Supplementary Figure 11c, we compute the cosine similarity between the estimated high-dimensional velocities and the true high-dimensional

velocities at different noise levels.

“9. PCC should be clearly defined and described in methods.”

**Response:** We have now explained how Pearson’s Correlation Coefficient (PCC) is calcu lated in the Methods section:

“*For two continuous variables, X and Y, the formula for Pearson’s Correlation Coefficient is given* 

*by:*

*r* =^∑^*^n^_i_*_=1_(*x_i_ − x*¯)(*y_i_ − y*¯) ^~~p~~^∑*^~~n~~^_i_*_=1_(*x_i_ − x*¯)^~~2~~^ ∑*^~~n~~^_i_*_=1_(*y_i_ − y*¯)^~~2~~^ 

*Here, x and* ¯ *y represent the mean values of variables X and Y, respectively, and n is the total* ¯ *number of paired observations.”*

“10. On page 2, lines 4-5, the authors mention that the RNA velocity vector field is used to provide evidence for the correctness of the embedding. This is further mentioned on Page 18, lines 21. Can you clarify / provide references where/how the visualized vector field is used to validate an embedding?”

**Response:** First, we clarify that this use of RNA velocity is not something that was rec ommended or suggested by either La Manno et al. (2018) nor Bergen, Lange, et al. (2020).

While the foundational papers did not use the RNA velocity vector fields as a means of validating a low-dimensional embedding, this has been done in other papers. An exam ple of this faulty line of reasoning is the description and interpretation of Figure 4 in Dura et al. (2022).

“11. On page 3, line 27: ’RNA velocity analysis results are usually qualitative’ - could you clarify how exactly this is validated in this qualitative manner?”

**Response:** The full quote the reviewer refers to is: ”Assessments of RNA velocity analy sis results are usually qualitative: does the resulting vector field visualization accurately capture known cellular dynamics of the biological system?”

We recognize that this was not clear language and we have re-written the paragraph for clarity:

“*Visualization of RNA velocity results through the construction of a low-dimensional vector field on a given embedding (optional component 2) is important for using RNA velocity to bring insight into a biological system. Much – but not all – of the evidence for the usefulness of RNA veloc ity rests on its ability to produce low-dimensional vector fields which appear to represent known biology accurately. This suggests that it is useful to understand the properties of this second com ponent; what happens when high-dimensional velocity estimates are mapped to a low-dimensional embedding, particularly using the transition probability method introduced by La Manno et al. (2018).”*

“12. On page 9, line 16: ’the kNN graph learned from the data is perturbed away from the true graph’ - what does true graph mean? Point related to this, the trueKNN, learned kNN, trans true kNN acronyms in Figure 4 could be defined in a clearer way.”

**Response:** Because it is indeed far from obvious what the “true k-NN graph” refers to, we now describe this in the Results (with additional details in Methods). We write

“*It is not obvious how to define the true structure. Here, we use the fact that we are simulating from a model which can be represented as “signal + noise” where “noise” is independent Gaussian (Methods). When using the term “true” k-NN graph, we generate the signal data and form a k-NN graph of the signal data prior to adding independent noise.”*

We have tried to improve our nomenclature in Figure 4 in response to this (and related comments from Reviewer #1).

“13. The introduction could benefit from a bit more organization to be more digestible for readers that are less familiar with the history and terminology used in the RNA velocity field. I think that the introduction could become much more digestible if it was broken into subsections (description of the tech nology, dynamical vs steady state model, inference techniques, possible deci sions or components of various inference techniques that you are planning to evaluate in your paper).”

**Response:** We have slightly expanded our description of the models in the start of our Results where we write: “*In the steady state model, a single parameter representing degradation rate is assumed different between genes, and this parameter is estimated using robust regression (La Manno et al., 2018). In the dynamic model, both transcription, splicing, and degradation rate are assumed to differ between genes, and these parameters are estimated together with a cell specific latent time for each cell using an EM algorithm (Bergen, Lange, et al., 2020). The original papers contain well-written expositions of the details.”*

In our manuscript, we don’t really evaluate any of the aspects mentioned by the reviewer. These aspects (model formulation, inference, etc) are well covered by the original papers as well as subsequent discussion of RNA velocity, particularly the work of Gorin et al. (2022) and Marot-Lassauzaie et al. (2022). In our view, adding an extended discussion of these aspects would make our paper substantially longer and not add much value precisely because we do not focus on these aspects. We acknowledge that our work – being a criticism of RNA velocity – requires some background in RNA velocity, but this background is better covered by the original papers and other references.

The parts of the RNA velocity analysis that we evaluate are – we believe – extensively covered in the Results. This is a bit unusual, but these parts of the workflow are not that well covered in the main texts of La Manno et al. (2018) and Bergen, Lange, et al. (2020).

“14. The discussion could benefit from a clearer explanation of where RNA velocity works and fails. It currently highlights speed limitations; however, the authors should highlight correctness of high dimensional directionality, speed, and parameter estimates under low noise conditions, as well as the correctness of directional mapping under low noise conditions. It seems that the limitation is with using a kNN graph that is not representative of the un derlying biological process (overwhelmed by noise, missing cell populations) vs RNA velocity estimation itself.”

**Response:** Above, in question 4, we have added sentences about the low-noise setting to the Results. Here we describe changes to the Discussion.

In the subsection titled “What influences the direction of RNA velocity?” “*At low noise levels (1-2), the cosine similarity between estimated and true directions is relatively high.”*

In the subsection titled “RNA velocity does not estimate expression speed” “*At low noise levels (1-2), there is a decent correlation between the true speed and the estimated speed.”*

and we have added a new small section titled “The impact of the noise level in our simu lations” where we write

“*We have utilized simulations to examine the performance of RNA velocity under varying noise levels and compared the results of using both the learned and true k-NN graphs. As these simu lations are based on the RNA velocity model, they represent an idealized best-case scenario. With a fixed signal in these simulations, they correspond to decreasing signal-to-noise situations. Our findings unequivocally demonstrate that the signal-to-noise ratio significantly influences RNA velocity performance. A critical aspect of this impact arises from the k-NN graph of the observed data, particularly through the k-NN smoothing step implemented during preprocessing. In low noise regimes (levels 1-2), RNA velocity exhibits decent to good performance. However, its effec tiveness quickly deteriorates at higher noise levels, where the learned k-NN graph deviates from the k-NN graph calculated on data before the addition of measurement errors. For noise levels 1-2, the measurement noise itself is unrealistically low (Figure 3a). Nonetheless, the crucial factor is likely the noise magnitude relative to the signal, which we have not attempted to quantify in real data. Quantifying this aspect is challenging because it depends on the magnitude of structured expression changes across the cell population being investigated. Consequently, we believe that determining how to quantify signal-to-noise in single-cell data and examining its impact on the learned k-NN graph is an essential open question. This inquiry is particularly significant since the k-NN graph is a central component in many single-cell analysis methods.”*

“Minor comments and typos:

1. Page 2, lines 9-10: ”the manuscript primarily discusses the python imple mentation of velocyto.” Page 3, lines 44-46: ”here, we primarily focus on the newer dynamical model as implemented in scVelo”. A distinction should be made between how spliced and unspliced counts are being quantified (e.g. ve locyto pipeline) vs. how RNA velocity is being estimated (e.g. scVelo steady state or dynamical model). Furthermore, it would be useful to explain what is happening in velocyto and not just that the manuscript discusses a python implementation.”

**Response:** We are now making it clear in the Methods that we are following the scVelo implementation when it comes to quantification (our work does not really go into the quantification aspect of the workflow, so we are somewhat brief about that aspect). Par ticularly, we have accessed processed data supplied by a variety of authors, predomi nately from the scVelo package. Our simulations – borrowed from the setup introduced in Bergen, Soldatov, et al. (2021) – are not affected by pre-processing. We added the fol lowing comment to the 1st Results section

“*Recent work has shown the importance of the choices of how to quantify spliced and unspliced counts (Soneson et al., 2021); here, we largely follow how each dataset was originally processed (Methods).”*

The reviewer is also asking for a detailed description/discussion of the steady state method as described by La Manno et al. (2018). We have decided not to do this in our main text for reasons of complexity. La Manno et al. (2018) contains two different implementations: python and R. Particularly, the R implementation is quite different from the description in the manuscript, particularly with regards to the transition probability method, which is only apparent when reading the R code. We are discussing some of the differences in detail in Supplementary Note 3.1. Our discussion of the steady state model in the main text (like the discussion in Bergen, Lange, et al. (2020) is actually about the Python im plementation, so we state this for reasons of precision, even if it may seem like a weird comment.

“2. The acronym kNN is used frequently throughout the text and is never formally defined. It is best to define this as k nearest neighbor graph.”

**Response:** We now define the k-NN as the acronym for k-Nearest-Neighbors in its first appearance.

“3. In the ’construction of the k-NN graph’ section in the methods on page 19, lines 56-57, the authors should describe what the default parameters are. How many neighbors were considered? This is mentioned in the supplemental file (k = 30), but should be also included in the main text for clarity.”

**Response:** We now write in the Methods section (Subsection “Real scRNA-seq datasets filtering and normalization”, sub-subsection “Construction of the k-NN graph”):

“*Given there is no guide on choosing the k in a new dataset, we have consistently used the k* = 30*, the default in the scVelo package, unless otherwise specified.”*

“4. Abstract, line 37, ’This dependence lead’ → ’This dependence leads to’” **Response:** Thanks

“5. Page 2, line 47: ’highlight examples where RNA velocity vector is not compatible’ → ’highlight examples where the RNA velocity vector is not com patible’”

**Response:** Thanks.

“6. Figure 4g: ’consine similarity’ → ’cosine’”

**Response:** Thanks.

**References**

Bergen, V., M. Lange, S. Peidli, F. A. Wolf, and F. J. Theis (2020). “Generalizing RNA veloc ity to transient cell states through dynamical modeling”. *Nature Biotechnology* 38, pp. 1– 7. DOI: 10.1038/s41587-020-0591-3.

Bergen, V., R. A. Soldatov, P. V. Kharchenko, and F. J. Theis (2021). “RNA velocity—current challenges and future perspectives”. *Molecular Systems Biology* 17.8, e10282. DOI: 10. 15252/msb.202110282.

Dura, M., A. Teissandier, M. Armand, J. Barau, C. Lapoujade, P. Fouchet, L. Bonneville, M. Schulz, M. Weber, L. G. Baudrin, S. Lameiras, and D. Bourc’his (2022). “DNMT3A dependent DNA methylation is required for spermatogonial stem cells to commit to spermatogenesis”. *Nature Genetics* 54.4, pp. 469–480. DOI: 10.1038/s41588- 022- 01040-z.

Gorin, G., M. Fang, T. Chari, and L. Pachter (2022). “RNA velocity unraveled”. *bioRxiv*, p. 2022.02.12.480214. DOI: 10.1101/2022.02.12.480214.

La Manno, G., R. Soldatov, A. Zeisel, E. Braun, H. Hochgerner, V. Petukhov, K. Lid schreiber, M. E. Kastriti, P. Lonnerberg, A. Furlan, J. Fan, L. E. Borm, Z. Liu, D. v. ¨ Bruggen, J. Guo, X. He, R. Barker, E. Sundstrom, G. Castelo-Branco, P. Cramer, I. Adameyko, ¨ S. Linnarsson, and P. V. Kharchenko (2018). “RNA velocity of single cells”. *Nature* 560.7719, pp. 494–498. DOI: 10.1038/s41586-018-0414-6.

Marot-Lassauzaie, V., B. J. Bouman, F. D. Donaghy, and L. Haghverdi (2022). “Towards reliable quantification of cell state velocities”. *bioRxiv*, p. 2022.03.17.484754. DOI: 10. 1101/2022.03.17.484754.

Soneson, C., A. Srivastava, R. Patro, and M. B. Stadler (2021). “Preprocessing choices affect RNA velocity results for droplet scRNA-seq data”. *PLOS Computational Biology* 17.1, e1008585. DOI: 10.1371/journal.pcbi.1008585.

Weiler, P., K. V. d. Berge, K. Street, and S. Tiberi (2021). “A guide to trajectory inference and RNA velocity”. *bioRxiv*, p. 2021.12.22.473434. DOI: 10.1101/2021.12.22.473434.

**2^nd^ round**

**Reviewer 1**

The authors have satisfactorily addressed all of my concerns. I find the revised manuscript to be much improved and appreciate the work that has gone into enhancing the clarity of both the language and mathematical notation.

A few minor notes:

Be consistent with hyphenating the phrase "velocity-flavored".

The footnote regarding terminology is awkwardly formatted.

In Figure 3a, I think the y axis on the second plot should be "Spliced_g" rather than "Spliec_g".

**Reviewer 2**

Thanks to the authors for addressing all of my concerns. Great job with the revision and the paper is much stronger and clearer in this iteration.
